# Furan–Urethane Monomers for Self-Healing Polyurethanes

**DOI:** 10.3390/polym17141951

**Published:** 2025-07-16

**Authors:** Polina Ponomareva, Zalina Lokiaeva, Daria Zakharova, Ilya Tretyakov, Elena Platonova, Aleksey Shapagin, Olga Alexeeva, Evgenia Antoshkina, Vitaliy Solodilov, Gleb Yurkov, Alexandr Berlin

**Affiliations:** 1 Semenov Institute of Chemical Physics, Russian Academy of Sciences, 119991 Moscow, Russia; lokyaevazal@gmail.com (Z.L.); tretiakovi.v@yandex.ru (I.T.); e-o-platonova@yandex.ru (E.P.); vital-yo@yandex.ru (V.S.); berlin.a@yandex.ru (A.B.); 2 NTI Center “Digital Materials Science: New Materials and Substances”, Bauman Moscow State Technical University, 2nd Baumanskaya Street 5, 105005 Moscow, Russia; dzakharova@bmstu.ru; 3 Frumkin Institute of Physical Chemistry and Electrochemistry, Leninsky Prospect 31, Building 4, 119071 Moscow, Russia; shapagin@mail.ru; 4 N.M. Emanuel Institute of Biochemical Physics, Russian Academy of Sciences, Kosygina Street, Building 4, 119334 Moscow, Russia; alexol@yandex.ru; 5 A.N. Nesmeyanov Institute of Organoelement Compounds, Russian Academy of Sciences, Vavilova Street 28, Building 1, 119334 Moscow, Russia; antoshkina.ep@phystech.edu

**Keywords:** Diels–Alder reaction, polyurethane, self-healing, remendability

## Abstract

The repair efficiency of various self-healing materials often depends on the ability of the prepolymer and curing agent to form mixtures. This paper presents a synthesis and study of the properties of modified self-healing polyurethanes using the Diels–Alder reaction (DA reaction), obtained from a maleimide-terminated preform and a series of furan–urethane curing agents. The most commonly used isocyanates (4,4′-methylene diphenyl diisocyanate (MDI), 2,4-tolylene diisocyanate (TDI), and hexamethylene diisocyanate (HDI)) and furan derivatives (furfurylamine, difurfurylamine, and furfuryl alcohol) were used as initial reagents for the synthesis of curing agents. For comparative analysis, polyurethanes were also obtained using the well-known “traditional” approach—from furan-terminated prepolymers based on mono- and difurfurylamine, as well as furfuryl alcohol and the often-used bismaleimide curing agent 1,10-(methylenedi-1,4-phenylene)bismaleimide (BMI). The structure and composition of all polymers were studied using spectroscopic methods. Molecular mass was determined using gel permeation chromatography (GPC). Thermal properties were studied using TGA, DSC, and TMA methods. The mechanical and self-healing properties of the materials were investigated via a uniaxial tensile test. Visual assessment of the completeness of damage restoration after the self-healing cycle was carried out using a scanning electron microscope. It was shown that the proposed modified approach helps obtain more durable polyurethanes with a high degree of self-healing of mechanical properties after damage.

## 1. Introduction

Polyurethanes and their composites are widely used for various purposes, from the creation of structural materials to the manufacture of household items. However, one of the main problems associated with the use of this type of materials remains their subsequent disposal and recycling at the end of their service life. The disposal of polymeric materials by incineration is very common [1]; however, this method causes serious harm to the environment. There are also a number of other chemical recycling methods, such as hydrolysis, glycolysis, hydrogenation, and pyrolysis. For example, the application of glycolysis to polyurethane materials results in the release of polyols—soft segments of the polymer chain—for reuse [2]. In some cases, the use of mechanical methods is allowed; the spent polymer material is crushed and then used as a filler to create various types of composites [3,4]. There are various approaches to solving the problem of increasing polymer waste and related disposal problems, such as the development of new biodegradable polymers [5]. Extending the service life of such materials could in turn reduce the amount of waste. In the last decade, researchers have been increasingly interested in creating materials with the ability to self-heal mechanical damage in response to irritants [6,7]. The self-healing effect increases the service life of the product or enables easy recycling. Self-healing can be accomplished in several different ways. In so-called external systems, it occurs due to microcapsules containing a liquid monomer and catalyst embedded in the polymer; here, cases of purely physical methods of recovery, such as interdiffusion or phase-separated morphologies, should be excluded [8]. In intrinsic systems, healing occurs due to the presence of special structural fragments in the material, which, under the influence of heating or radiation, can undergo reversible splitting [9,10]. One of the main advantages of intrinsic systems compared to external ones is the unlimited number of healing attempts.

In the process of internal self-healing, various chemical processes can occur, such as the formation of hydrogen bonds [7], the emergence of disulfide bridges [11] or metal complexes [12,13], as well as the reversible Diels–Alder reaction (DA reaction) [9,14]. Various combinations of the above approaches are also possible. The DA reaction, which involves the interaction of furan (diene) and maleimide (dienophile) to form a DA adduct, is convenient for creating self-healing materials. The healing process involves a two-stage heating. First, a reverse Diels–Alder reaction (rDA) occurs at a given temperature, with the adducts splitting into the initial furan and maleimide, and then, at a lower temperature, a direct reaction occurs with the re-formation of adducts. This method is widely used to create thermally induced self-healing polyurethanes (PUs). Such PUs are usually obtained by synthesizing a prepolymer with terminal furan groups and adding a bismaleimide-based curing agent.

**Figure 1:**
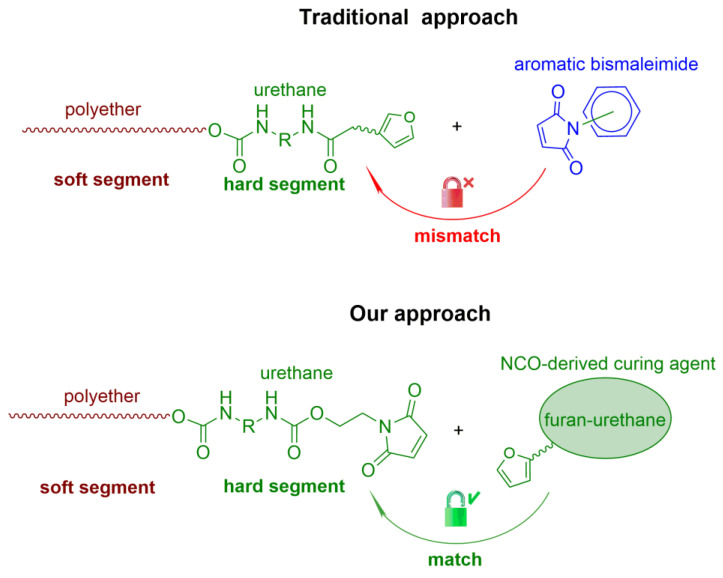
Schematic representation of the proposed approach.

However, there is the problem of reduced self-healing efficiency in the creation of self-healing polyurethanes, which is associated with the low affinity of bismaleimide to the furan-containing preform. We have already encountered the problem of incomplete restoration of mechanical properties [15,16] in the creation of self-healing PUs using the DA reaction. PUs are two-phase systems in which the “hard” segments are formed from urethane-containing fragments, and aliphatic esters form the “soft” ones. For the DA reaction to occur, it is necessary to concentrate active furan and maleimide groups in one segment [17]. To solve this problem, we used the “like dissolves like” method. To improve the affinity between the prepolymer and the curing agent, we obtained a series of furan-containing monomers based on the three main known isocyanates (TDI, MDI, and HDI). It is assumed that such furan–urethanes will mix better with the PU prepolymer and facilitate DA interactions [18]. In addition, the presence of urethane bonds in the monomers can contribute to an additional increase in the strength of the material, due to the formation of hydrogen bonds with urethane fragments in the PU prepolymer. An experimental PU composition was created based on one of the monomers. Its properties were compared with those of the material obtained using the traditional method [19,20,21,22], which involves a furan-containing prepolymer and a bismaleimide curing agent. The material obtained using the modified synthetic approach showed a higher molecular weight compared to traditional PU, and it was also more effective in restoring the elastic modulus and tensile strength. The synthetic approach is shown in Figure 1. Thus, we combined chemical and physical approaches to self-healing [8]; specifically, we obtained polymeric self-healing polyurethanes in which several self-healing mechanisms can be implemented, such as reversible covalent interactions (via the Diels–Alder reaction) and hydrogen bonds.

This work presents the synthesis and study of the structure of self-healing polyurethanes obtained from all furan–urethane monomers. A comparative assessment of the thermal, mechanical, and self-healing properties of modified materials with traditional PUs of similar structure was carried out. The developed approach will enable the flexible regulation of self-healing polyurethanes’ properties, significantly expanding their application scope and supplementing knowledge in the field of self-healing via the Diels–Alder reaction materials.

## 2. Materials and Methods

### 2.1. Materials

Furan–urethane monomers were prepared and characterized in previous work [18]. Difurfurylamine (DFA) was prepared by a modified literature method [23]. N-(2-hydroxyethyl)-maleimide (HEMI) was synthesized via a reported procedure [24]. Methylene diphenyl diisocyanate (MDI) 98%, 1,10-methylenedi-1,4-phenylene)bismaleimide (BMI) 95%, hexamethylene diisocyanate (HDI) 99%, and Tin(II) 2-ethylhexanoate (Sn(Oct)_2_) were purchased from Sigma-Aldrich (Steinheim, Germany) and used as received. TDI (toluene-2,4-diisocyanate, 80% of 2,4-isomer) was purchased from Sigma-Aldrich (Steinheim, Germany), distilled in a vacuum, and frozen out to separate the 2,6-isomer. Furfuryl alcohol (FOH) 98% was purchased from Sigma-Aldrich (Steinheim, Germany) and distilled under reduced pressure to remove tarry material. Furfurylamine (FA) 99% was purchased from Acros (Geel, Belgium), dried over NaOH, and distilled in a vacuum prior to use. PPG-2000 (polypropylene glycol, Mn = 2000) was purchased from Sigma-Aldrich (Steinheim, Germany) and dried under vacuum at 110 °C prior to use. N,N-dimethylformamide (DMF) was purchased from Acros (Geel, Belgium), dried over CaH_2_, and distilled in a vacuum prior to use. Methylene chloride (CH_2_Cl_2_) was purchased from Acros (Geel, Belgium), dried over P_2_O_5_, and distilled.

### 2.2. Characterizations of Samples

^1^H NMR spectra were obtained on a Bruker Avance 600 NMR Spectrometer (600.1 MHz) (Brno, Czech Republic) using the residual proton signal of deuterated solvent as a reference. Chemical shifts were reported as parts per million downfield from tetramethylsilane (TMS). ATI-FTIR was performed on a Nicolet iS10 spectrometer (Thermo Fisher Scientific, Waltham, MA, USA) in the range of 4000 to 650 cm^−1^ on a germanium crystal. Gel permeation chromatography (GPC) analysis was carried out on a chromatographic system: high-pressure pump STAYER p.2 (Akvilon, Podolsk, Russia), detector refractometer Smartline RI 2300 (KNAUER, Berlin, Germany), and thermostat JETSTREAM 2 PLUS (KNAUER, Berlin, Germany). Temperature 40 ± 0.1 °C, eluent THF, flow rate 1.0 mL/min. Columns 300 × 7.8 mm, Phenogel sorbent (Phenomenex, Torrance, CA, USA), 5 µm, pore size from 50 Å to 105 Å. Molecular weights were estimated against polystyrene standards. Thermogravimetric analysis (TGA) was carried out on a NETZSCH TG 209 F1 Libra (Selb, Germany) device in the temperature range from 30 to 550 °C at a heating/cooling rate of 10 K min^−1^ in an argon atmosphere. The thermal behavior was studied by DSC on a NETZSCH DSC 204 F1 Phoenix instrument (Selb, Germany) in the temperature range from −80 to 180 °C at a heating/cooling rate of 10 Kmin^−1^ in an argon atmosphere. Polyurethanes based on tetrafuranic monomers were additionally investigated for the presence of tandem Diels–Alder adducts in their structure. The temperature program consisted of two heating/cooling cycles at a rate of 10 Kmin^−1^; the first heating was carried out from 20 to 180 °C, the second from 20 to 300 °C. A sample weighing about 18 mg was taken for the study. Thermomechanical analysis was performed on TA Instruments TMS Q400E (New Castle, DE, USA) in the penetration mode. Samples with a diameter of 6 mm were tested in the temperature range from −70 to 250 °C at heating/cooling rates of 5 K/min, a load of 1 N, and a probe diameter of 2.54 mm. Cracks in the polymer film were studied using a JSM 6060A scanning electron microscope (Jeol, Tokio, Japan) at an accelerating voltage of 15 keV. The image was formed in secondary electrons. The samples were films of the research objects, 1 mm thick, square in shape with a side of 10 mm. To obtain a conductive surface, thin carbon was thermally sprayed onto the samples in a Coating System E306A vacuum station (Edwards, Burgess Hill, UK). Sample preparation and analysis were carried out in 2 stages. In the first stage, linear mechanical defects were applied to the samples with a blade at an angle of 30 degrees when the blade was immersed to a depth of 50–100 μm, carbon was sprayed, and SEM images of mechanical surface defects were obtained. In the second phase, the samples were thermostated at 120 °C for 90 min, then at 60 °C for 24 h, and morphological changes on the surface of the samples in the area of applied defects were studied. The uniaxial tension test was carried out using a Zwick Roell Z100 universal testing machine (Ulm, Germany). Ten dumbbell-shaped samples were cut from each polyurethane film. To quantify the effectiveness of the self-healing process, the samples were divided into two groups. Five samples were tested immediately, while the remaining five were damaged and thermocycled according to the same principle as the samples for SEM. The tests were carried out at a temperature of 25 °C with a loading speed of 50 mm/min. High-resolution mass spectra were collected with a liquid chromatography–mass spectrometer LCMS-9030 Q-TOF (Shimadzu, Kyoto, Japan) with electrospray ionization and a quadrupole-time-of-flight tandem mass analyzer in positive and negative ion modes (*m*/*z* from 50 to 1500); mass-scale was calibrated using a solution of sodium iodide in a mixture MeOH/H2O (1:1 *v*/*v*). The mobile phase was acetonitrile (99.9+%, HPLC Gradient grade, Carlo Erba, Milano, Italy) with a flow rate of 0.4 mL/min. The electrospray voltage was 4.0 kV in the positive ion mode and 3.5 kV in the negative ion mode. The interface temperature and the temperatures of the desolvation line and the heating block were 100, 250 and 400 °C, respectively. Nitrogen (99.5%) was used as drying and heating gas with a flow rate of 10 L/min and as nebulizing gas with a flow rate of 3 L/min.

### 2.3. Preparation Process of HEMI-TDI-PPG Prepolymer

The polyurethane prepolymer was obtained using a two-step procedure [18]. In the first step, PPG-2000 (70.00 g, 1 equiv) was added to TDI (12.19 g, 2 equiv). The reaction solution was stirred for 1.5 h at 60 °C in an argon atmosphere. The progress of the reaction was monitored by IR spectroscopy by the disappearance of the characteristic band at 3500 cm^−1^ (OH group). After the reaction was completed, the mixture was cooled to room temperature and HEMI (9.88 g, 2 equiv) in 10 mL of DMF was added. Afterwards, the reaction mixture was stirred at 60 °C for 1.5 h in an argon atmosphere until the characteristic band of the NCO group at 2270 cm^−1^ completely disappeared in the IR spectrum [25,26].

### 2.4. Synthesis of PU-DA-Modified

Furan–urethane monomers based on TDI, HDI and HDI were obtained using the method presented in our previous work [18]. The composition of the furan–urethane monomers was confirmed by HR-MS spectroscopy (Figure S1a–h). A solution of the corresponding furan-containing agent (H-, T- and M-series) in DMF (1.0 g/mL) was added to a solution of the HEMI-TDI-PPG prepolymer in DMF (0.5 g/mL). The molar ratio of the furan–urethane monomer to the prepolymer was [furan–urethane monomer]:[prepolymer] = 1:1. The resulting solution was stirred at 60 °C for 2 h [18].

### 2.5. Synthesis of PU-DA-Traditional

Traditional PUs were obtained using a standard two-stage procedure [15,27,28]. In the first stage, (NFu_2_)_2_-TDI-PPG, (NFu)_2_-TDI-PPG and (OFu)_2_-TDI-PPG prepolymers were obtained from TDI, PPG-2000 and furan chain extenders (DFA, FA and FOH). In the second stage, a solution of BMI in DMF (1 g/mL) was added to a solution of the prepolymer in DMF (0.5 g/mL) in the molar ratio [BMI]:[prepolymer] = 1:1. The reaction solution was stirred at 60 °C for 2 h.

### 2.6. Film Preparation

A solution of the polymer in DMF (1.3 g/mL) heated up to 60 °C was poured into a Petri dish and kept in an oven at 60 °C for 48 h.

## 3. Results and Discussion

### 3.1. Synthesis and Characterization of Polyurethanes

**Figure 2:**
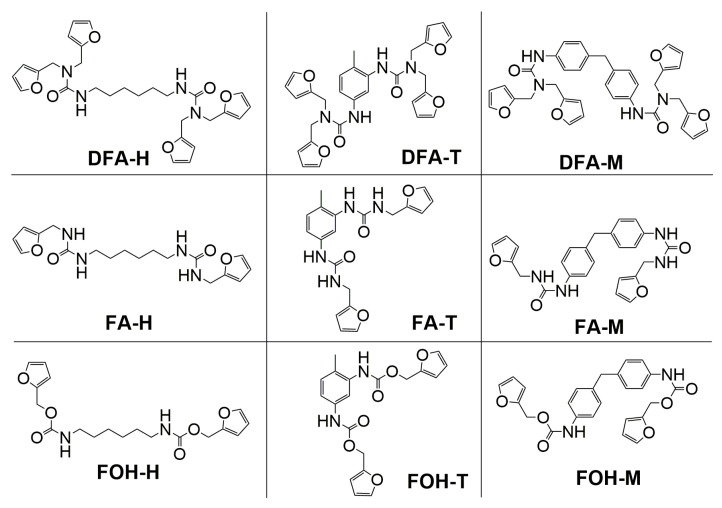
Furan–urethane monomers.

In our previous work on the self-healing of PUs using the DA reaction [18], it was suggested that the peculiarities of the active group distribution in the polymer domains may cause the incomplete restoration of mechanical properties. Specifically, the aromatic bis-maleimide curing agent is concentrated predominantly in the hard domains, whereas the furan groups of the prepolymer are mainly located in the soft domains. Due to this unevenness, the furan rings are «locked» and are inaccessible for interaction with maleimide. However, the use of curing agents similar in structure to the hard domain of PU can solve this problem. Such curing agents can be more effectively mixed with the isocyanate component of the polymer, which will contribute to a more complete DA reaction and, accordingly, increase the efficiency of self-healing. Thus, di- and tetrafuranurethane curing agents were obtained from the main known isocyanates (TDI, MDI, and HDI) and furan derivatives (DFA, FA, and FOH) (Figure 2). A model polymer was obtained based on one of the hardeners (FA-T) and a maleimide-terminated PU prepolymer. The resulting polymer was then compared with a sample synthesized with the commonly used “traditional” approach via interaction of a furan-terminated prepolymer with a low-molecular-weight bismaleimide curing agent. The modified PU outperforms the “traditional” one in terms of MM (M_w_ = 19,200 Da versus 5000 Da for the traditional PU) and the efficiency of the mechanical property restoration (the recovery efficiency was 93% versus 54% for the traditional PU).

In this work, polymers derived from other hardeners were also synthesized to study the influence of the furan–urethane chemical structure on the thermal behavior of polyurethanes. The furan monomers differ in the number of active groups, the structure of the spacer between the furan groups, and also in heat resistance. For comparison, PU samples were also obtained via the traditional method. Aromatic bismaleimide BMI was chosen for the synthesis of traditional PUs, since it is the most common component to obtain self-healing by the DA reaction polyurethanes. Thus, the resulting polymers can be compared not only with model PUs but also with polymers from other published works.

**Scheme 1:**
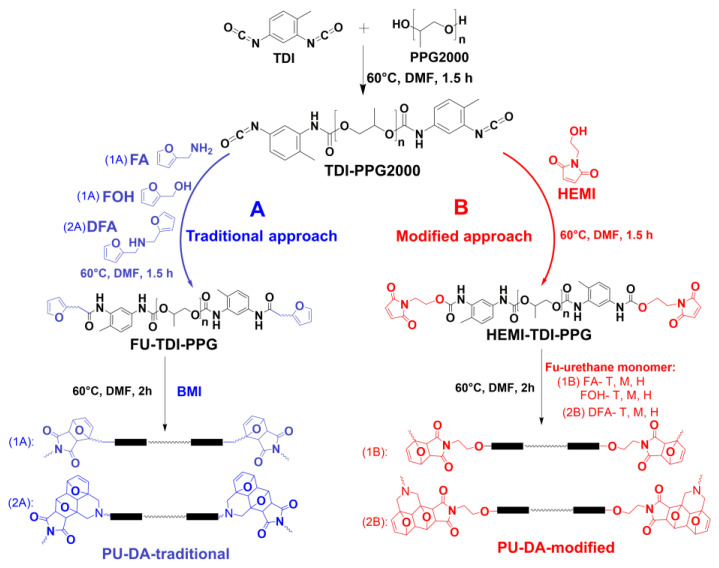
Synthesis of PU-DA polymers: A—traditional approach, B—modified approach.

Both synthetic approaches, modified and traditional, are presented in Scheme 1. PU-DA was synthesized with a two-step method. In the first step, an isocyanate-terminated prepolymer was prepared from PPG-2000 macrodiol (soft segment) and TDI (hard segment). Afterwards, it reacted with chain extenders: HEMI in the modified method, or DFA, FA, and FOH in the traditional method. As a result, oligomers with terminal maleimide (HEMI-TDI-PPG) or furanic (Fu-TDI-PPG) groups were formed. At the second stage, the resulting prepolymers were cured via the DA reaction with furan–urethane curing agents (for polymers of the T, H and M series) or low-molecular-weight BMI (polymers PU-(NFu_2_)_2_, PU-(NFu)_2_, and PU-(OFu)_2_). The molar ratio [prepolymer]:[curing agent] = 1:1 was used. The introduction of difuranic derivatives (furan–urethane curing agents or oligomer with terminal furanic groups) into the Diels–Alder reaction leads to the formation of classic adducts of the Diels–Alder reaction, whereas the use of tetrafuranic derivatives such as DFA-H, DFA-T, DFA-M, DFA-TDI-PPG leads to the formation of tandem adducts of the Diels–Alder reaction (Scheme 1), as was shown in [29,30,31].

**Figure 3:**
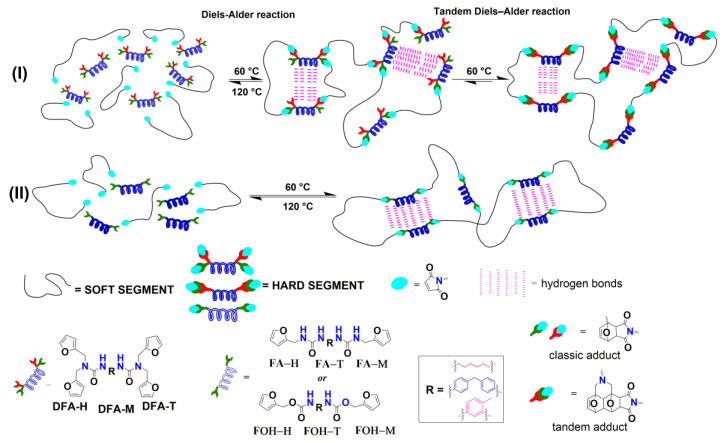
Schematic view of a reversible covalent and physical bonds formed during the curing of a prepolymer with furan–urethanes: (**I**)—reversible bonds based on tetrafuranurethanes; (**II**)—reversible bonds based on difuranurethanes.

All synthesized polymers, except PU obtained from FOH derivatives (Table 1), had the appearance of transparent yellow films, soluble in THF, DMF, and DMSO. FOH-based polymers, regardless of curing time, appeared as sticky, weak films. We believe that the presence of alcohol-containing monomers in the polyurethane structure leads to weak microphase separation, which reduces the density of the physical network of hydrogen bonds, which is crucial for the mechanical strength. Figure 3 shows a detailed scheme of reversible covalent interaction (formation of classic and tandem DA adducts) and hydrogen bonds formed during the curing of polyurethanes, depending on the furan–urethane monomers.

**Figure 4:**
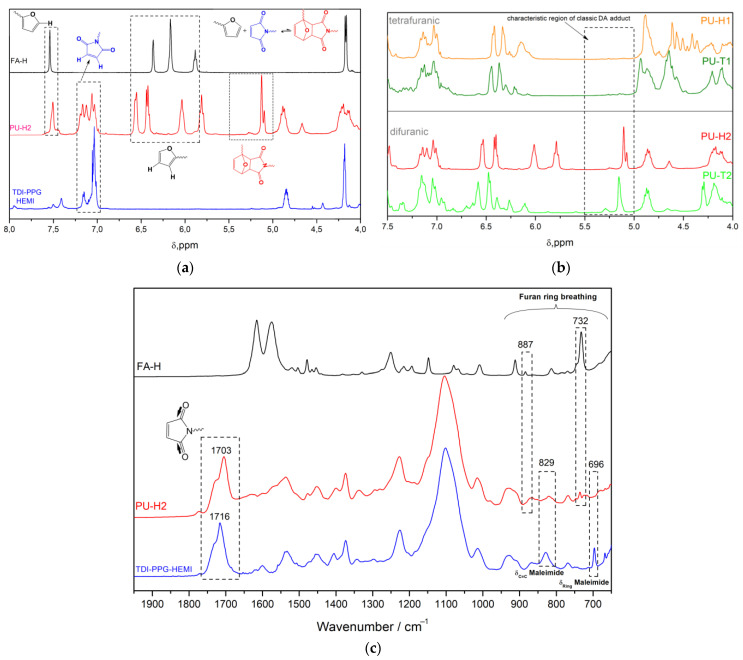
Spectra: (**a**) NMR spectra of PU-H2, prepolymer and curing agent; (**b**) NMR spectra of tetrafuranic and difuranic PU; (**c**) IR spectra of PU-H2, prepolymer and curing agent.

All synthesized polymers were characterized by ^1^H-NMR and IR spectroscopy. Figure 4 shows the spectra of the furan-containing agent FA-H, the TDI-PPG-HEMI preform, and the PU-H2 polymer. The spectra of the other polymers are presented in the Supplementary Materials (Figure S2a–t). The ^1^H-NMR spectra of the prepolymer and the resulting PU showed that the intensity of the signals of double-bond protons of the maleimide cycle, 7.04–7.05 ppm, decreased during the reaction, indicating the successful formation of DA adducts (Figure 4a). A noticeable decrease in the signals of furan protons at 7.55, 6.37, and 5.90 ppm and the appearance of signals corresponding to the formation of a DA adduct also indicate a successful interaction between the monomers and the prepolymer [28]. The characteristic area of ^1^H-NMR spectra for polymeric materials with classic DA adducts is the region of 5.09–5.38 ppm (Figure 4b), as was previously shown in [29]; however, for polymeric materials with tandem adducts, the region corresponding to protons at the oxygen bridge is at 4.55–4.95 ppm. According to the literature, ^1^H-NMR spectroscopy is a reliable method for identifying the type of the obtained material for both types of classic and tandem adducts. However, we have shown that for the obtained polymeric materials, the signals corresponding to the methylene groups of the HEMI-TDI-PPG and DFA-TDI-PPG prepolymers overlap with the characteristic region of protons at the oxygen bridge for tandem adducts. It is known that the formation of tandem adducts by the DA reaction proceeds through the formation of intermediate classic adducts. Consequently, the degree of conversion of classic adducts into tandem ones for the materials PU-H1, PU-M1, PU-TI, and PU-(NFu_2_)_2_ can be estimated by the absence of signals in the characteristic region of 5.09–5.38 ppm in the ^1^H-NMR spectra, which was done in this work. To verify the proposed approach, kinetic NMR experiments were performed (Figure S2u). The starting components HEMI-TDI-PPG and DFA-H were dissolved in DMSO-d^6^ in a 1:1 ratio and then placed in an ampoule for recording of ^1^H-NMR spectra at 60 °C every 10 min for two hours. As a result, after an hour of the experiment, the concentration of classic endo- and exo-adducts reaches a plateau, and by the time the reaction is complete, the target polyurethane materials do not contain classic DA adducts, which is consistent with the literature data [31].

In all samples (PU-H1, PU-M1, PU-TI, PU-(NFu2)2), polymers exclusively derived from tandem Diels–Alder adducts were achieved (Figure 4b and Figure S2c,i,o).

The interaction between the curing agent and the prepolymer can also be detected in the IR spectra (Figure S3a–l, Supplementary Materials). According to IR spectroscopy (Figure 4c), the characteristic bands of asymmetric vibrations of C=O groups conjugated with the maleimide ring in the spectra of the prepolymer (1716 cm^−1^) and PU (1703 cm^−1^) were preserved. A small red shift of the band may be caused by the breaking of the C=O and maleimide heterocycle conjugation via the DA reaction occurring [32]. Moreover, all synthesized polyurethanes contain bands of terminal maleimide groups: the band of the double bond (C=C, 829 cm^−1^) and the band of bending vibrations of the maleimide ring (696 cm^−1^), as well as characteristic bands of the furan ring vibration at 887–884 cm^−1^ and 748–732 cm^−1^. The presence of characteristic bands in the IR spectra corresponding to terminal maleimide and furan groups proves that there are no side processes involving these functional groups, which is usually accompanied by the removal of components from the reaction zone.

Determination of molecular mass distribution by GPC showed that the molecular weight characteristics of the modified samples are characterized by a wide polydispersity (polydispersity index > 1.4). Almost all samples show a unimodal molecular weight distribution, only in some cases, some bimodality is observed. All traditional samples were characterized by a wide bimodal molecular weight distribution, significantly exceeding that of the modified samples (Table 2).

### 3.2. Thermal Properties

**Figure 5:**
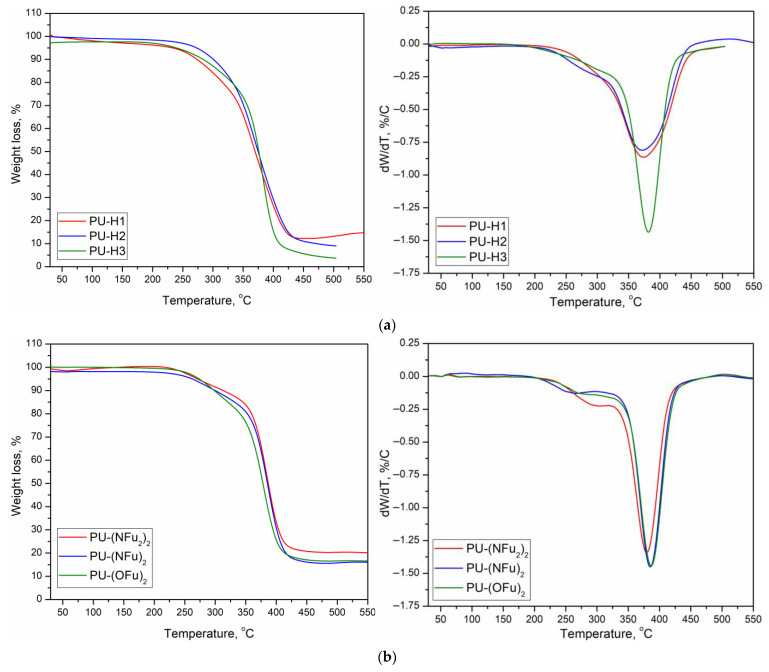
TGA (left) and DTG (right) curves of PU samples: (**a**) modified (PU-H-series); (**b**) traditional.

The thermal stability of all self-healing polyurethanes was investigated by thermogravimetric analysis (Figure S4a–l, Supplementary Materials). The previously determined decomposition temperatures, T_5%_ and T_10%_, of difurfurylamine derivatives and furfuryl alcohol are quite close in value, while furfurylamine derivatives have significantly higher decomposition onset temperatures (in the case of furan-containing agents of the T- and H-series, the difference was almost 100 °C) [18]. In this regard, it could be expected that self-healing polyurethanes derived from these agents would have greater thermal stability; however, according to the obtained TGA data, no specific trend was observed in the thermal stability values for modified PUs from different series (as was in the case of the di- and tetrafuran–urethane curing agents). We assume that the thermal stability of polymers strongly depends on the stability of the prepolymer and the furan hardener. Thus, in the T- and H-series furan–urethanes, furfuryl alcohol derivatives are the least thermally stable [18], and the same trend was observed for polymers obtained from these hardeners. In the M-series, the least stable was furan–urethane from furfurylamine—and the same thermal behavior was demonstrated by the PU synthesized from this curing agent. The modified polyurethanes of the H-, T- and M-series have the same decomposition pattern and are stable at temperatures up to 207–278 °C. As an example, Figure 5 shows the curves of the H-series samples. The maximum decomposition rate of the more heat-resistant preform structure corresponds to the temperature range of 226–383 °C. Losses are 71–91% for samples PU-H1–PU-T3, respectively. Traditional PU samples were stable at temperatures up to 247–278 °C, and the polymeric preform was stable at temperatures up to 286–314 °C (68–75% loss for PU-(NFu_2_)_2_ and PU-(OFu)_2_, respectively), which does not differ significantly from the thermal stability of modified PU samples. However, it was found that polymers derived from tandem DA reaction adducts (for the PU-Hn, PU-Tn series) are more thermally stable compared to polymers from classic DA reaction adducts. Comparable thermal stability is observed for the products PU-(NFu_2_)_2_ and PU-(OFu)_2_. The data is presented in Table 3.

**Figure 6:**
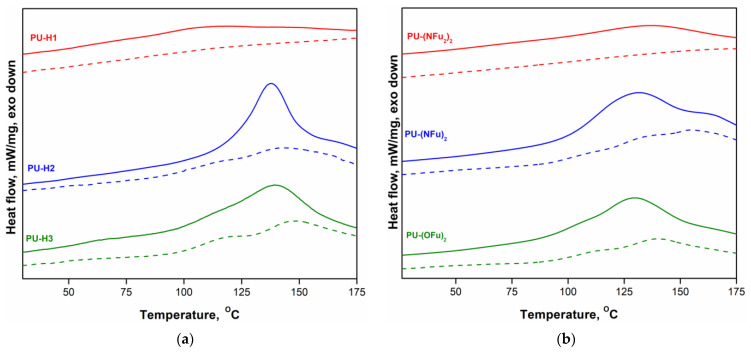
DSC curves of PU samples: (**a**) modified (PU-H-series); (**b**) traditional (solid lines—first heat, dashed lines—second heat).

All polymers were also examined by differential scanning calorimetry (DSC) (Figure 6). A comparative analysis of the DSC curves of the PU-H series and traditional PU samples in Figure 4 was carried out; curves of other polymers are given in Supplementary Materials (Figure S5a–l).

The glass transition temperatures for the modified polymers were in the range from −50 to −38 °C for the soft segment (Tg_ss_) [15,18,33]. The transition in the range from −4 to 7 °C can be attributed to the Tg of the hard phase (Tg_hs_) [34,35,36].

Tg_ss_ for traditional PUs were in the range from −56 to −51 °C, and Tg_hs_ from −7 to −4 °C (Figure S5j–l). The traditional samples are characterized by a low degree of phase separation due to the greater thermodynamic compatibility of the hard and soft blocks in their structure, and the miscibility of these phases increases when moving from the PU-(NFu_2_)_2_ to the PU-(OFu)_2_ (Table 4).

In the case of the modified PU-DAs, the nature of the diisocyanate in the structure of the furan-containing chain extenders had a significant effect on the polyurethane microstructure; from the PU-H to the PU-T and PU-M series samples, an increase in the Tg_ss_ values was observed, indicating a gradual segregation of the hard blocks into a separate phase due to the growth of thermodynamic incompatibility. In addition, the decrease in the difference between the Tg_ss_ and Tg_hs_ values observed in the modified polymers when moving from the difurfurylamine-derived samples to the furfurylamine and furfuryl alcohol derivatives indicates an enhancement in the phase miscibility for samples numbered 2 and 3 in the series [34]. It should be noted that the FOH-derived PUs have the highest Tg among the samples of their series, which confirms the poor phase separation. Usually, the glass transition of hard segments of polyurethanes from aromatic diisocyanates is characterized by higher temperatures (from 60 to 200 °C) [35,36] depending on the content of the hard segment, and with a decrease in the hard segment content, a decrease in the glass transition temperature Tg_hs_ is observed. Thus, the underestimated Tg_hs_ values for all synthesized PUs can be explained by the low content of the hard segment.

For all polyurethanes, characteristic peaks are observed on the first heating curve (Figure 6, solid line), which can be attributed to the rDA reaction for classic DA adduct. However, only for the PU-T series, two pronounced endothermic effects are observed on the first heating curve (Table 4, Figure S5d–f), characteristic of endo- and exo-isomers [37,38]. For classic and modified samples synthesized from furfurylamine and furfuryl alcohol derivatives, two endothermic effects of endo- and exo-isomers (dashed line) are observed on the second heating curve. This behavior can be explained by incompletion of the reverse Diels–Alder reaction (rDA reaction) during the measurement; cooling curves did not show the DA reaction. Thus, the temperature of the rDA reaction (T_rDA_) for all PU samples was in the range from 106 to 155 °C. The data are presented in Table 4. The sensitivity of the DSC method significantly exceeds the sensitivity of the NMR method, which explains the endothermic peaks on the first DSC heating curves (PU-H1, PU-M1, PU-T1, PU-DA-(NFu_2_)_2_), corresponding to trace amounts of classic adducts of the DA reaction in polymer samples. During the first heating–cooling cycle, classic adducts undergo thermal decomposition, then tandem adducts of the DA reaction are formed. According to the literature data [30,31], the reverse DA reaction for tandem adducts occurs at higher temperatures, which was confirmed by additional DSC experiments (Figure S5m–p). During the first heating cycle from 20 to 180 °C, an endothermic peak corresponding to classic adducts was recorded. During the second heating cycle from 20 to 300 °C, only the peak corresponding to the reverse Diels–Alder reaction for tandem adducts was observed in the temperature range 220–230 °C (Table 4).

**Figure 7:**
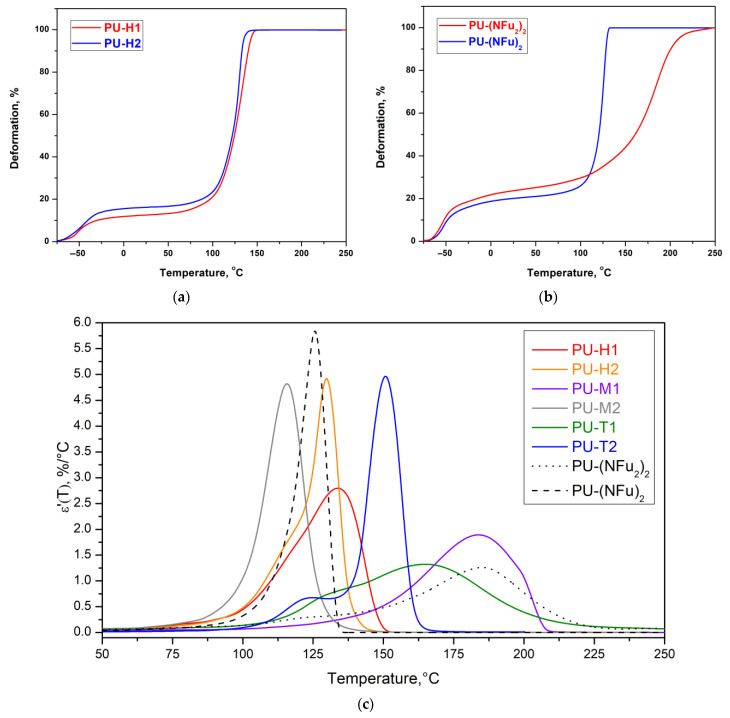
Thermomechanical curves for PU-DA samples: (**a**) modified (PU-H-series); (**b**) traditional; (**c**) curves of the first derivative ε’(T) of PU-DA samples.

The deformation heat resistance of self-healing polyurethanes was determined by thermomechanical analysis (TMA). Tests were performed for all traditional and modified PUs, except samples obtained from furfuryl alcohol derivatives. Figure 7a,b show the TM curves of the PU-H series and PU-DA traditional samples; all other curves are given in the Supplementary Materials (Figure S6a). The curves for all samples have a similar appearance; two visible bends and a plateau of high elasticity are clearly visible [39,40]. The first bend corresponds to the glass transition temperature of the soft segment (Tg_ss_), and the second one corresponds to the rDA reaction and dissociation of hydrogen bonds in the hard segments (T_rDA_H_) [40]. Thus, traditional samples had similar soft segment glass transition temperatures, −54 °C (PU-(NFu_2_)_2_) and −53 °C (PU-(NFu)_2_), which is consistent with the DSC data. However, the temperatures of the second bend differ and are 185 °C for PU-(NFu_2_)_2_ and 126 °C for the PU-(NFu)_2_ sample. For the modified samples, similar effects were observed in the range from −62 to 6 °C and from 116 to 184 °C. The difference in the values of the second bend temperatures, as well as the width of this peak on the first derivative curve, reflects the number and type of DA adducts and the degree of physical network consisting of hydrogen bonds [39,40]. Thus, traditional and modified samples synthesized from difurfurylamine derivatives had higher values of the second transition temperatures (184 °C for PU-M1, 165 °C for PU-T1, and 185 °C for (PU-(NFu_2_)_2_). This behavior is explained by the presence of predominantly tandem DA adducts in their structure, which undergo dissociation at higher temperatures. These samples also had a broader second transition peak on the first-order derivative curve compared to samples based on furfurylamine derivatives (Figure 7c). These results indicate the occurrence of several sequential processes; first, there is dissociation of a small residual amount of classic DA adducts in the polymer structure, after which the destruction of hydrogen bonds and dissociation of tandem DA adducts occur [39,40,41]. In the case of traditional and modified PU-DA from furfurylamine derivatives, the width of the second transition peak was approximately the same, indicating the same amount of classic DA adducts and H-bonds in their structure [40,41]. PU-M2 and PU-T2 samples demonstrated a slightly different thermomechanical behavior; for the PU-M2 sample, the first inflection temperature (Tg_ss_) was the highest in the series (6 °C), and the temperature of the second transition peak (T_rDA_H_) was the lowest in the series and amounted to 116 °C. This fact is associated with the predominance of the mixed phase in the structure of the PU-M2 sample, accompanied by a decrease in the segmental mobility of the soft segments, which, in turn, is probably associated with the presence of a dense physical network between the hard and soft segments and a wide molecular weight distribution [26,42]. The PU-T2 sample, on the contrary, demonstrated the lowest value of the first bend, which indicates a more distinct phase segregation in its structure [42,43]. A slight shift of the second bend to the higher temperature region may be due to the peculiarities of the research method.

The height of the high-elasticity area also indicates the density of the physical bond network—the denser the network, the lower the deformation value in this area [39]. In the case of traditional PU samples, the degree of deformation in the high-elasticity zone was 25 and 20% for PU-(NFu_2_)_2_, and PU-(NFu)_2_, respectively. Similar values for the modified samples from difurfurylamine derivatives remained constant and amounted to 13% (PU-H1), 15% (PU-M1), and 15% (PU-T1), and for the polymers from furfurylamine derivatives 15% (PU-H2), 10% (PU-M2), and 20% (PU-T2) (Figure S6a). Thus, the obtained deformability data in the high-elasticity zone indicate the formation of a greater number of intermolecular hydrogen bonds in the structure of modified PUs synthesized from difurfurylamine derivatives. Furfurylamine-derived samples PU-H2, PU-T2, and PU-(NFu)_2_ have a similar density of the physical bond network. However, as in the case of the second transition peak, the properties of the PU-M2 were noticeably different. This sample had the lowest degree of deformation in the high-elasticity zone in the series, which can also be caused by strong intermolecular interaction between the soft and hard segments, preventing free movement of the macromolecules of the soft phase [43]. The measurement results are presented in Table 5.

### 3.3. Mechanical and Self-Healing Properties

**Figure 8:**
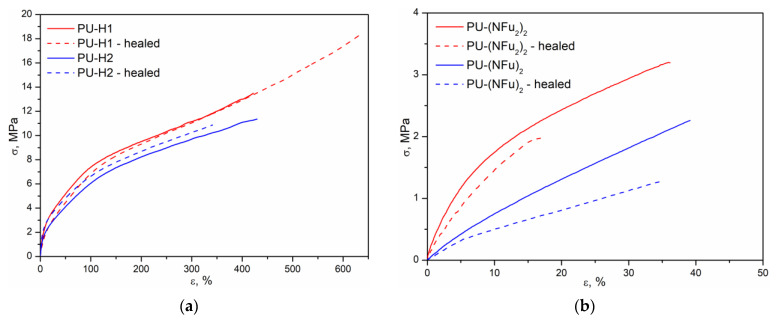
Load diagrams for PU-DA: (**a**) original and healed PU-H (PU-H-series); (**b**) original and healed PU-DA traditional.

In previous works [15,16,18], traditional PU-DA samples have low self-healing efficiency due to the concentration of furan groups in the hard domains. We assumed that the use of a modified approach consisting of using a maleimide-terminated preform and furan-containing agents of different structures would result in an increase in the availability of furan and maleimide groups for the DA reaction. This method is aimed at increasing the self-healing efficiency, as well as strengthening the resulting material due to a higher content of the hard segment. This segment acts as a physical reinforcement for PU, forming hydrogen bonds between the hard segments of the prepolymer and the urea fragments of the furan-containing agents. The efficiency of the self-healing process was quantified by measuring the elastic modulus and uniaxial tensile strength. All samples except the furfuryl alcohol-derived polyurethanes were examined (FOH polymer films were sticky and unsuitable for testing). To determine the elastic modulus in each group of samples, the average load curve was used, i.e., the average curve for all samples in the group. Representative load diagrams for undamaged and healed PU-H series and PU-DA traditional samples are shown in Figure 8; the loading diagrams for the remaining samples had a similar appearance. During the self-healing procedure on the first heating to 120 °C, thermal destruction of the Diels–Alder reaction adducts and hydrogen bonds occurs. For phase-separated systems, exceeding the glass transition temperatures increases the mobility of polymer chains in the material. Both of these factors make possible the diffusion of the material into the damaged area, leading to the replacement of the lost volume. The subsequent holding at 60 °C leads to the final restoration of the structure, and the formation of new covalent and hydrogen bonds, and also the formation of a phase-separated system. For polymeric materials with classic adducts, all the above-mentioned processes are possible. However, for polymeric materials with tandem adducts, the self-healing mechanism via covalent reversible interactions is “switched off”. The reason for this phenomenon is the broader temperature range of the reverse Diels–Alder reaction for tandem adducts than the cycling mode of classic adducts (Figure S5m–p).

Thus, the DSC and TMA data indicate microphase separation processes in the structure of traditional and modified PU-DA. And during the tensile test, the soft and hard segments are above their respective Tg values. The loading curves of the initial and cured samples showed the behavior of a weakly crosslinked elastomer, elongating and breaking at relatively low stress (Figure 8). The mechanical property values for the initial PU-DA traditional samples were for PU-(NFu_2_)_2_: E_o_ = 40 ± 4 MPa; σ_o_ = 3.2 ± 0.3 MPa, and for PU-(NFu)_2_: E_o_ = 14 ± 4 MPa; σ_o_ = 2.4 ± 0.3 MPa. The obtained PU-DA-modified demonstrated higher mechanical properties compared to PU-DA-traditional due to the content of additional hydrogen bonds, which limit the mobility of polymer chains during stretching [44]. Thus, the values of the elastic modulus for the original modified samples were in the range from 23 to 110 MPa, and the values of the tensile strength were from 8 to 20 MPa (Table 6).

Examination of mechanical properties of PU samples before and after thermal healing showed that the efficiency of Young’s modulus (η_E_(%)) and tensile strength (η_σ_(%)) restoration for traditional samples was 68% and 67% for the PU-(NFu_2_)_2_ sample, and 50% and 50% for PU-(NFu)_2_, respectively. These results indicate the formation of fewer classic DA adducts and hydrogen bonds in PU-(NFu)_2_ and hydrogen bonds in PU-(NFu_2_)_2_ after the healing process. In the case of the modified polymers from difurfurylamine derivatives (PU-H1, PU-M1, and PU-T1), the efficiency of Young’s modulus (η_E_(%)) and tensile strength (η_σ_(%)) restoration ranged from 44 to 76%, and from 60 to 129%, respectively. Low self-healing efficiency was observed for samples PU-M1 (η_E_ = 44%, η_σ_ = 60%) and PU-M2 (η_E_ = 74%, η_σ_ = 64%). For PU-M1, this fact is probably caused by the presence of predominantly only tandem DA adducts, which is confirmed by DSC analysis. Therefore, in the resulting material, a contribution of reversible covalent interactions to the healing process is absent. This fact led to the low property restoration efficiency, and self-healing occurs mainly due to hydrogen bonds [45]. The mixed phase predominates in the structure of the PU-M2 sample. This phase is formed via the hydrogen bonds between the hard and soft segments and characterized by lower strength compared to bonds of the “hard-hard” segment type [46,47], which in turn determines the lowest initial value of Young’s modulus (23 MPa) among the PU-DA-modified samples. Their sufficiently large number hampered the movement of macromolecules relative to each other and the orientation of the soft segments along the axis of the applied load, which prevented plastic deformation and determined the sufficiently high tensile strength of the material [48,49]. In the case of PU-H1 (η_E_ = 64%, η_σ_ = 129%) and PU-T1 (η_E_ = 76%, η_σ_ = 113%) samples, slight strengthening was observed. According to the DSC results, a certain amount of classic DA adducts is present in the structure of these samples. Thus, the thermal cycling procedure leads to a complete conversion of classic adducts into tandem ones, which can explain the increase in the strength of the material after the healing cycle and the self-healing efficiency values over 100%. PU-H2 (η_E_ = 100%, η_σ_ = 81%) and PU-T2 (η_E_ = 93%, η_σ_ = 90%) showed approximately the same efficiency of Young’s modulus restoration, due to the formation of the same amount of DA adducts and H-bonds after healing [29]. PU-T1 and PU-T2 samples, among all modified polymers, showed the most optimal combination of the elastic modulus and tensile strength recovery in the series. This could be explained by the high affinity of furan-containing agents of the T-series to the hard block of the obtained PU, which does not lead to a sharp decrease in the mobility of macromolecules, and, as a result, to achieving high values of self-healing efficiency.

**Figure 9:**
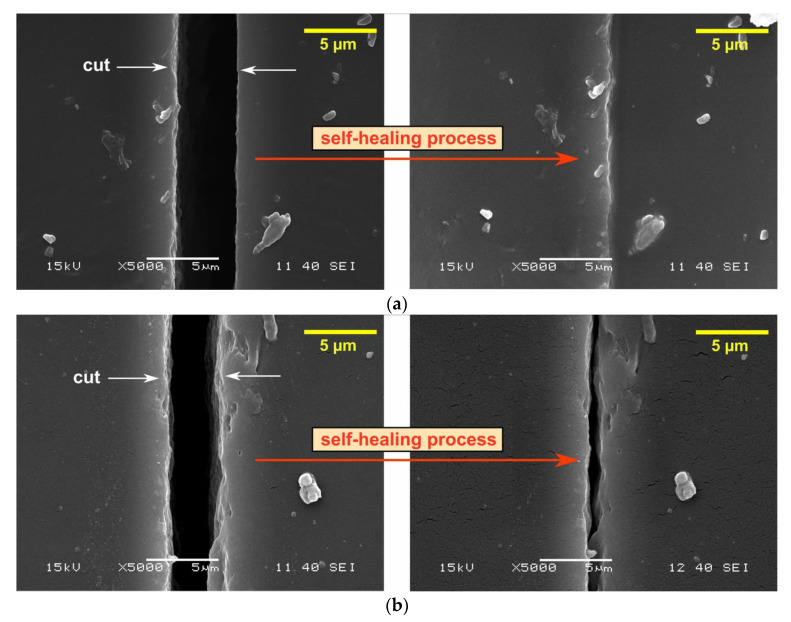
SEM-images of PUs: (**a**) modified (PU-T2); (**b**) traditional (PU-(NFu)_2_).

Visual investigation of the self-healing ability was performed by the method of scanning electron microscopy (SEM). The polymer sample was cut with a surgical scalpel and then subjected to a two-stage healing cycle (1.5 h at 120 °C, 24 h at 60 °C). All polyurethane samples showed self-healing ability. However, SEM analysis revealed that modified PU-DA exhibited markedly superior defect closure compared to traditional PU (Figure 9).

## 4. Conclusions

A series of new modified polyurethanes with a self-healing effect were obtained from furan–urethane monomers. The properties of modified PUs were compared with the properties of PUs obtained by a traditional method widely described in the literature (from a furan-terminated oligomer and low-molecular-weight bismaleimide). The GPC method showed that the modified PUs had a higher molecular weight and a narrower polydispersity compared to traditional samples. On the results of the uniaxial tension test, all samples, except PU from furfuryl alcohol derivatives, showed self-healing ability. The proposed modified approach increased the self-healing efficiency to 80–100% and strengthened the obtained materials several times. Visual investigation of self-healing properties by SEM on one of the modified samples showed the successful completion of the healing process.

In addition, new polyurethane materials with tandem Diels–Alder adducts were obtained. The introduction of tandem adducts of the DA reaction makes such materials suitable for processing and further reuse of their components. Combining several self-healing mechanisms in such materials allows for obtaining polymers with high efficiency of mechanical property restoration.

## Figures and Tables

**Table 1:** Modified and traditional PUs: nomenclature and corresponding formulations.

PU System	Prepolymer	Curing Agent
PU-H1	HEMI-TDI-PPG	DFA-H
PU-H2		FA-H
PU-H3		FOH-H
PU-M1		DFA-M
PU-M2		FA-M
PU-M3		FOH-M
PU-T1		DFA-T
PU-T2		FA-T
PU-T3		FOH-T
PU-(NFu_2_)_2_	DFA-TDI-PPG	BMI
PU-(NFu)_2_	FA-TDI-PPG	
PU-(OFu)_2_	FOH-TDI-PPG	

**Table 2:** Molecular weight characteristics of traditional and modified PU samples.

Sample	M_n_ (Da)	M_w_ (Da)	M_w_/M_n_
PU-H1	3100	10,300	3
PU-H2	3000	9900	3
PU-H3	3700	11,300	3
PU-M1	4700	27,000	6
PU-M2	13,500	93,300	7
PU-M3	4800	26,000	6
PU-T1	3100	9900	3
PU-T2	2800	9800	4
PU-T3	5600	38,400	7
PU-(NFu_2_)_2_	2700	28,000	10
PU-(NFu)_2_	6200	112,400	18
PU-(OFu)_2_	2700	23,000	9

**Table 3:** The results of thermogravimetric analysis of PUs.

Sample	T_5%_, °C	T_10%_, °C	T_max_, °C	Char Residue, %
PU-H1	278	304	371	9
PU-H2	239	283	373	4
PU-H3	236	278	377	15
PU-M1	268	287	383	10
PU-M2	241	253	269	23
PU-M3	270	301	382	17
PU-T1	256	284	376	9
PU-T2	233	266	378	10
PU-T3	207	215	226	26
PU-(NFu_2_)_2_	275	297	380	17
PU-(NFu)_2_	247	286	386	17
PU-(OFu)_2_	278	314	387	18

**Table 4:** The results of differential scanning calorimetry of PUs.

Sample	Tg_ss_, °C	Tg_hs_,°C	T_rDA(classic)_,°C	T_rDA(tandem)_,°C
PU-H1	−48	−2	114	230
PU-H2	−50	−4	137	-
PU-H3	−44	−4	139	-
PU-M1	−50	7	116	220
PU-M2	−44	7	142	-
PU-M3	−38	4	139	-
PU-T1	−50	7	106, 155	226
PU-T2	−47	7	114, 141	-
PU-T3	−40	6	110, 143	-
PU-(NFu_2_)_2_	−55	−4	137	226
PU-(NFu)_2_	−54	−7	136	-
PU-(OFu)_2_	−51	-	113	-

**Table 5:** TMA results for PU-DA samples.

Sample	Tg_ss_, °C	T_rDA_H_, °C
PU-H1	−51	134
PU-H2	−44	130
PU-M1	−50	184
PU-M2	6	116
PU-T1	−62	165
PU-T2	−50	151
PU-(NFu_2_)_2_	−54	185
PU-(NFu)_2_	−53	126
PU-H1	−51	134

**Table 6:** Tensile test results of PU-DA samples.

Samples	E_o_ (MPa)	E_h_ (MPa)	η_E_ (%)	σ_o_ (MPa)	σ_h_ (MPa)	η_σ_ (%)
PU-H1	42 ± 4	27 ± 4	64	14 ± 1	18 ± 2	129
PU-H2	54 ± 4	54 ± 12	100	11 ±1	9 ± 2	81
PU-M1	95 ± 7	42 ± 4	44	20 ± 3	12 ± 3	60
PU-M2	23 ± 2	17 ± 3	74	11 ± 1	7 ± 2	64
PU-T1	110 ± 5	84 ± 17	76	8 ± 1	9 ± 1	113
PU-T2	55 ± 3	51 ± 8	93	10 ± 1	9 ± 1	90
PU-(NFu_2_)_2_	40 ± 4	27 ± 7	68	3.2 ± 0.3	2.1 ± 0.3	67
PU-(NFu)_2_	14 ± 4	7 ± 1	50	2.4 ± 0.3	1.2 ± 0.1	50

## Data Availability

The original contributions presented in this study are included in the article/Supplementary Materials. Further inquiries can be directed to the corresponding author.

## Supplementary Materials

The following supporting information can be downloaded at: https://www.mdpi.com/article/10.3390/polym17141951/s1, Figure S1a: HR-MS spectra of DFA-H; Figure S1b: HR-MS spectra of FA-H; Figure S1c: HR-MS spectra of FOH-H; Figure S1d: HR-MS spectra of FA-T; Figure S1e: HR-MS spectra of FOH-T; Figure S1f: HR-MS spectra of DFA-M; Figure S1g: HR-MS spectra of FA-M; Figure S1h: HR-MS spectra of FOH-M; Figure S2a: ^1^H NMR spectrum of the PU prepolymer in DMSO-d_6_; Figure S2b: ^13^C NMR spectrum of the PU prepolymer in DMSO-d_6_; Figure S2c: ^1^H NMR spectrum of the PU-H1 in DMSO-d_6_; Figure S2d: ^13^C NMR spectrum of the PU-H1 in DMSO-d_6_; Figure S2e: ^1^H NMR spectrum of the PU-H2 in DMSO-d_6_; Figure S2f: ^13^C NMR spectrum of the PU-H2 in DMSO-d_6_; Figure S2g: ^1^H NMR spectrum of the PU-H3 in DMSO-d_6_; Figure S2h: ^13^C NMR spectrum of the PU-H3 in DMSO-d_6_; Figure S2i: ^1^H NMR spectrum of the PU-T1 in DMSO-d_6_; Figure S2j: ^13^C NMR spectrum of the PU-T1 in DMSO-d_6_; Figure S2k: ^1^H NMR spectrum of the PU-T2 in DMSO-d_6_; Figure S2l: ^13^C NMR spectrum of the PU-T2 in DMSO-d_6_; Figure S2m: ^1^H NMR spectrum of the PU-T3 in DMSO-d_6_; Figure S2n: ^13^C NMR spectrum of the PU-T3 in DMSO-d_6_; Figure S2o: ^1^H NMR spectrum of the PU-M1 in DMSO-d_6_; Figure S2p ^13^C NMR spectrum of the PU-M1 in DMSO-d_6_; Figure S2q: ^1^H NMR spectrum of the PU-M2 in DMSO-d_6_; Figure S2r: ^13^C NMR spectrum of the PU-M2 in DMSO-d_6_; Figure S2s: ^1^H NMR spectrum of the PU-M3 in DMSO-d_6_; Figure S2t: ^13^C NMR spectrum of the PU-M3 in DMSO-d_6_; Figure S2u: Kinetic data for the polymerization reaction of PU-H1; Figure S3a: ATI-FTIR spectrum of the PU-H1; Figure S3b: ATI-FTIR spectrum of the PU-H2; Figure S3c: ATI-FTIR spectrum of the PU-H3; Figure S3d: ATI-FTIR spectrum of the PU-T1; Figure S3e: ATI-FTIR spectrum of the PU-T2; Figure S3f: ATI-FTIR spectrum of the PU-T3; Figure S3g: ATI-FTIR spectrum of the PU-M1; Figure S3h: ATI-FTIR spectrum of the PU-M2; Figure S3i: ATI-FTIR spectrum of the PU-M3; Figure S3j: ATI-FTIR spectrum of the PU-(NFu_2_)_2_; Figure S3k: ATI-FTIR spectrum of the PU-(NFu)_2_; Figure S3l: ATI-FTIR spectrum of the PU-(OFu)_2_; Figure S4a: TGA and DTG curves of the PU-H1; Figure S4b: TGA and DTG curves of the PU-H2; Figure S4c: TGA and DTG curves of the PU-H3; Figure S4d: TGA and DTG curves of the PU-T1; Figure S4e: TGA and DTG curves of the PU-T2; Figure S4f: TGA and DTG curves of the PU-T3; Figure S4g: TGA and DTG curves of the PU-M1; Figure S4h: TGA and DTG curves of the PU-M2; Figure S4i: TGA and DTG curves of the PU-M3; Figure S4j: TGA and DTG curves of the PU-(NFu_2_)_2_; Figure S4k: TGA and DTG curves of the PU-(NFu)_2_; Figure S4l: TGA and DTG curves of the PU-(OFu)_2_; Figure S5a: DSC curve of the PU-H1; Figure S5b: DSC curve of the PU-H2; Figure S5c: DSC curve of the PU-H3; Figure S5d: DSC curve of the PU-T1; Figure S5e: DSC curve of the PU-T2; Figure S5f: DSC curve of the PU-T3; Figure S5g: DSC curve of the PU-M1; Figure S5h: DSC curve of the PU-M2; Figure S5i: DSC curve of the PU-M3; Figure S5j: DSC curve of the PU-(NFu_2_)_2_; Figure S5k: DSC curve of the PU-(NFu)_2_; Figure S5l: DSC curve of the PU-(OFu)_2_; Figure S5m: Additional DSC curve of the PU-H1; Figure S5n: Additional DSC curve of the PU-T1; Figure S5o: Additional DSC curve of the PU-M1; Figure S5p: Additional DSC curve of the PU-(NFu_2_)_2_; Figure S6a: TM curves of all polyurethanes.
