# Supplementary material for: Furan–Urethane Monomers for Self-Healing Polyurethanes
Source: Polymers (Basel). 2025 Jul 16;17(14):1951. doi: 10.3390/polym17141951 (PMC12298714; doi:10.3390/polym17141951)
Supplement: Supplementary file 1 [file polymers-17-01951-s001.zip › polymers-3730852-supplementary.pdf]

SUPPORTING INFORMATION

Furan-urethane monomers for self-healing polyurethanes

Polina Ponomareva [1,*], Zalina Lokiaeva [1], Daria Zakharova [2], Ilya Tretyakov [1], Elena Platonova [1], Aleksey Shapagin [3], Olga Alexeeva [4], Evgenia Antoshkina [5], Vitaliy Solodilov [1], Gleb Yurkov[1] and Alexandr Berlin[1]

[1] Semenov Institute of Chemical Physics, Russian Academy of Sciences, 119991 Moscow, Russia

[2] NTI Center "Digital Materials Science: New Materials and substances" Bauman Moscow State Technical University, 2nd Baumanskaya Street 5, 105005 Moscow, Russia.

[3] Frumkin Institute of Physical Chemistry and Electrochemistry, Leninsky Prospect 31, Building 4, 119071 Moscow, Russia.

[4] N.M. Emanuel Institute of Biochemical Physics, Russian Academy of Sciences, 119334 Moscow, Russia, Kosygina Street, Building 4.

[5] A.N. Nesmeyanov Institute of Organoelement Compounds of Russian Academy of Sciences, Vavilova Street 28, Building 1, 119334 Moscow, Russia.

* Correspondence: ponomareva.p.f@gmail.com (P.P.)

Table of the Contents

Experimental Section
Characterization
Figure S1a. HR-MS spectra of DFA-H
Figure S1b. HR-MS spectra of FA-H
Figure S1c. HR-MS spectra of FOH-H
Figure S1d. HR-MS spectra of FA-T
Figure S1e. HR-MS spectra of FOH-T
Figure S1f. HR-MS spectra of DFA-M
Figure S1g. HR-MS spectra of FA-M
Figure S1h. HR-MS spectra of FOH-M
2. Nuclear Magnetic Resonance (NMR)
Figure S2a. ^{1}H NMR spectrum of the PU prepolymer in DMSO-d_6.
Figure S2b. ^{13}C NMR spectrum of the PU prepolymer in DMSO-d_6.
Figure S2c. ^{1}H NMR spectrum of the PU-H1 in DMSO-d_6.
Figure S2d. ^{13}C NMR spectrum of the PU-H1 in DMSO-d_6.
Figure S2e. ^{1}H NMR spectrum of the PU-H2 in DMSO-d_6.
Figure S2f. ^{13}C NMR spectrum of the PU-H2 in DMSO-d_6.
Figure S2g. ^{1}H NMR spectrum of the PU-H3 in DMSO-d_6.
Figure S2h. ^{13}C NMR spectrum of the PU-H3 in DMSO-d_6.
Figure S2i. ^{1}H NMR spectrum of the PU-T1 in DMSO-d_6.
Figure S2j. ^{13}C NMR spectrum of the PU-T1 in DMSO-d_6.
Figure S2k. ^{1}H NMR spectrum of the PU-T2 in DMSO-d_6.
Figure S2l. ^{13}C NMR spectrum of the PU-T2 in DMSO-d_6.
Figure S2m. ^{1}H NMR spectrum of the PU-T3 in DMSO-d_6.
Figure S2n. ^{13}C NMR spectrum of the PU-T3 in DMSO-d_6.
Figure S2o. ^{1}H NMR spectrum of the PU-M1 in DMSO-d_6.
Figure S2p. ^{13}C NMR spectrum of the PU-M1 in DMSO-d_6.
Figure S2q. ^{1}H NMR spectrum of the PU-M2 in DMSO-d_6.
Figure S2r. ^{13}C NMR spectrum of the PU-M2 in DMSO-d_6.
Figure S2s. ^{1}H NMR spectrum of the PU-M3 in DMSO-d_6.
Figure S2t. ^{13}C NMR spectrum of the PU-M3 in DMSO-d_6.
Figure S2u. Kinetic data for the polymerization reaction of PU-H1.
3. Attenuated Total Reflection-Fourier Transform Infrared Spectroscopy (ATR-FTIR)
Figure S3a. ATI-FTIR spectrum of the PU-H1.
Figure S3b. ATI-FTIR spectrum of the PU-H2.
Figure S3c. ATI-FTIR spectrum of the PU-H3.
Figure S3d. ATI-FTIR spectrum of the PU-T1.
Figure S3e. ATI-FTIR spectrum of the PU-T2.
Figure S3f. ATI-FTIR spectrum of the PU-T3.
Figure S3g. ATI-FTIR spectrum of the PU-M1.
Figure S3h. ATI-FTIR spectrum of the PU-M2.
Figure S3i. ATI-FTIR spectrum of the PU-M3.
Figure S3j. ATI-FTIR spectrum of the PU-$(NFu_2)_2$.
Figure S3k. ATI-FTIR spectrum of the PU-$(NFu)_2$.
Figure S3l. ATI-FTIR spectrum of the PU-$(OFu)_2$.
4. Thermogravimetric Analysis (TGA)
Figure S4a. TGA and DTG curves of the PU-H1.

Figure S4b. TGA and DTG curves of the PU-H2.
Figure S4c. TGA and DTG curves of the PU-H3.
Figure S4d. TGA and DTG curves of the PU-T1.
Figure S4e. TGA and DTG curves of the PU-T2.
Figure S4f. TGA and DTG curves of the PU-T3.
Figure S4g. TGA and DTG curves of the PU-M1.
Figure S4h. TGA and DTG curves of the PU-M2.
Figure S4i. TGA and DTG curves of the PU-M3.
Figure S4j. TGA and DTG curves of the PU-$(NFu_2)_2$.
Figure S4k. TGA and DTG curves of the PU-$(NFu)_2$.
Figure S4l. TGA and DTG curves of the PU-$(OFu)_2$.
5. Differential Scanning Calorimetry (DSC)
Figure S5a. DSC curve of the PU-H1.
Figure S5b. DSC curve of the PU-H2.
Figure S5c. DSC curve of the PU-H3.
Figure S5d. DSC curve of the PU-T1.
Figure S5e. DSC curve of the PU-T2.
Figure S5f. DSC curve of the PU-T3.
Figure S5g. DSC curve of the PU-M1.
Figure S5h. DSC curve of the PU-M2.
Figure S5i. DSC curve of the PU-M3.
Figure S5j. DSC curve of the PU-$(NFu_2)_2$.
Figure S5k. DSC curve of the PU-$(NFu)_2$.
Figure S5l. DSC curve of the PU-$(OFu)_2$.
Figure S5m. Additional DSC curve of the PU-H1.
Figure S5n. Additional DSC curve of the PU-T1.
Figure S5o. Additional DSC curve of the PU-M1.
Figure S5p. Additional DSC curve of the PU-$(NFu_2)_2$.
6. Thermomechanical analysis (TMA)
Figure S6a. TM curves of all polyurethanes

Experimental Section
Characterization

1. High-resolution mass spectra (HR-MS)

Di- (FA-H, FOH-H, FA-T, FOH-T, FA-M, FOH-M) and tetrafurane derivatives (DFA-H, DFA-M) were previously obtained according to the literature method [1] and characterized by the method 1H-, 13C-spectroscopy. Additionally, HRMS experiments were performed to prove the composition of the monomeric compounds used.

<u>DFA-H</u> HRMS (ESI): calculated for $C_{28}H_{34}N_4O_6Cl$ $[M+Cl]^-$ 557.2172, found: 557.2173; calculated for $C_{29}H_{35}N_4O_8$ $[M+HCOO]^-$ 567.2460, found: 567.2461; calculated for $C_{28}H_{34}N_5O_9$ $[M+NO_3]^-$ 584.2362, found: 584.2365; calculated for $C_{30}H_{36}N_4O_{10}Na$ $[M+HCOONa+HCOO]^-$ 635.2335, found: 635.2339.

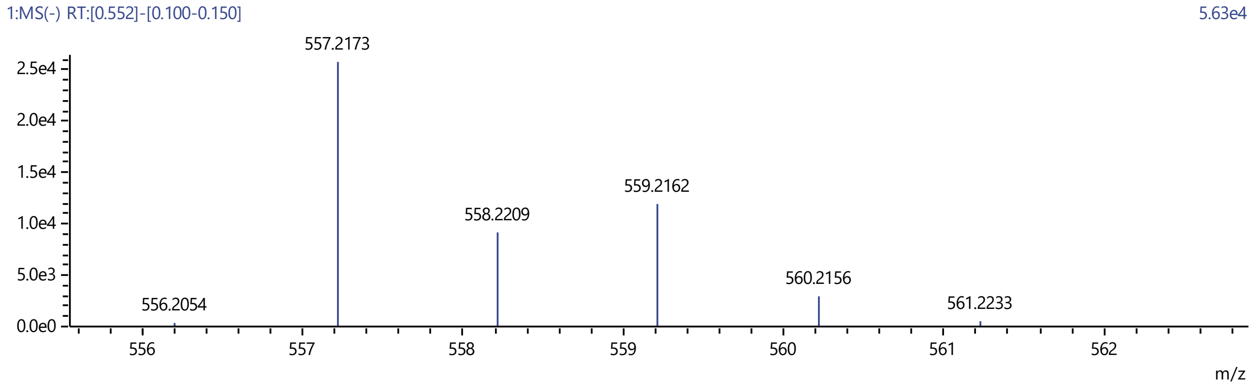

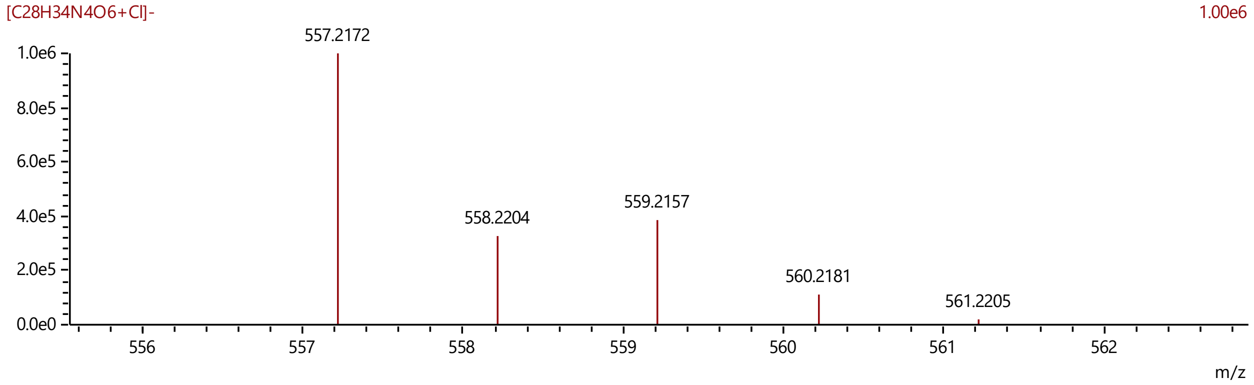

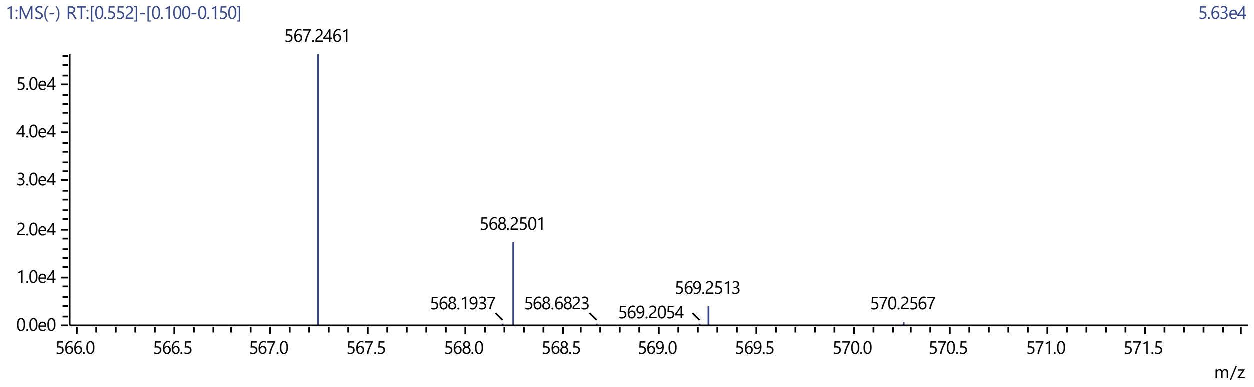

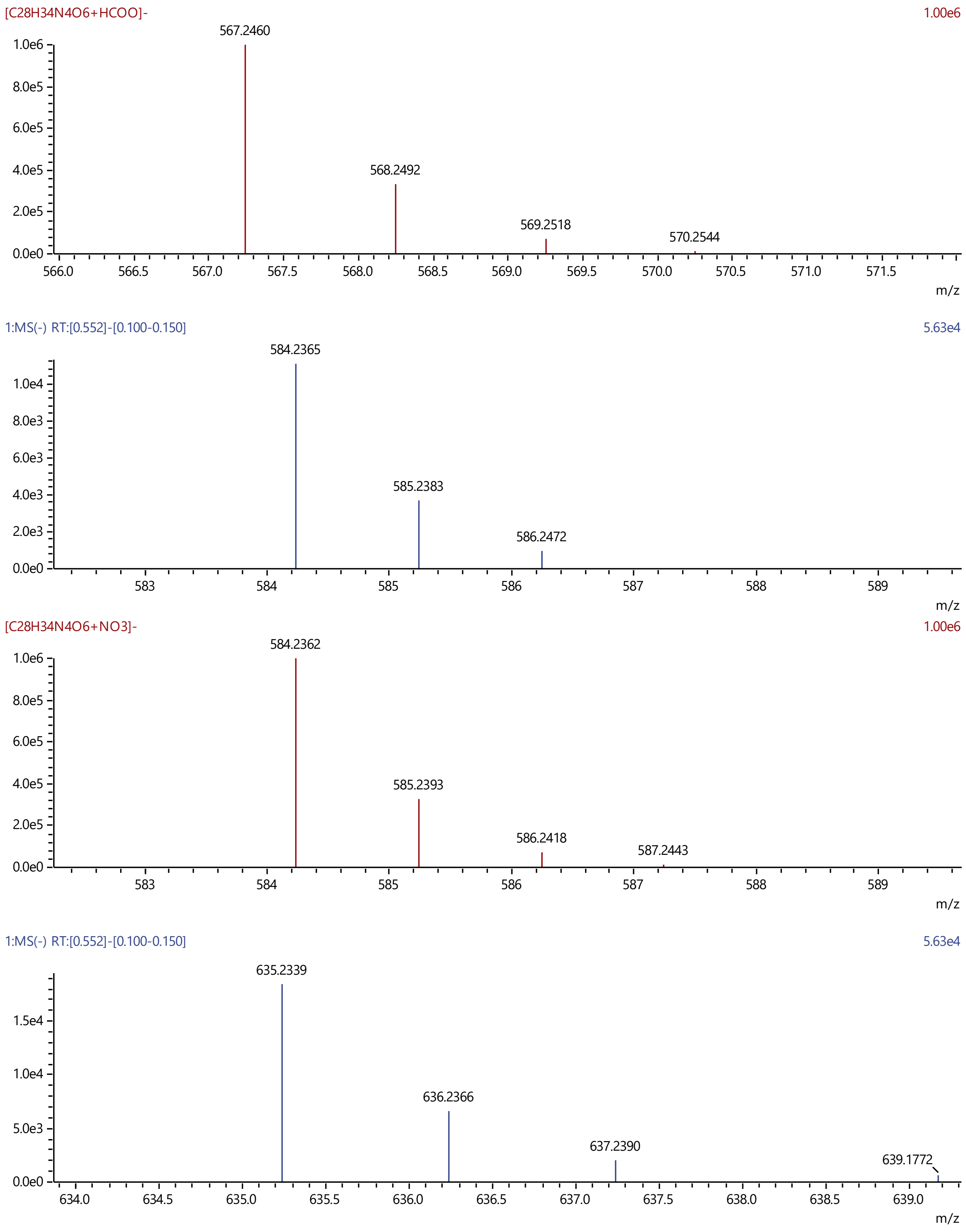

[C28H34N4O6+HCOO]-
1.00e6
567.2460
568.2492
569.2518
570.2544
m/z
1:MS(-) RT:[0.552]-[0.100-0.150]
5.63e4
584.2365
585.2383
586.2472
m/z
[C28H34N4O6+NO3]-
1.00e6
584.2362
585.2393
586.2418
587.2443
m/z
1:MS(-) RT:[0.552]-[0.100-0.150]
5.63e4
635.2339
636.2366
637.2390
639.1772
m/z

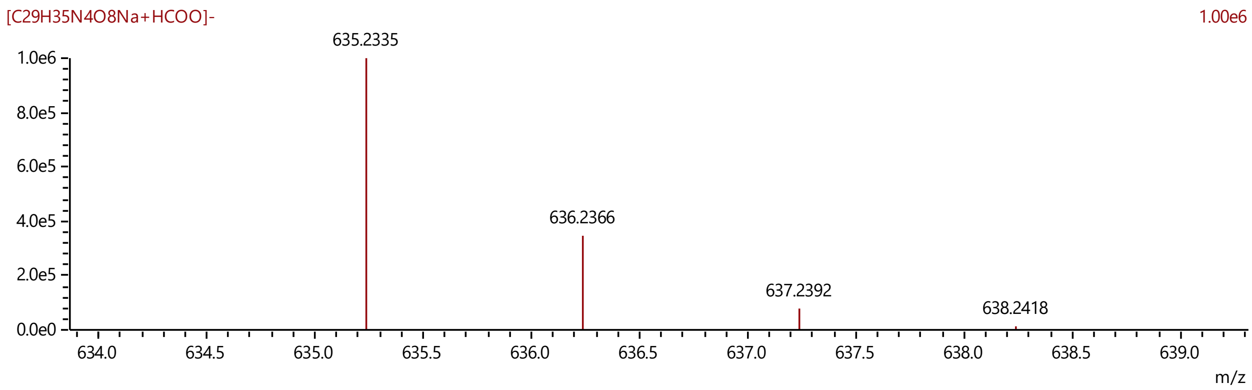

Figure S1a. HR-MS spectra of DFA-H

<u>FA-H</u> HRMS (ESI): calculated for $C_{18}H_{27}N_4O_4$ $[M+H]^+$ 363.2027, found: 363.2031; calculated for $C_{18}H_{27}N_4O_4$ $[M-H]^-$ 361.1881, found: 361.1886; calculated for $C_{18}H_{26}N_4O_4Cl$ $[M+Cl]^-$ 397.1648, found: 397.1648; calculated for $C_{18}H_{26}N_5O_7$ $[M+NO_3]^-$ 424.1838, found: 424.1836.

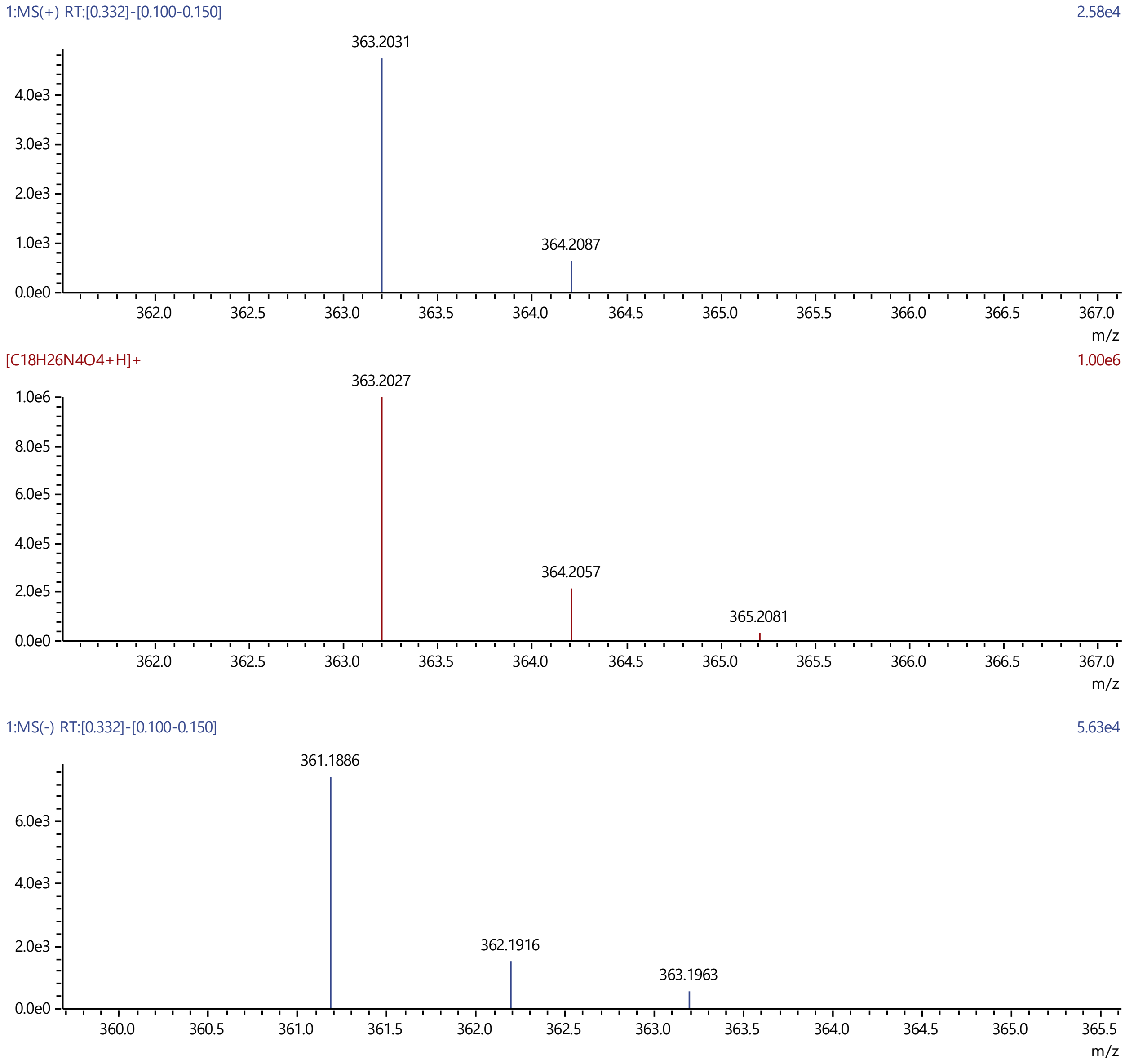

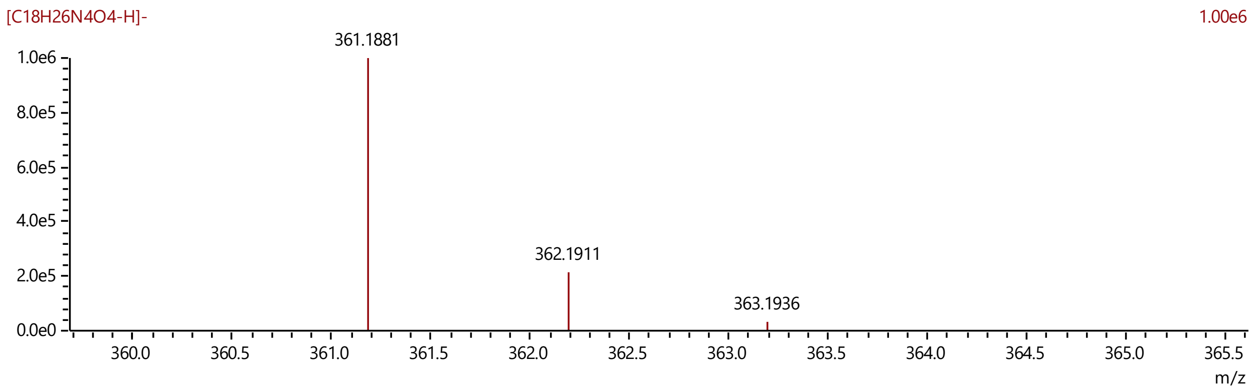
[C18H26N4O4-H]-
1.00e6
361.1881
362.1911
363.1936
m/z

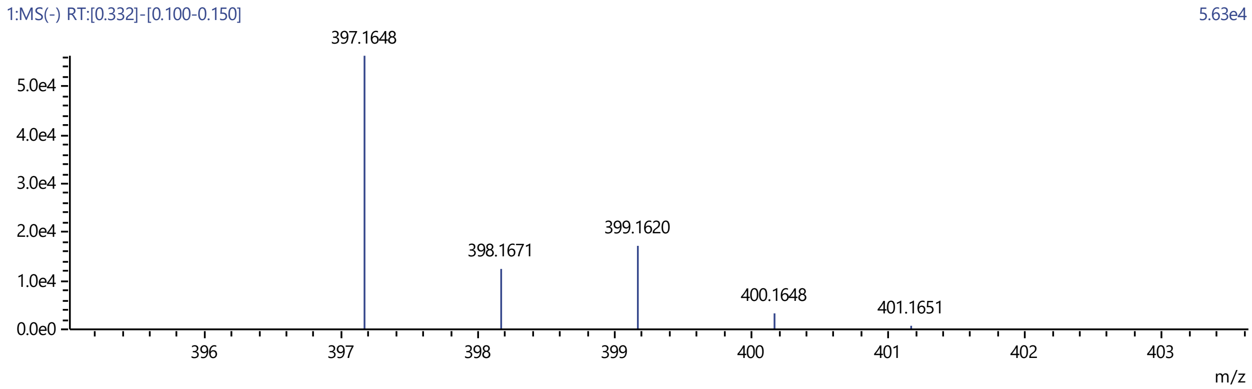
1:MS(-) RT:[0.332]-[0.100-0.150]
5.63e4
397.1648
398.1671
399.1620
400.1648
401.1651
m/z

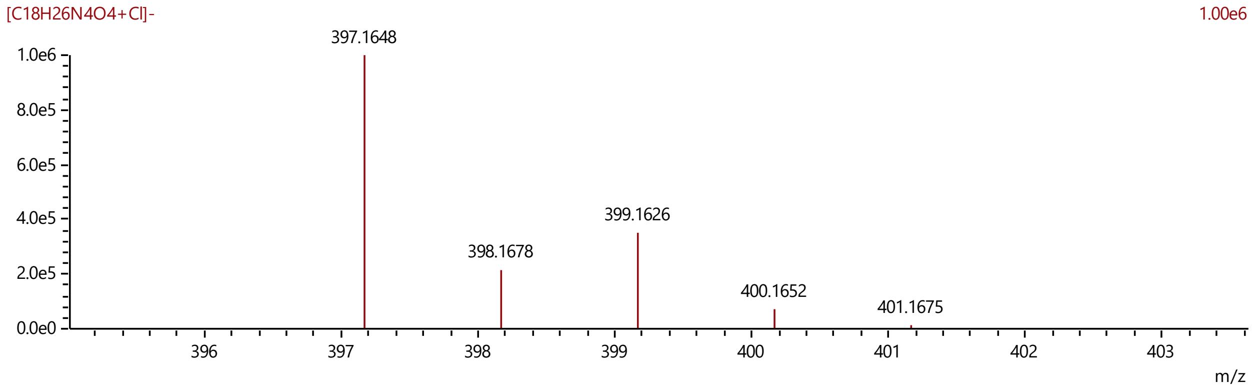
[C18H26N4O4+Cl]-
1.00e6
397.1648
398.1678
399.1626
400.1652
401.1675
m/z

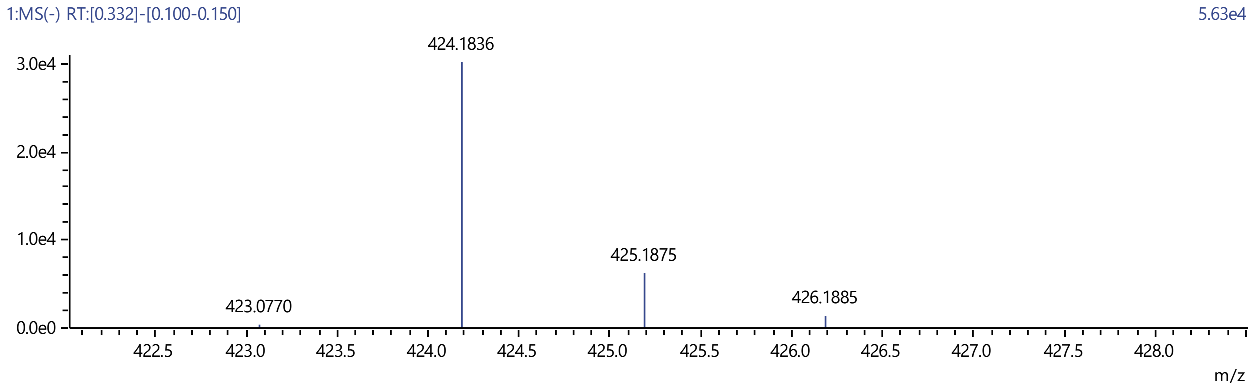
1:MS(-) RT:[0.332]-[0.100-0.150]
5.63e4
424.1836
425.1875
426.1885
423.0770
m/z

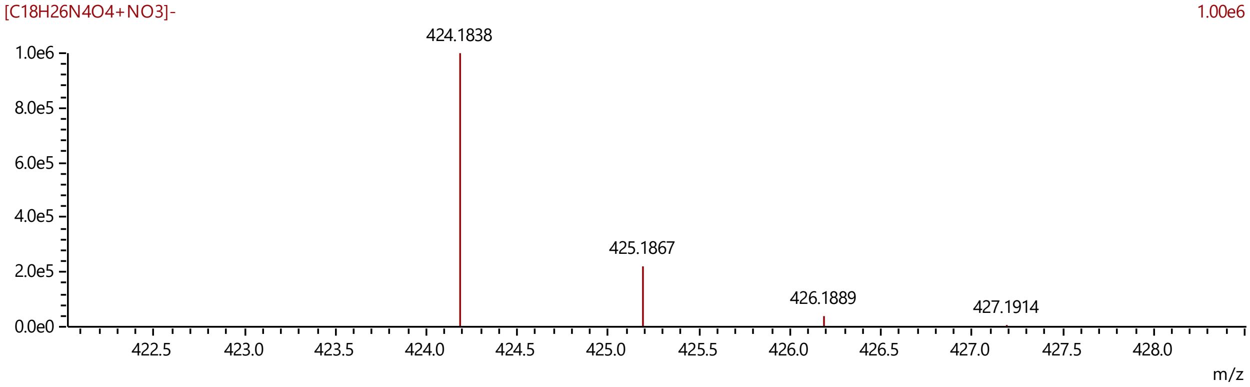

Figure S1b. HR-MS spectra of FA-H

<u>FOH-H</u> HRMS (ESI): calculated for $C_{18}H_{25}N_2O_6$ $[M+H]^+$ 365.1707, found: 365.1711; calculated for $C_{18}H_{24}N_2O_6Na$ $[M+Na]^+$ 387.1527, found: 387.1534; calculated for $C_{36}H_{48}N_4O_{12}Na$ $[2M+Na]^+$ 751.3161., found: 751.3171; calculated for $C_{18}H_{24}N_2O_6Cl$ $[M+Cl]^-$ 399.1328, found: 399.1325;

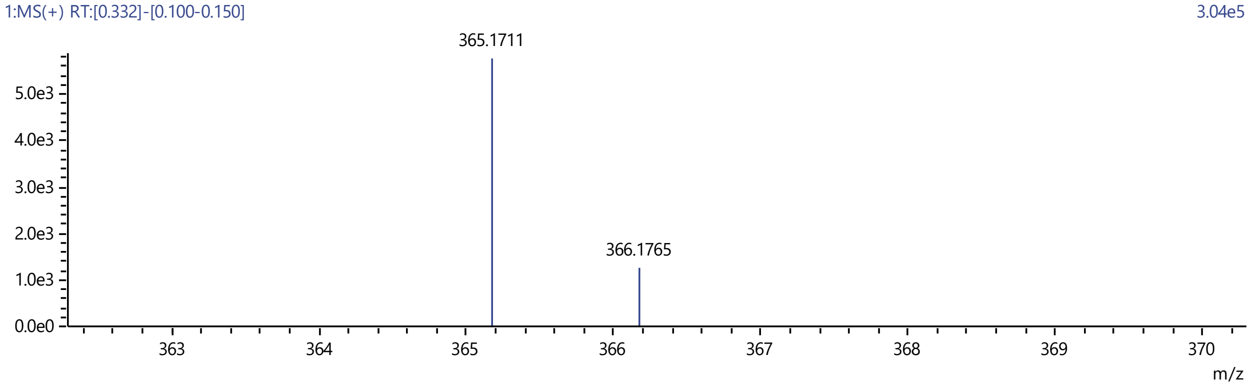

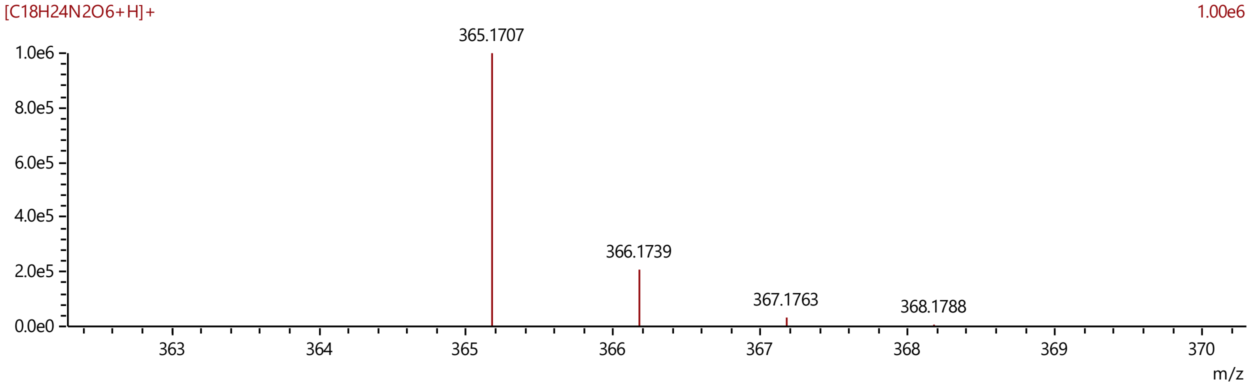

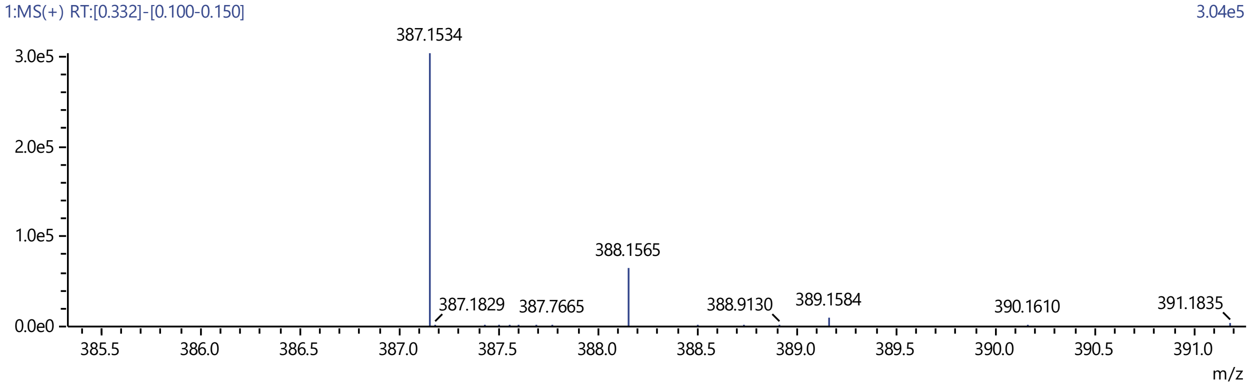

[C18H24N2O6+Na]+ 1.00e6

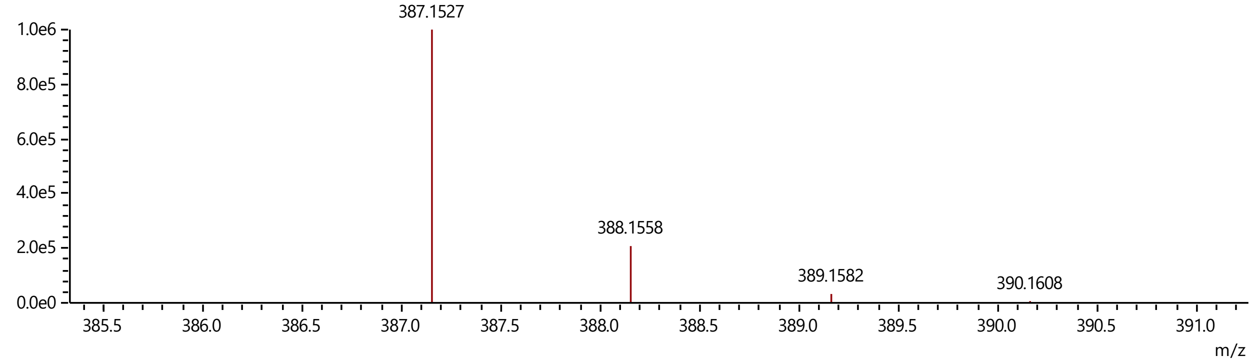

1:MS(+) RT:[0.332]-[0.100-0.150] 3.04e5

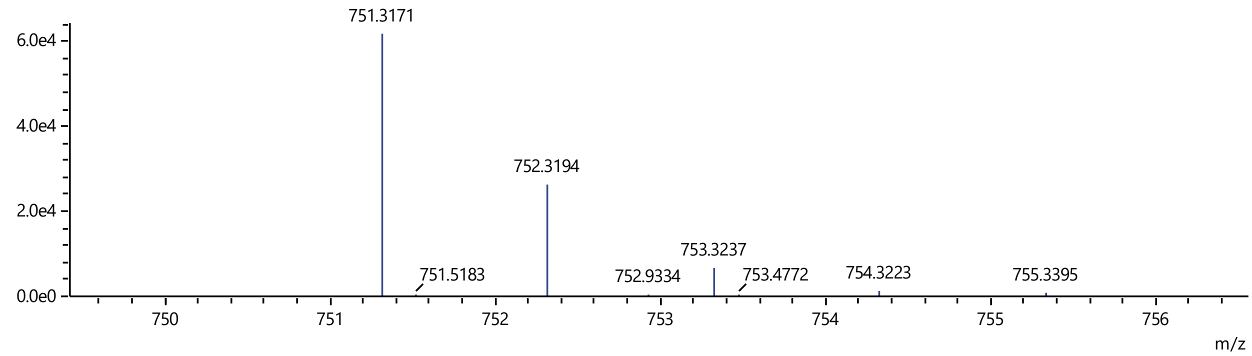

[C36H48N4O12+Na]+ 1.00e6

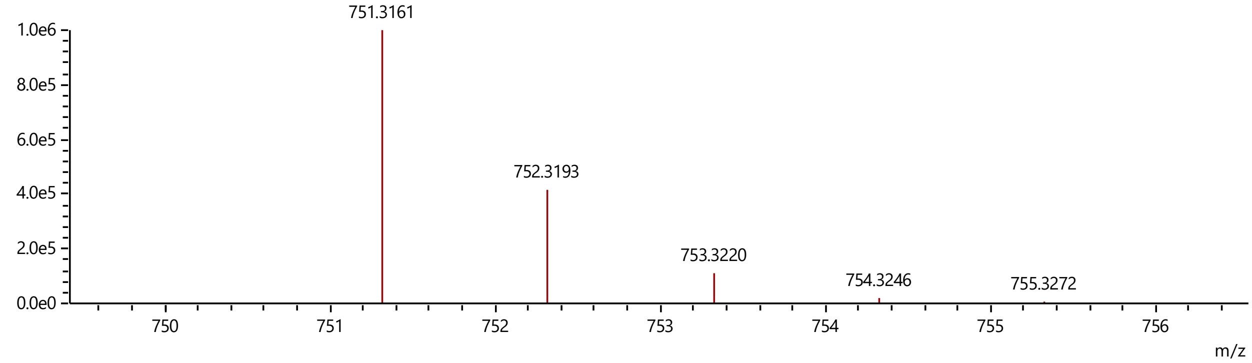

1:MS(-) RT:[0.332]-[0.100-0.150] 1.74e4

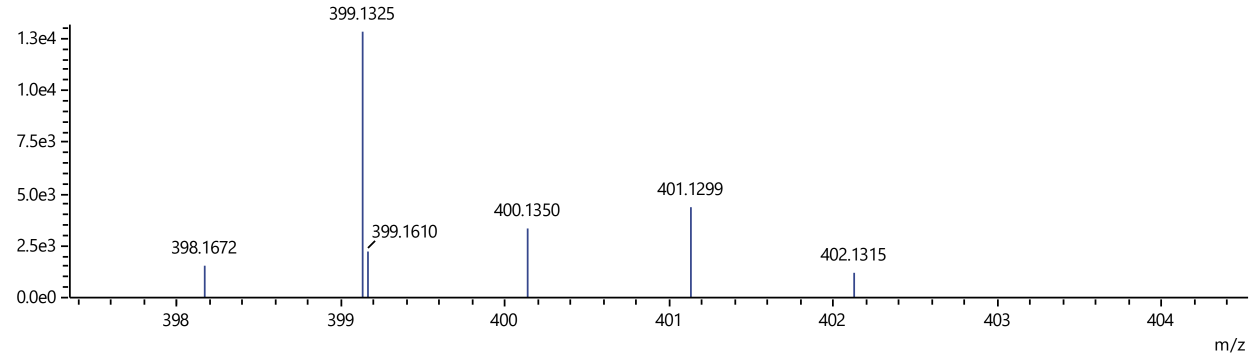

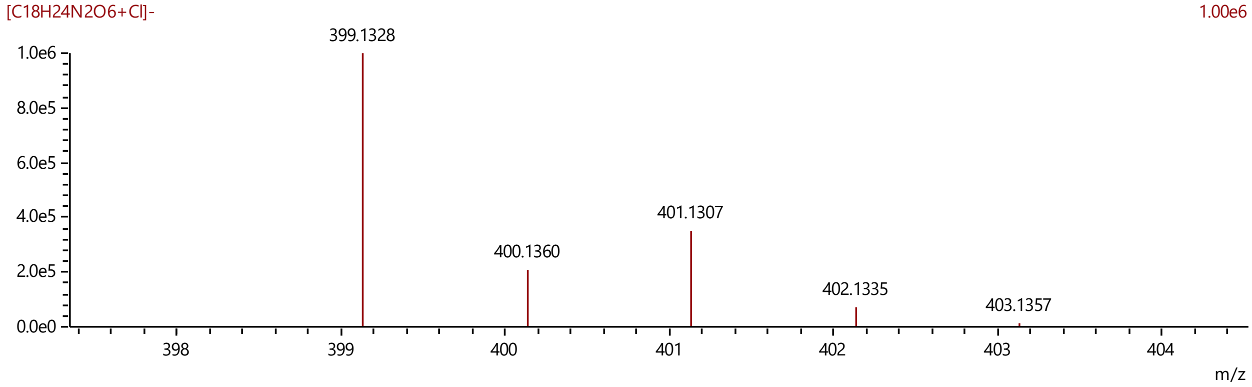

Figure S1c. HR-MS spectra of FOH-H

<u>DFA-T</u> HRMS (ESI): no data.

<u>FA-T</u> HRMS (ESI): calculated for $C_{19}H_{21}N_4O_4$ $[M+H]^+$ 369.1557, found: 369.1560; calculated for $C_{19}H_{20}N_4O_4Na$ $[M+Na]^+$ 391.1377, found: 391.1382; calculated for $C_{19}H_{19}N_4O_4$ $[M-H]^-$ 367.1412, found: 367.1415; calculated for $C_{19}H_{20}N_4O_4Cl$ $[M+H]^-$ 403.1179, found: 403.1183; calculated for $C_{19}H_{20}N_5O_7$ $[M+NO_3]^-$ 430.1368, found: 430.1363;

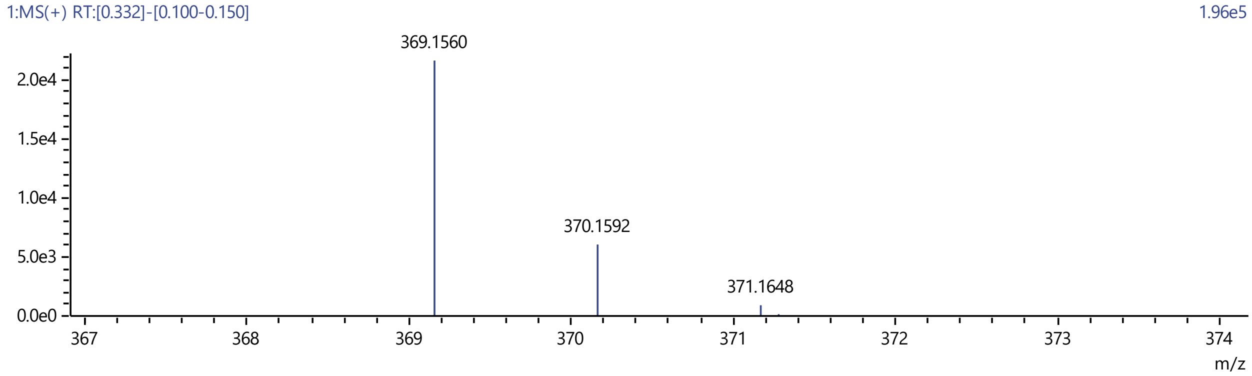

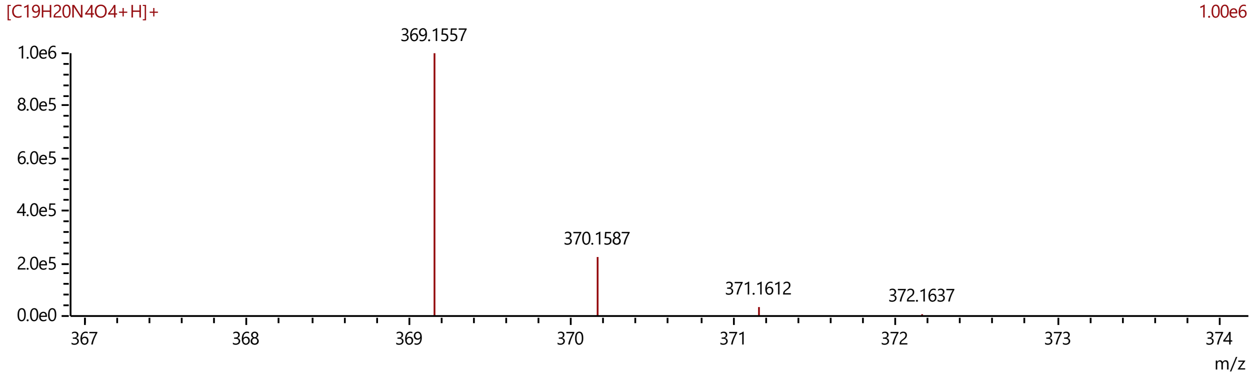

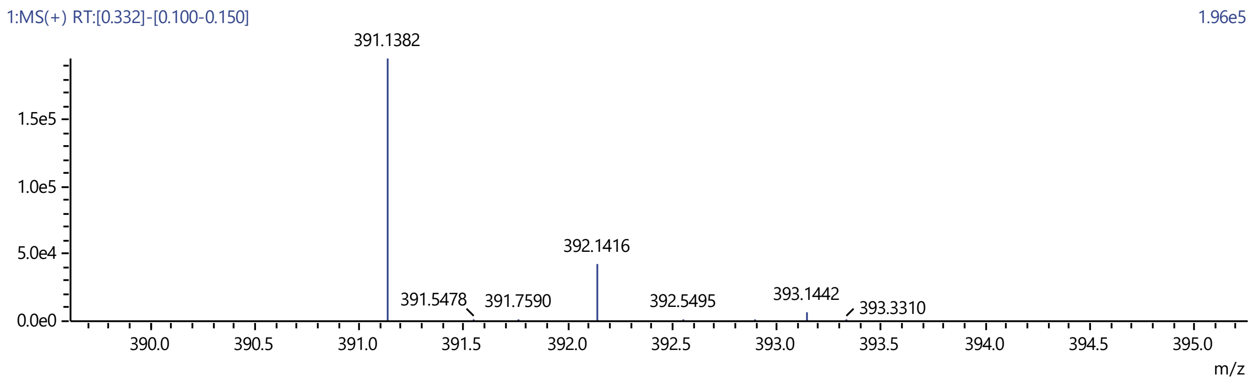
1:MS(+) RT:[0.332]-[0.100-0.150]
1.96e5
391.1382
392.1416
391.5478
391.7590
392.5495
393.1442
393.3310
m/z

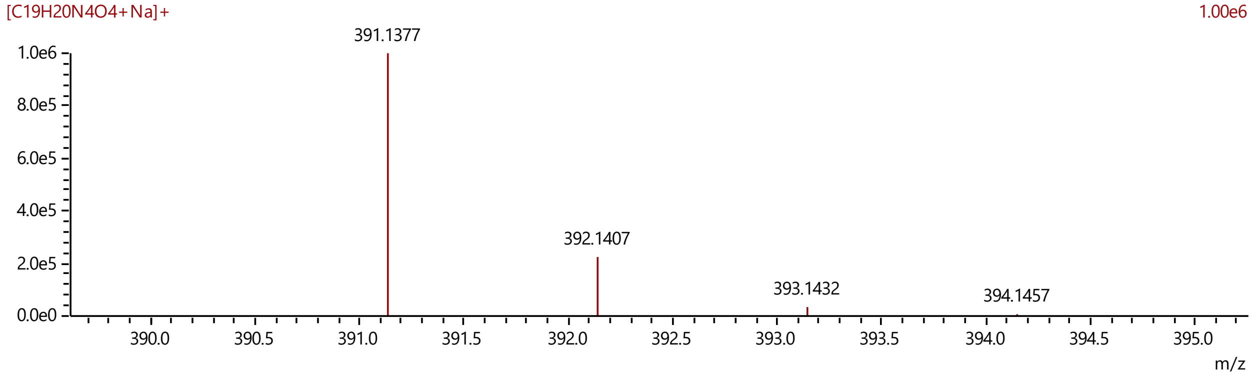
[C19H20N4O4+Na]+
1.00e6
391.1377
392.1407
393.1432
394.1457
m/z

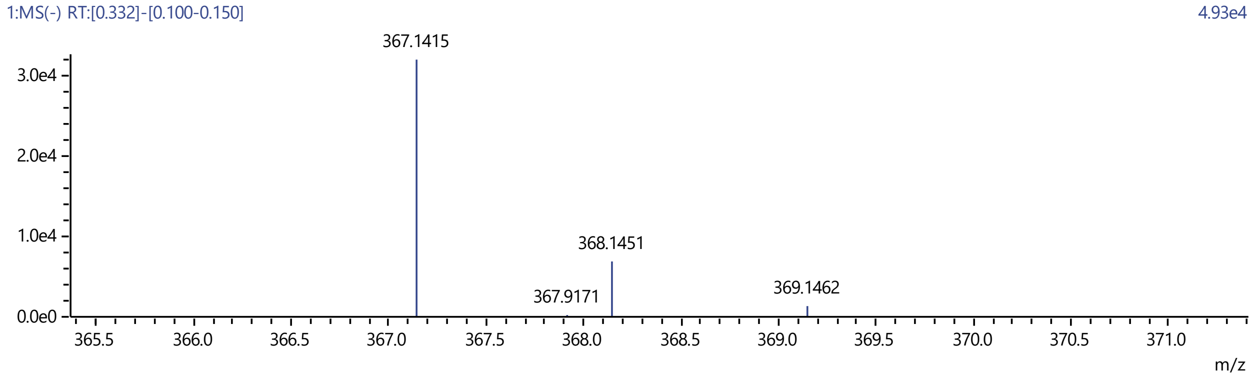
1:MS(-) RT:[0.332]-[0.100-0.150]
4.93e4
367.1415
368.1451
367.9171
369.1462
m/z

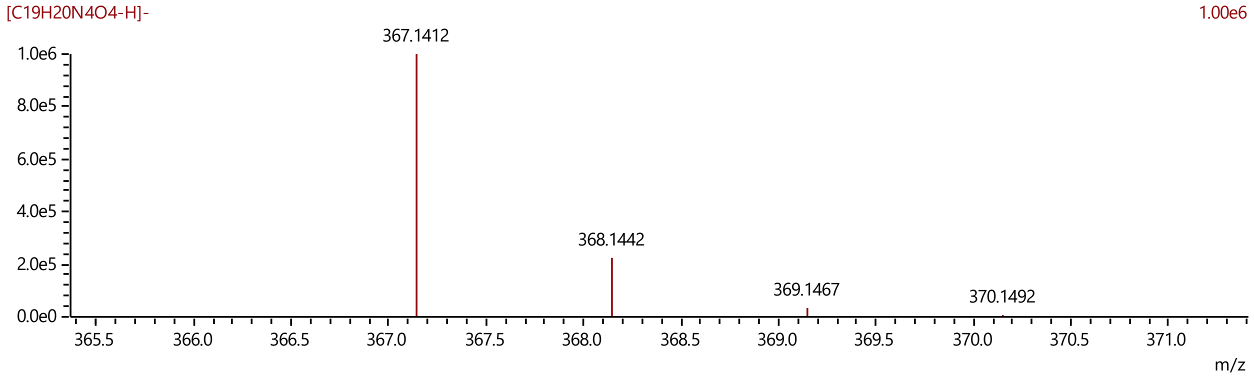
[C19H20N4O4-H]-
1.00e6
367.1412
368.1442
369.1467
370.1492
m/z

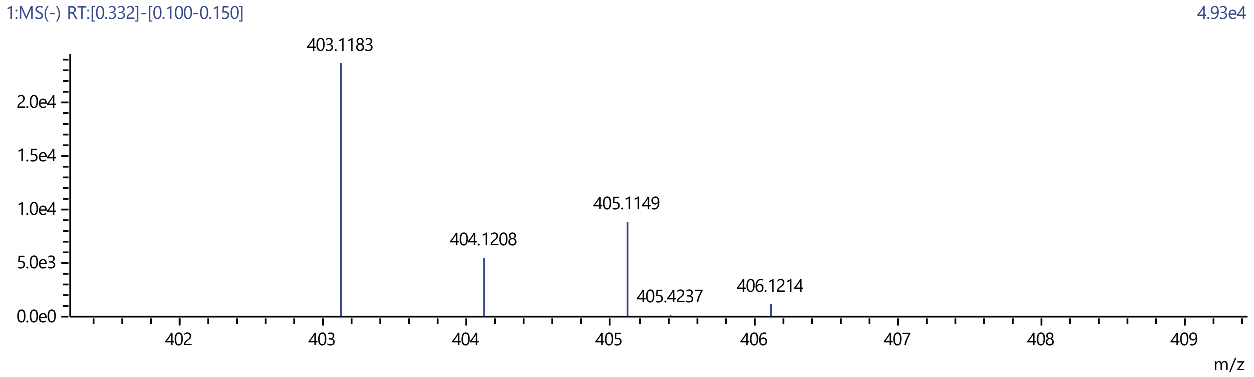
1:MS(-) RT:[0.332]-[0.100-0.150]
4.93e4
403.1183
404.1208
405.1149
405.4237
406.1214
m/z

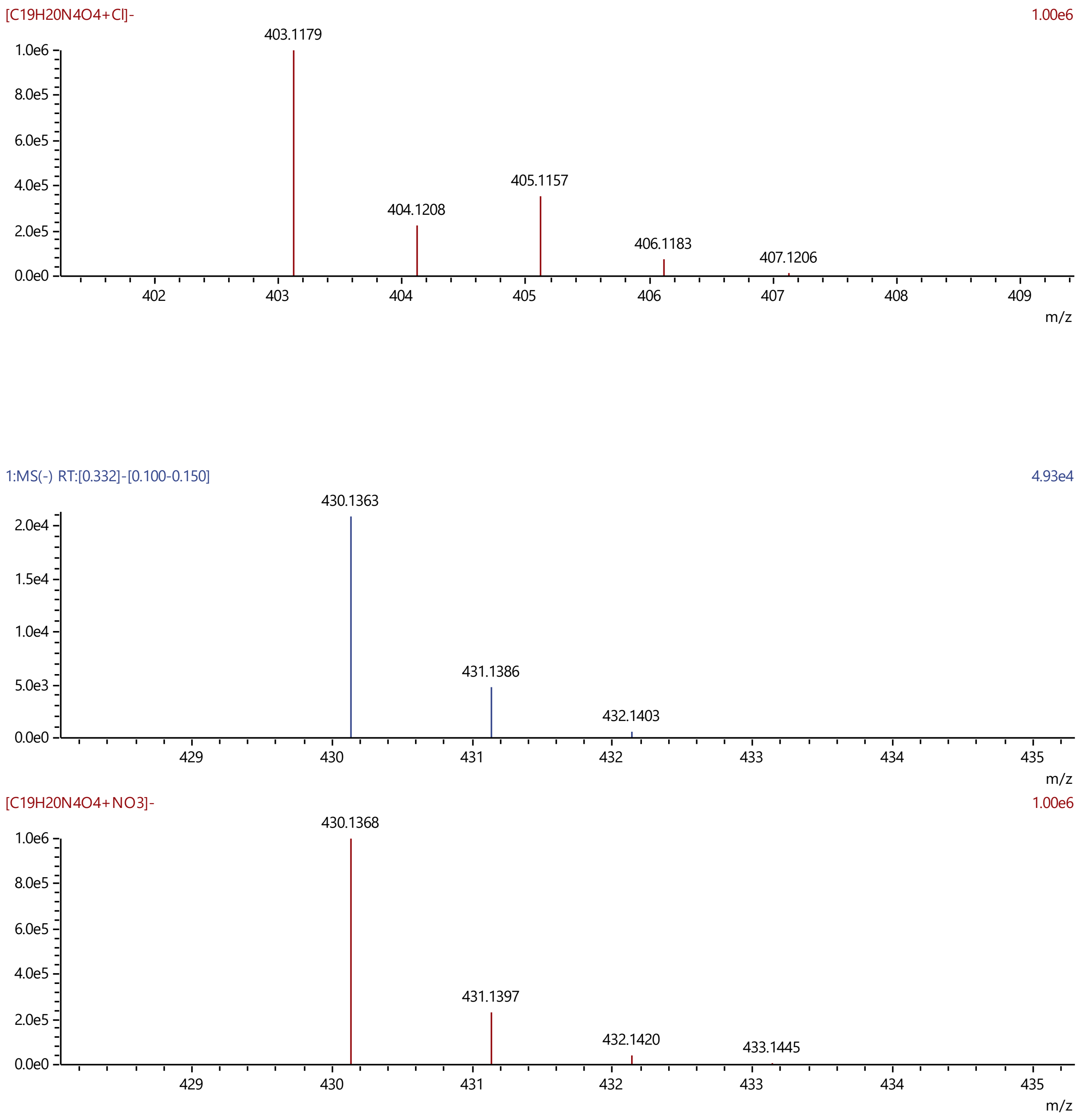

Figure S1d. HR-MS spectra of FA-T

<u>FOH-T</u> HRMS (ESI): calculated for $C_{19}H_{22}N_3O_6$ $[M+NH_4]^+$ 388.1503, found: 388.1510; calculated for $C_{19}H_{18}N_2O_6Na$ $[M+Na]^+$ 393.1057, found: 393.1065; calculated for $C_{19}H_{17}N_2O_6$ $[M-H]^-$ 369.1092, found: 369.1082; calculated for $C_{19}H_{18}N_2O_6Cl$ $[M+Cl]^-$ 405.0859, found: 405.0862.

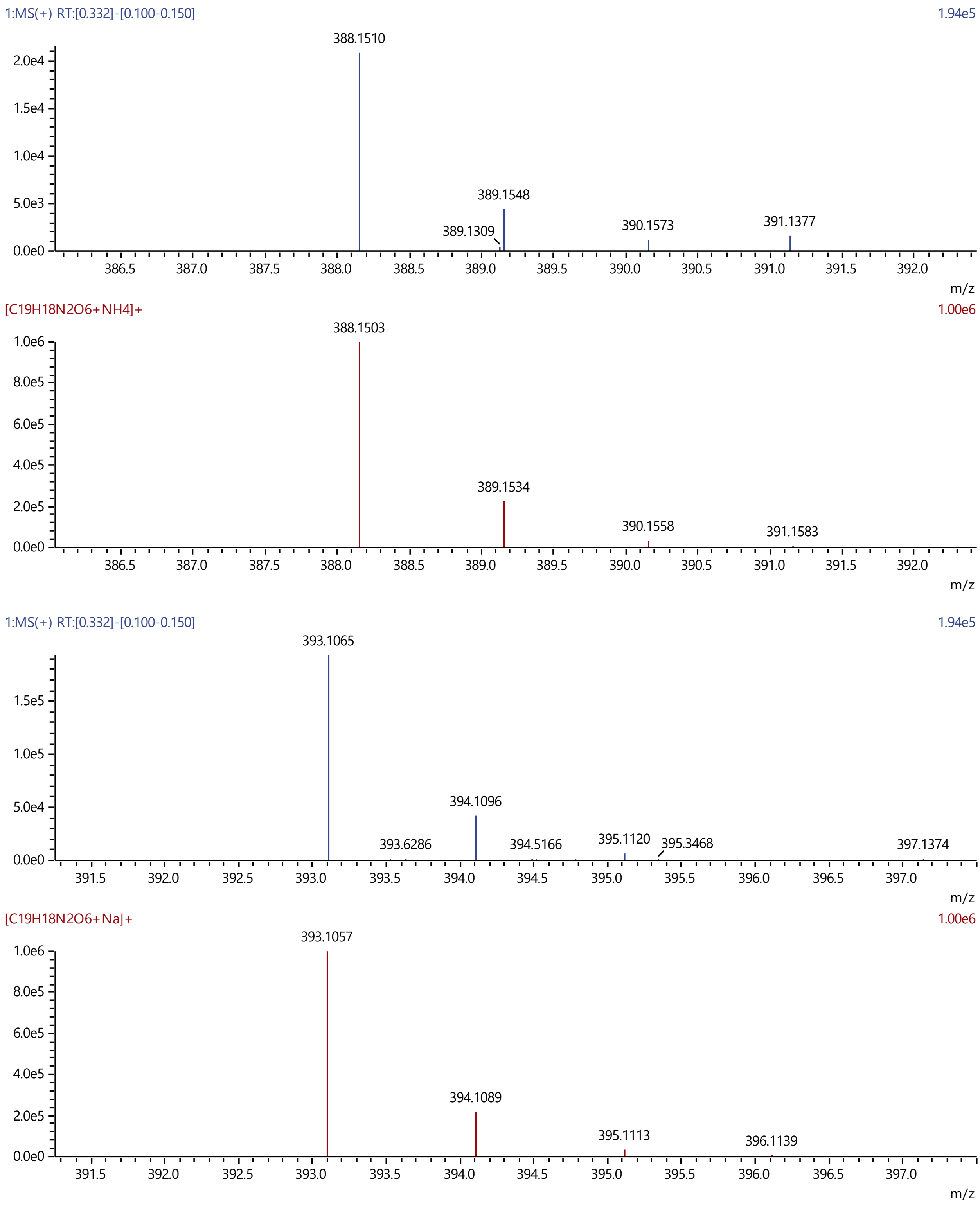
1:MS(+) RT:[0.332]-[0.100-0.150]
1.94e5
388.1510
389.1548
389.1309
390.1573
391.1377
m/z
[C19H18N2O6+NH4]+
1.00e6
388.1503
389.1534
390.1558
391.1583
m/z
1:MS(+) RT:[0.332]-[0.100-0.150]
1.94e5
393.1065
394.1096
393.6286
394.5166
395.1120
395.3468
397.1374
m/z
[C19H18N2O6+Na]+
1.00e6
393.1057
394.1089
395.1113
396.1139
m/z

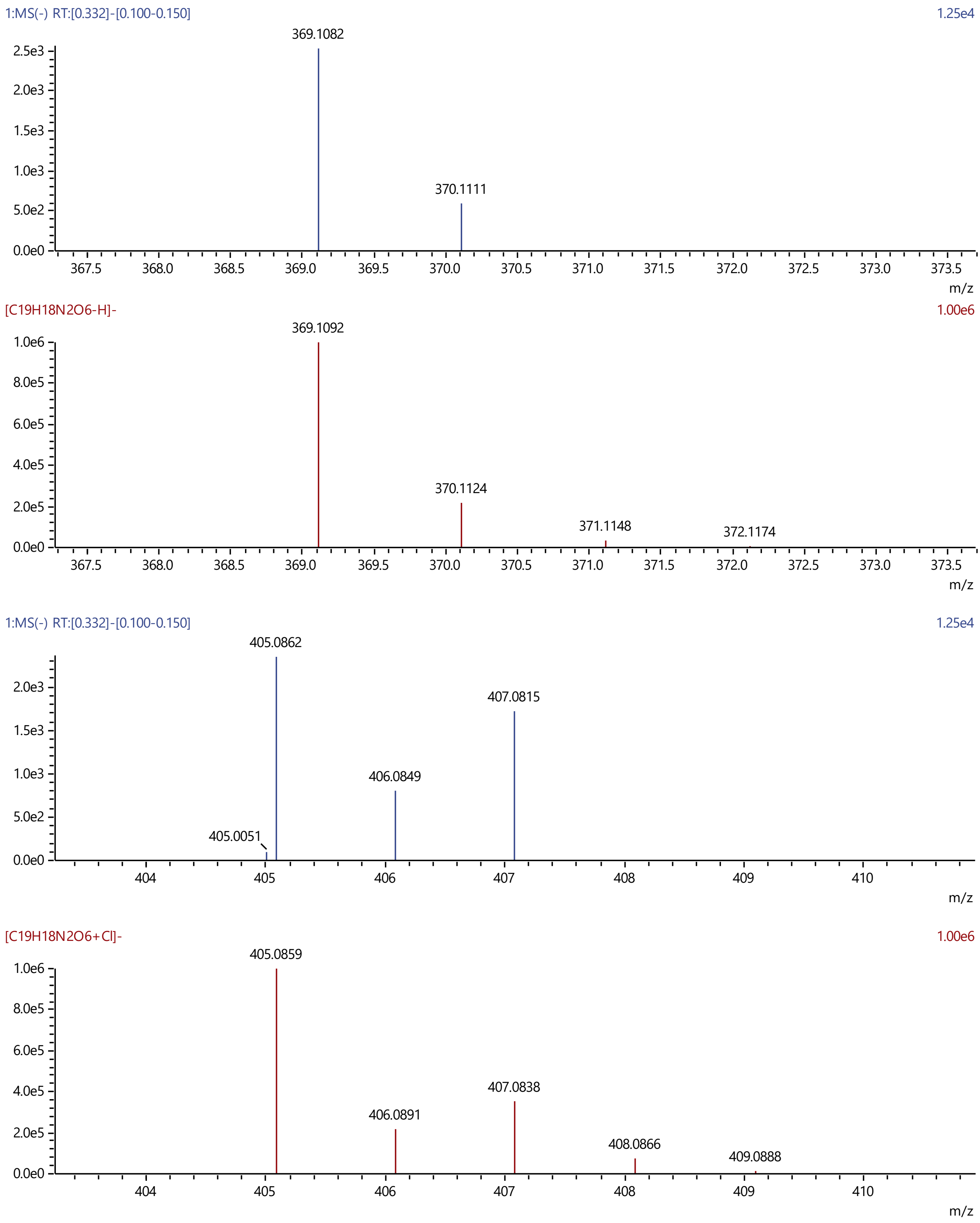

Figure S1e. HR-MS spectra of FOH-T

DFA-M HRMS (ESI): calculated for $C_{35}H_{31}N_4O_6$ $[M-H]^-$ 603.2249, found: 603.2253.

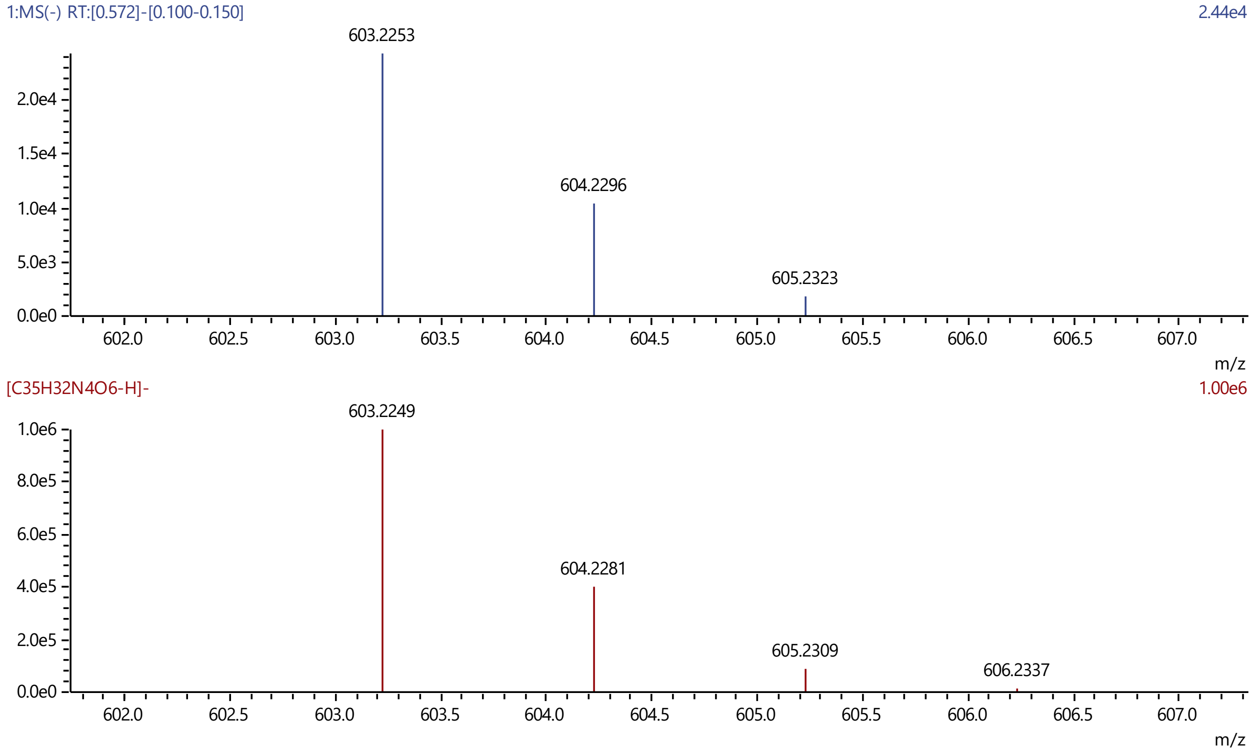

Figure S1f. HR-MS spectra of DFA-M

<u>FA-M</u> HRMS (ESI): calculated for $C_{25}H_{28}N_5O_4$ $[M+NH_4]^+$ 462.2136, found: 462.2137; calculated for $C_{25}H_{24}N_4O_4Na$ $[M+Na]^+$ 467.1690, found: 467.1698; calculated for $C_{25}H_{23}N_4O_4$ $[M-H]^-$ 443.1725, found: 443.1730; calculated for $C_{25}H_{24}N_4O_4Cl$ $[M+Cl]^-$ 479.1492, found: 479.1495.

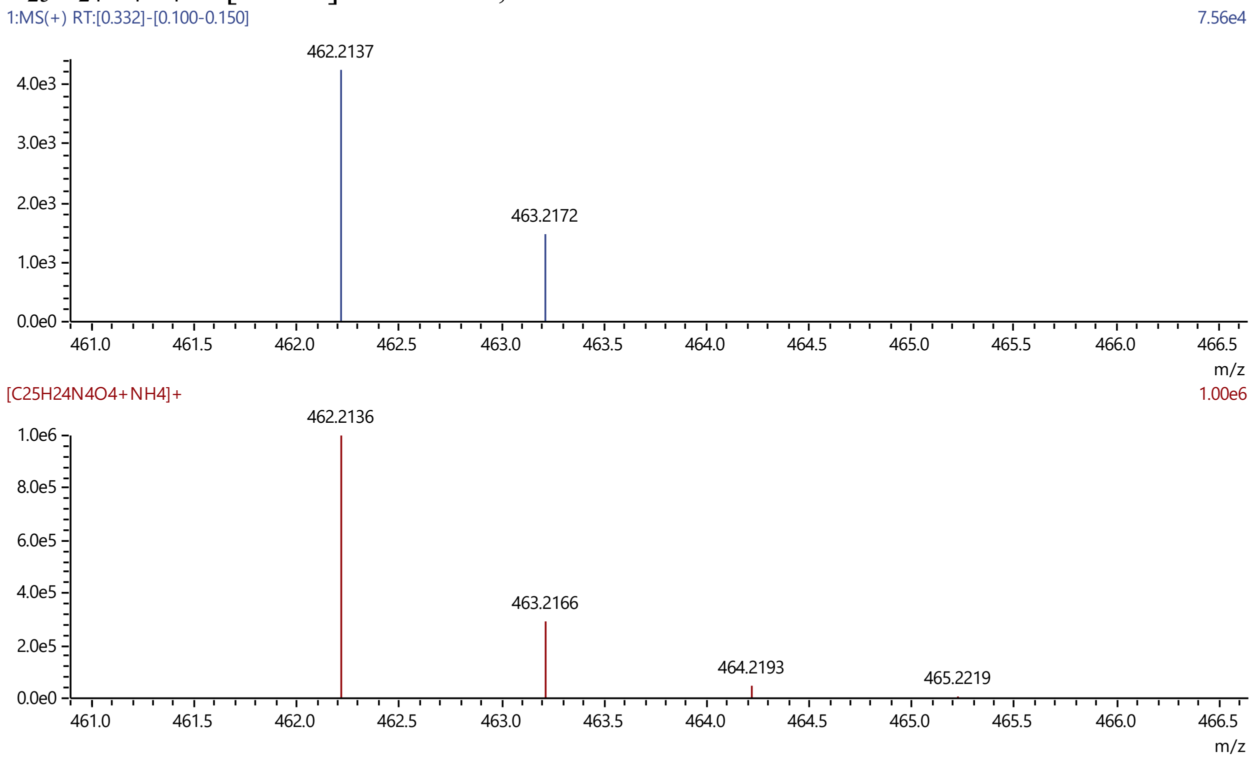

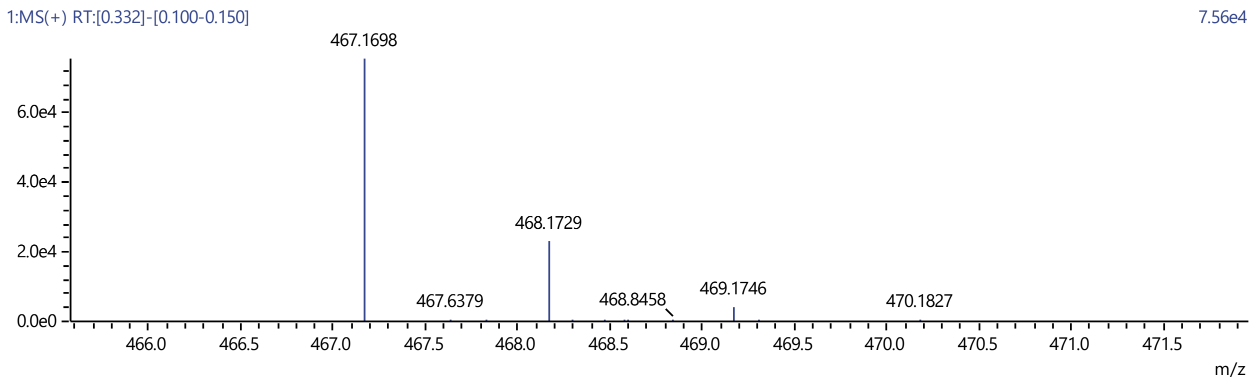

1:MS(+) RT:[0.332]-[0.100-0.150]
7.56e4
467.1698
468.1729
467.6379
468.8458
469.1746
470.1827
m/z

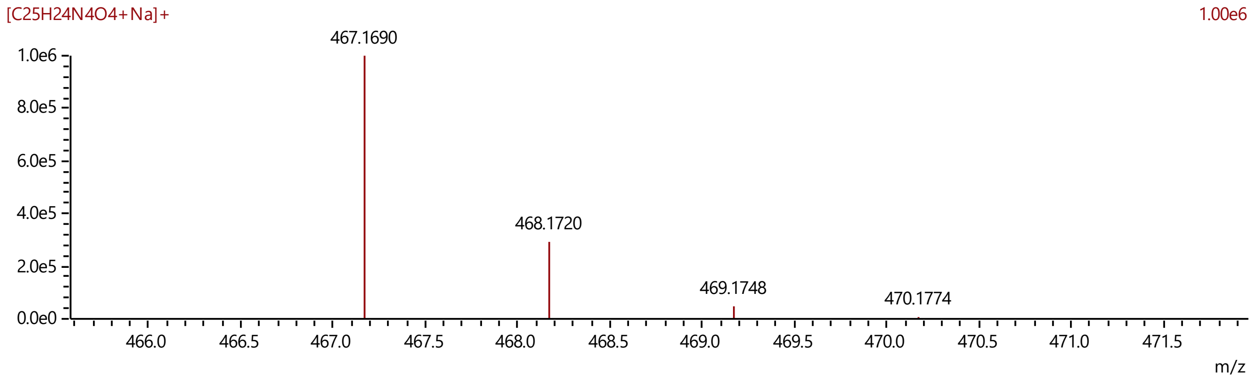

[C25H24N4O4+Na]+
1.00e6
467.1690
468.1720
469.1748
470.1774
m/z

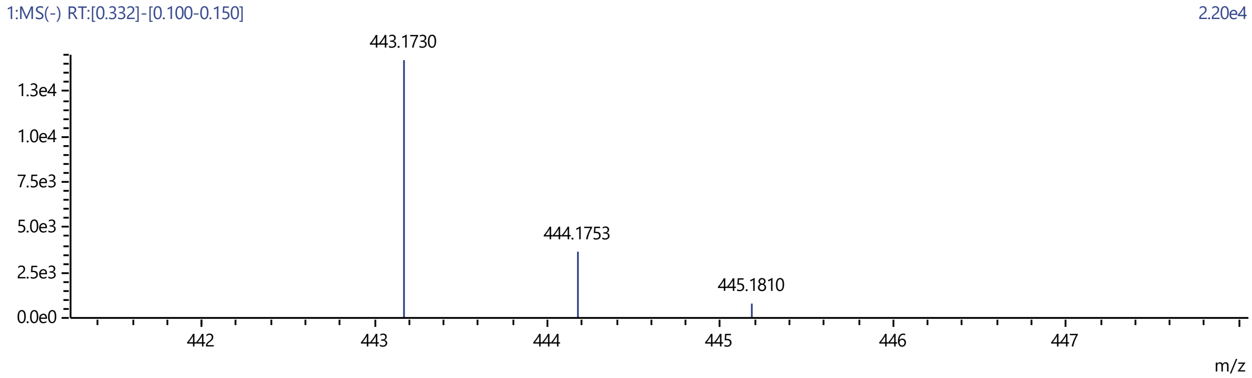

1:MS(-) RT:[0.332]-[0.100-0.150]
2.20e4
443.1730
444.1753
445.1810
m/z

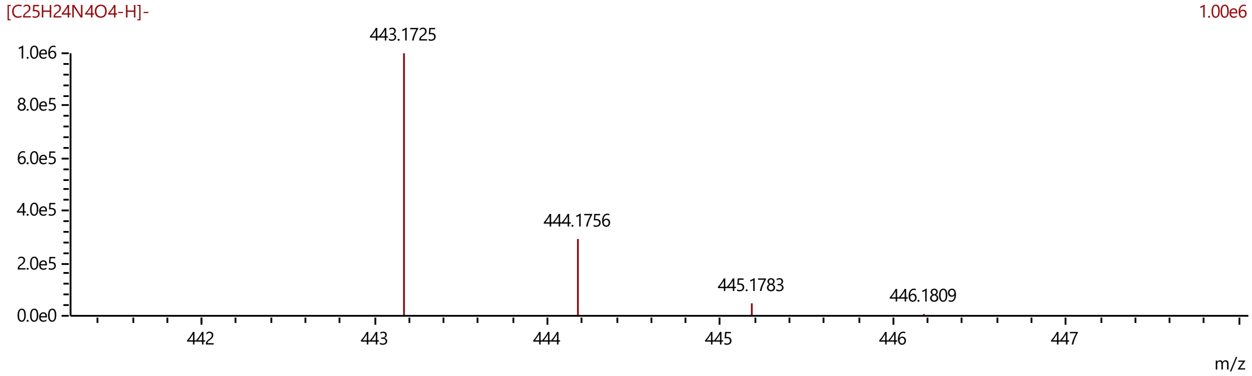

[C25H24N4O4-H]-
1.00e6
443.1725
444.1756
445.1783
446.1809
m/z

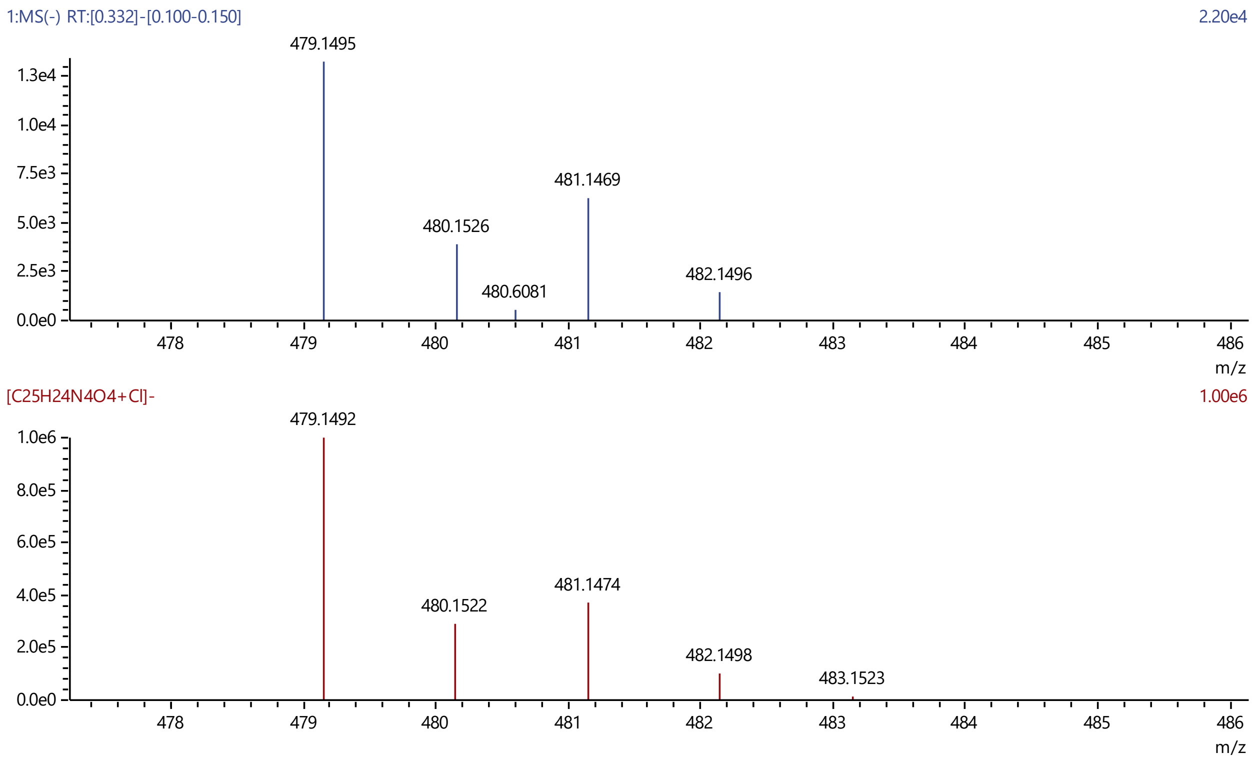

Figure S1g. HR-MS spectra of FA-M

<u>FOH-M</u> HRMS (ESI): calculated for $C_{25}H_{26}N_3O_6$ $[M+NH_4]^+$ 464.1816, found: 464.1823; calculated for $C_{25}H_{22}N_2O_6Na$ $[M+Na]^+$ 469.1370, found: 469.1378; calculated for $C_{50}H_{44}N_4O_{12}Na$ $[2M+Na]^+$ 915.2848, found: 915.2858; calculated for $C_{25}H_{21}N_2O_6$ $[M-H]^-$ 445.1405, found: 445.1409.

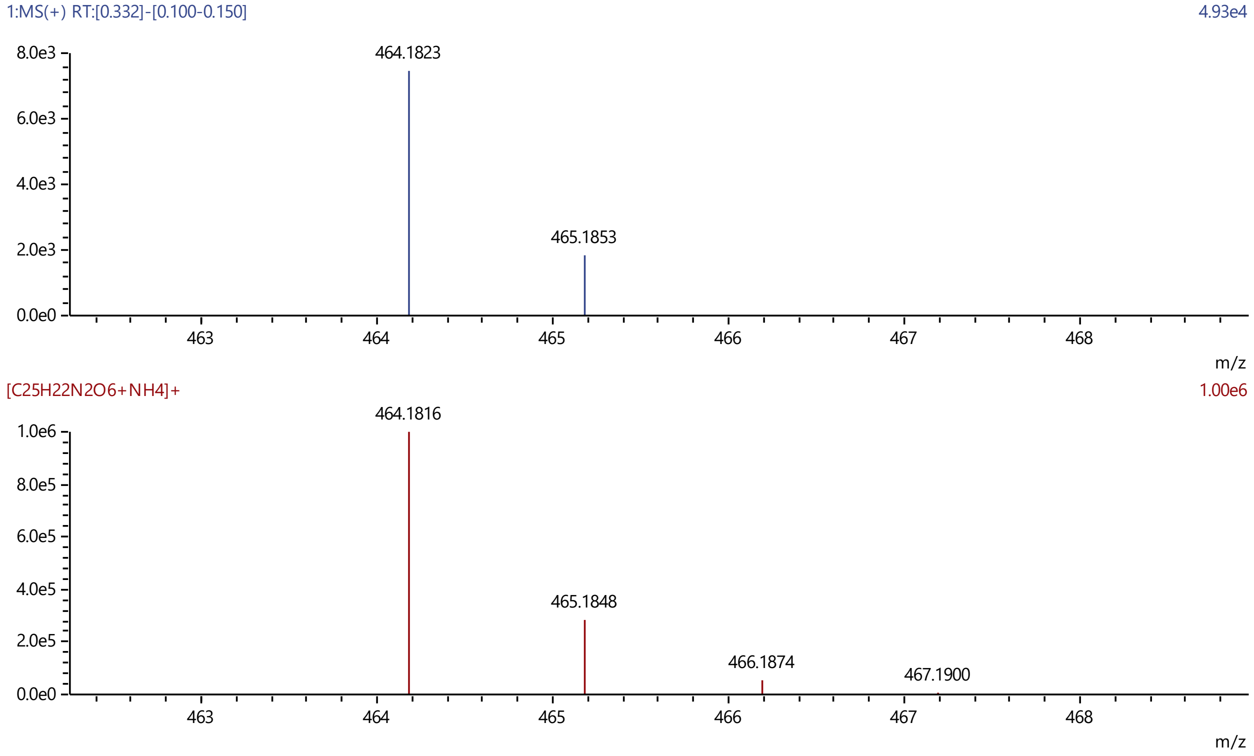

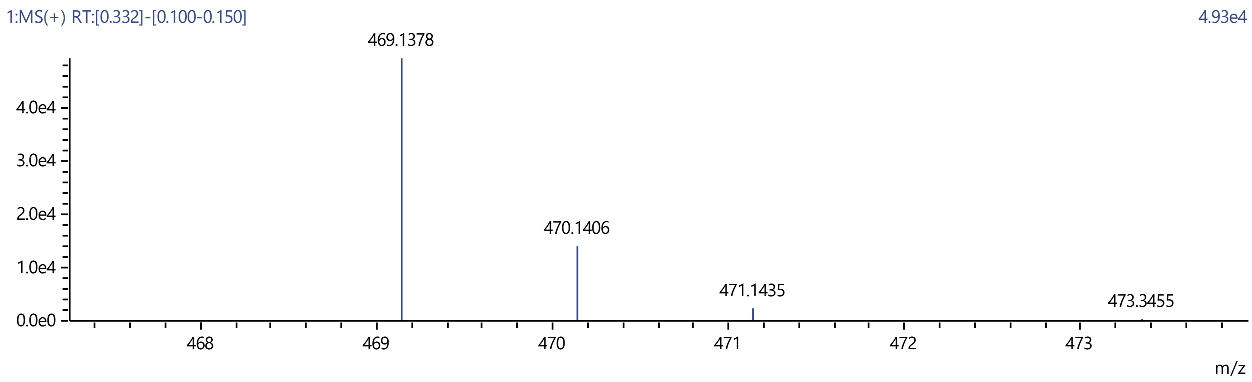
1:MS(+) RT:[0.332]-[0.100-0.150]
4.93e4
469.1378
470.1406
471.1435
473.3455
0.0e0
1.0e4
2.0e4
3.0e4
4.0e4
468
469
470
471
472
473
m/z

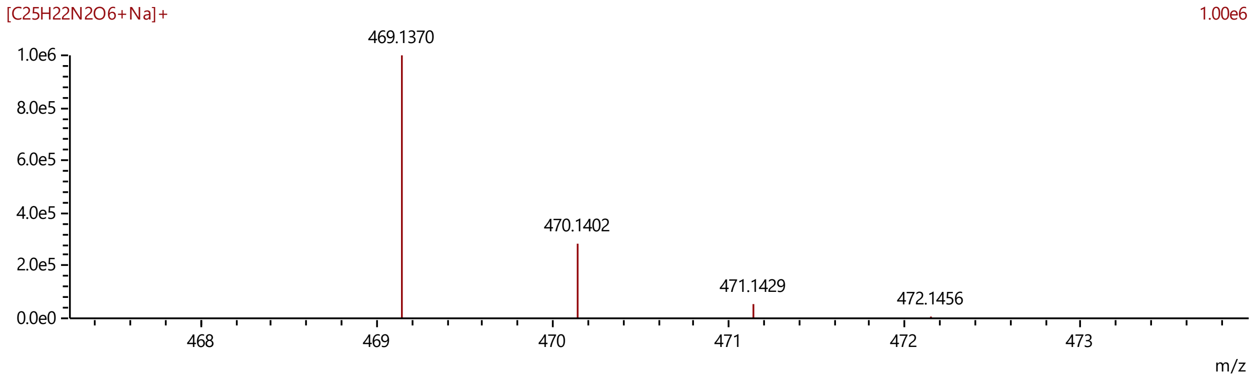
[C25H22N2O6+Na]+
1.00e6
469.1370
470.1402
471.1429
472.1456
0.0e0
2.0e5
4.0e5
6.0e5
8.0e5
1.0e6
468
469
470
471
472
473
m/z

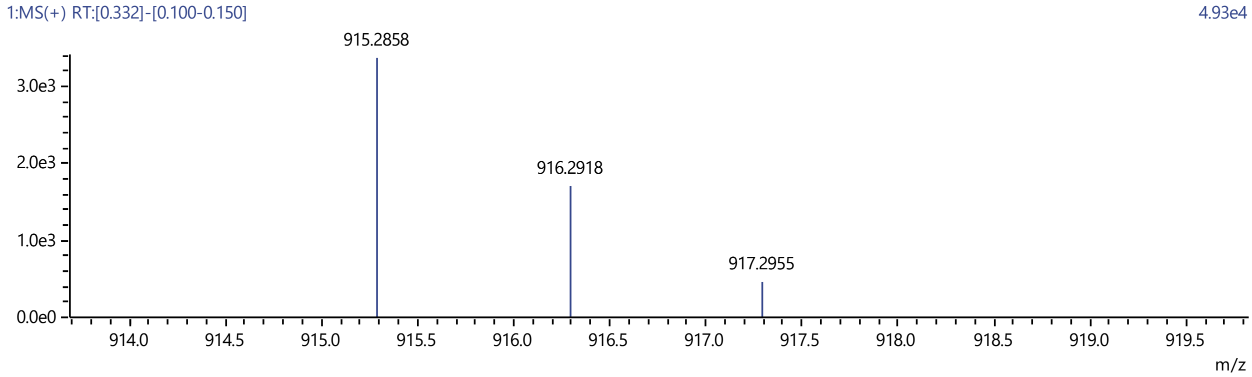
1:MS(+) RT:[0.332]-[0.100-0.150]
4.93e4
915.2858
916.2918
917.2955
0.0e0
1.0e3
2.0e3
3.0e3
914.0
914.5
915.0
915.5
916.0
916.5
917.0
917.5
918.0
918.5
919.0
919.5
m/z

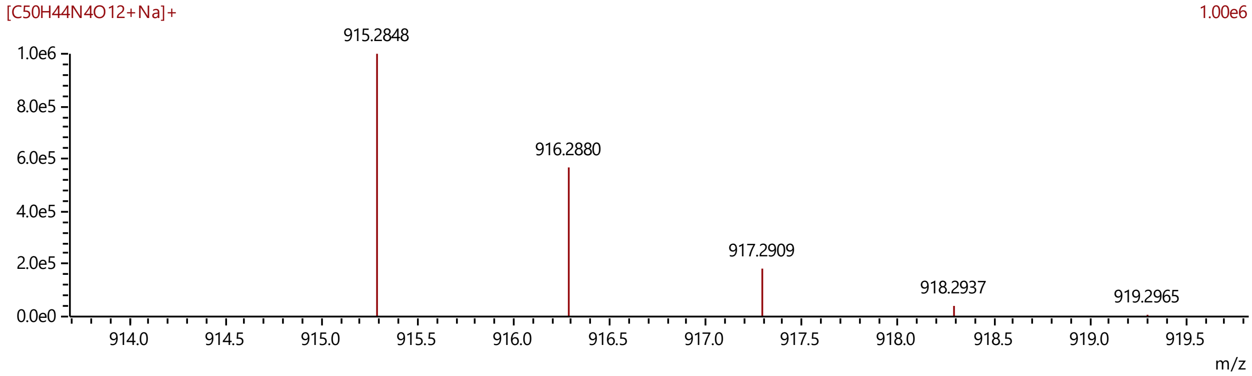
[C50H44N4O12+Na]+
1.00e6
915.2848
916.2880
917.2909
918.2937
919.2965
0.0e0
2.0e5
4.0e5
6.0e5
8.0e5
1.0e6
914.0
914.5
915.0
915.5
916.0
916.5
917.0
917.5
918.0
918.5
919.0
919.5
m/z

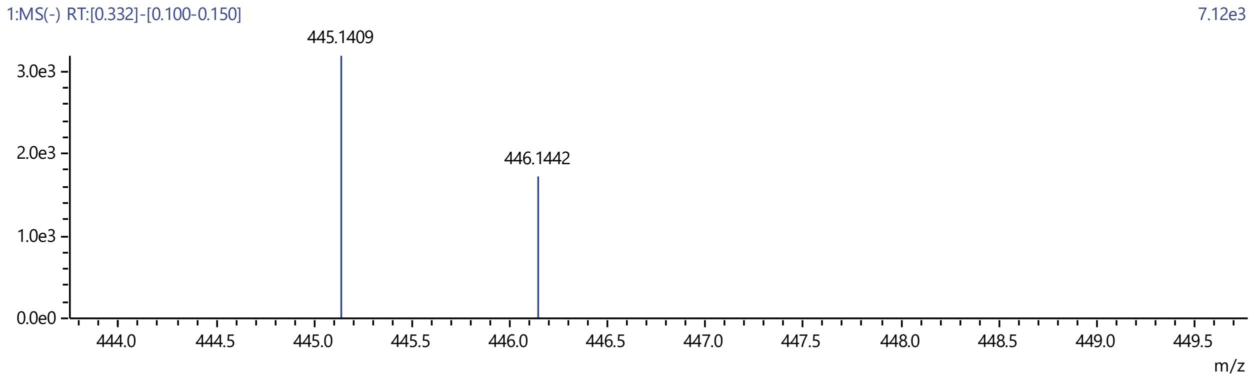
1:MS(-) RT:[0.332]-[0.100-0.150]
7.12e3
445.1409
446.1442
0.0e0
1.0e3
2.0e3
3.0e3
444.0
444.5
445.0
445.5
446.0
446.5
447.0
447.5
448.0
448.5
449.0
449.5
m/z

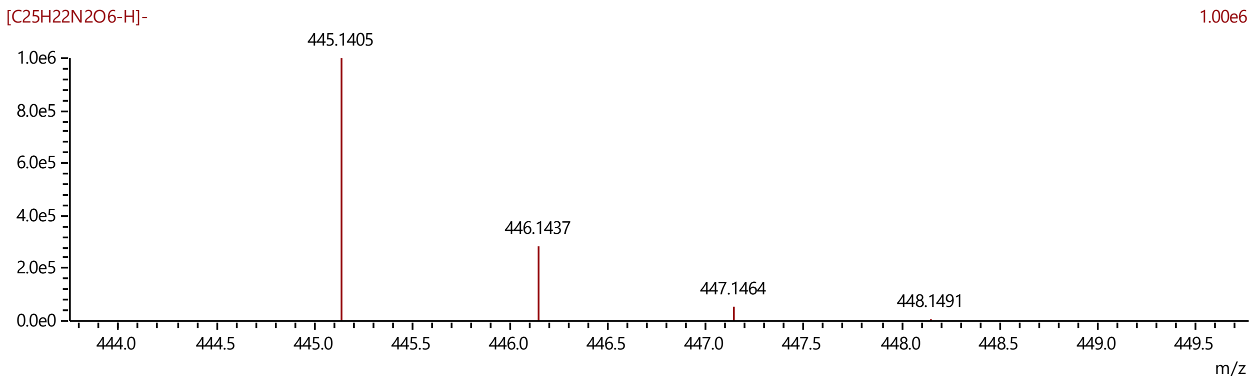

Figure S1h. HR-MS spectra of FOH-M

2 Nuclear Magnetic Resonance (NMR)

NMR spectra were recorded by a Bruker Avance 600 NMR Spectrometer (600.1 MHz), using residual proton signal of the deuterated solvent as a reference, chemical shifts were reported as parts per million downfield from tetramethylsilane (TMS).

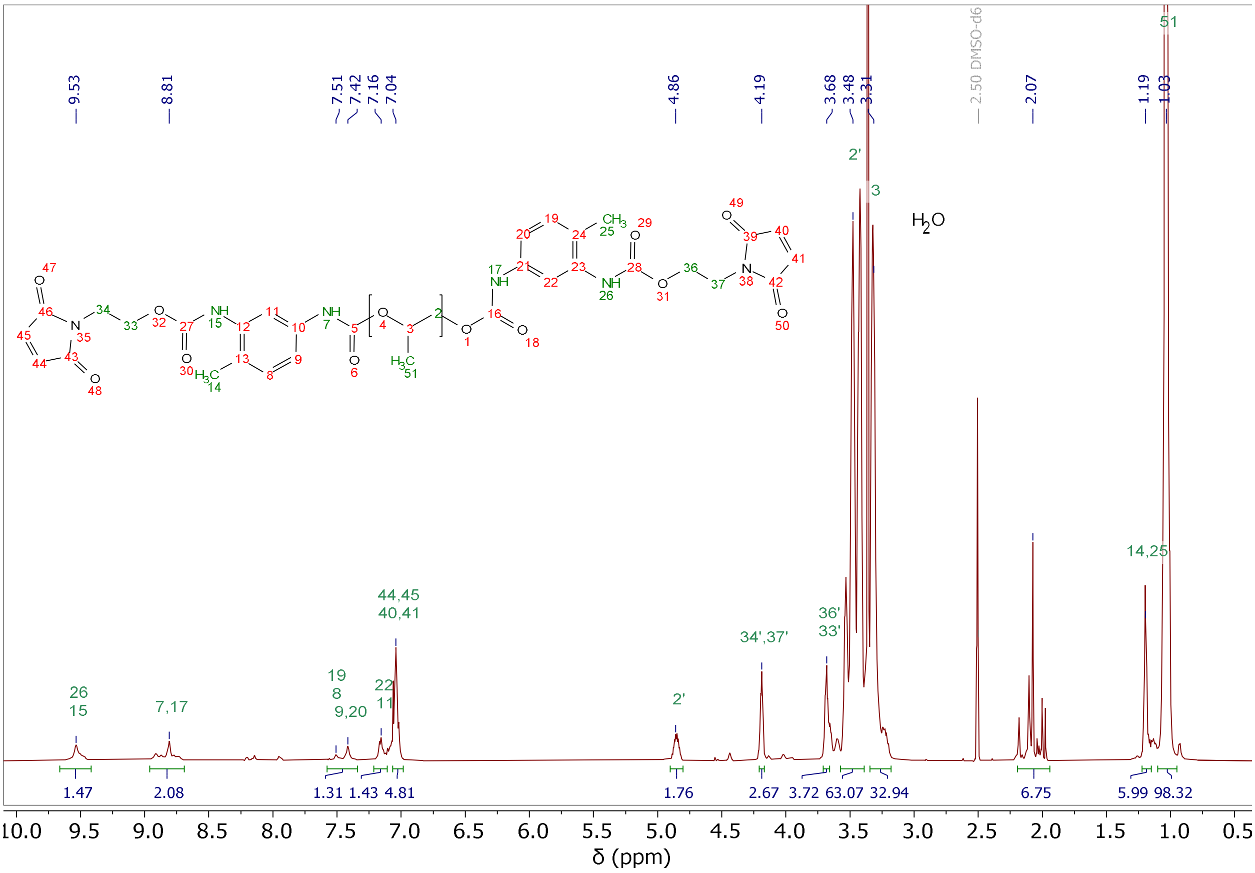

Figure S2a. ^{1}H NMR spectrum of the PU prepolymer in DMSO-d_6.

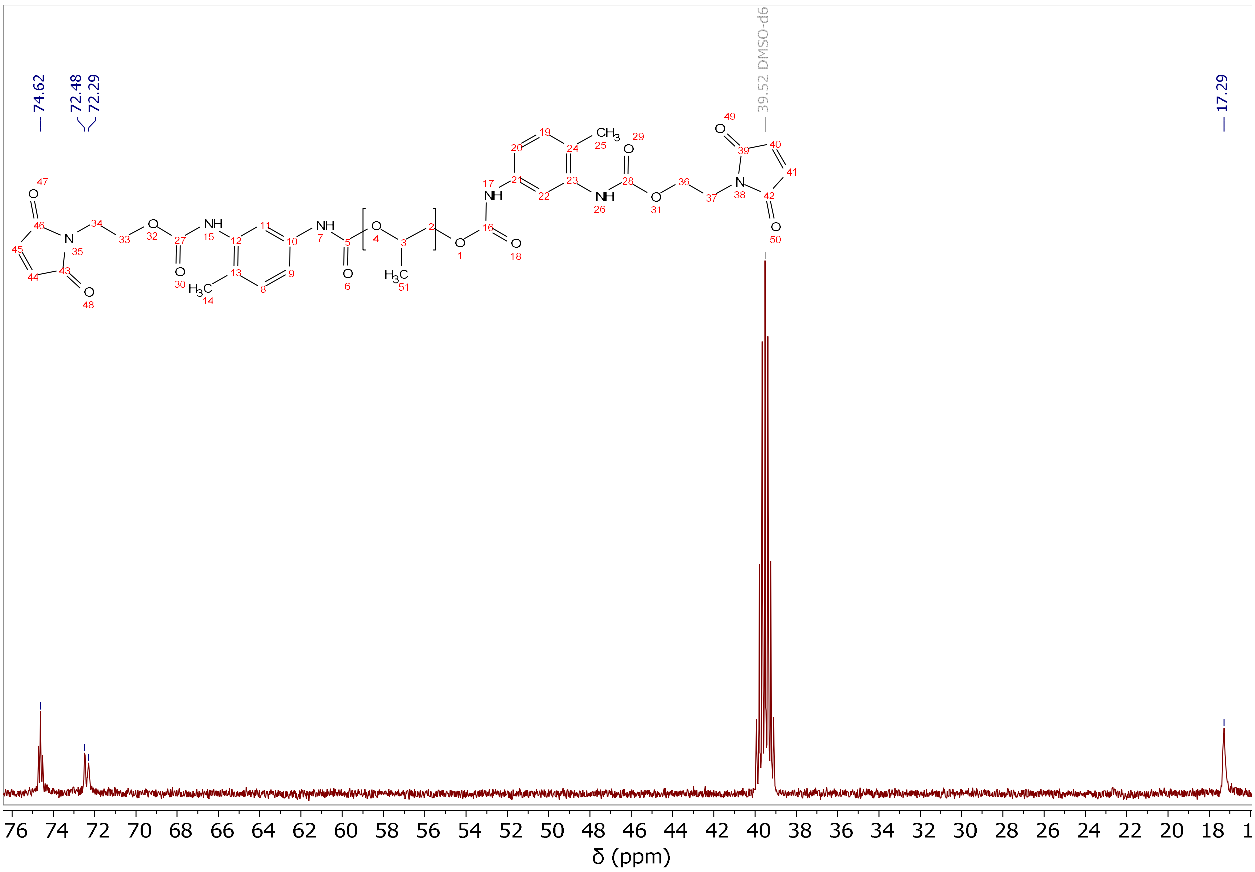

Figure S2b. ^{13}C NMR spectrum of the PU prepolymer in DMSO-d_6.

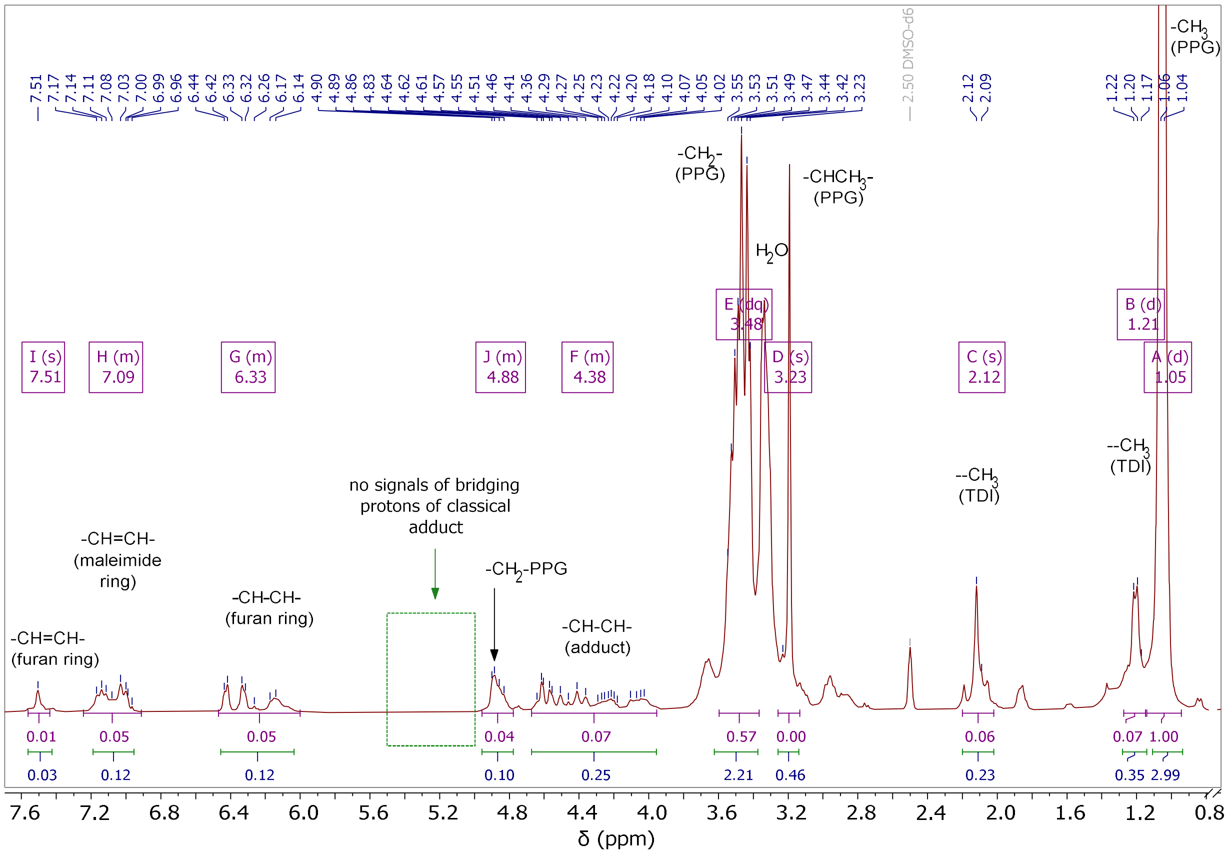

Figure S2c. ^{1}H NMR spectrum of the PU-H1 in DMSO-d_6.

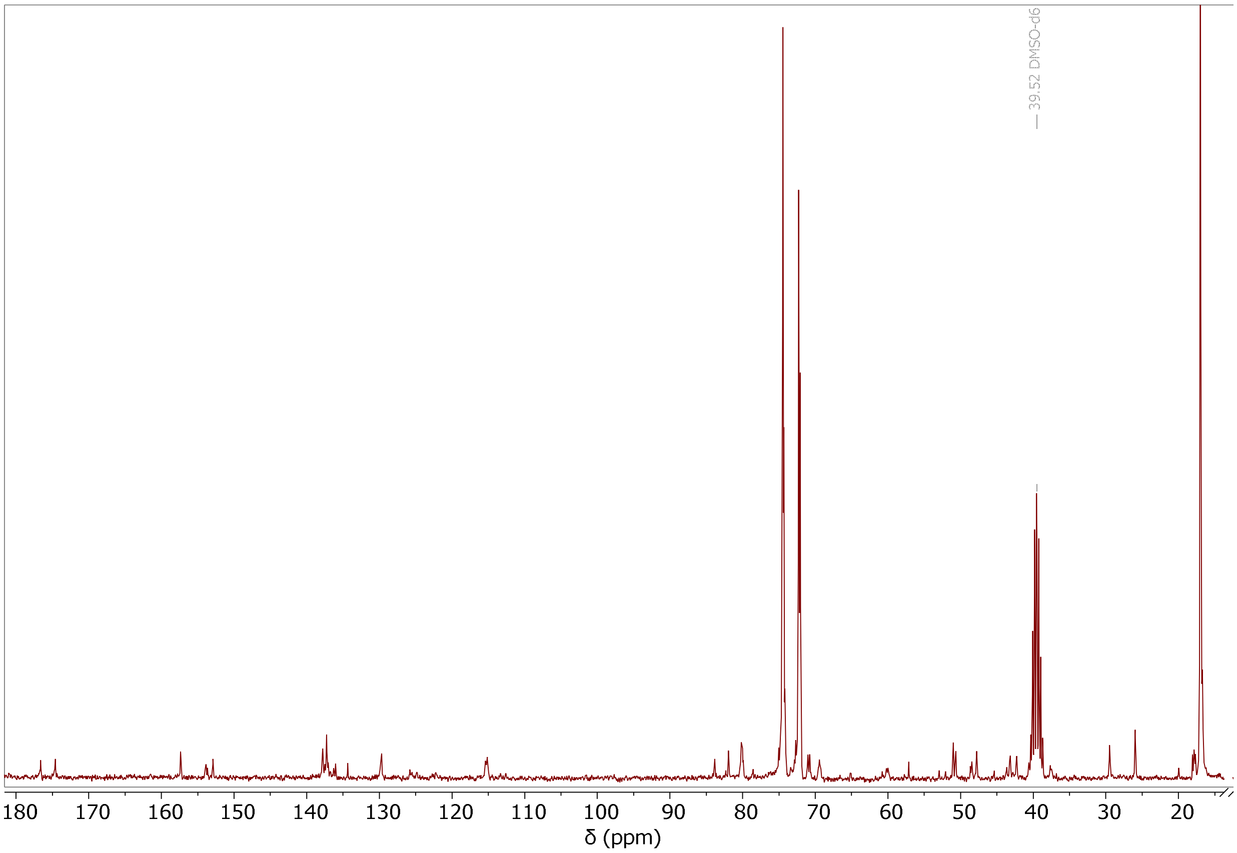

Figure S2d. ^{13}C NMR spectrum of the PU-H1 in DMSO-d_6.

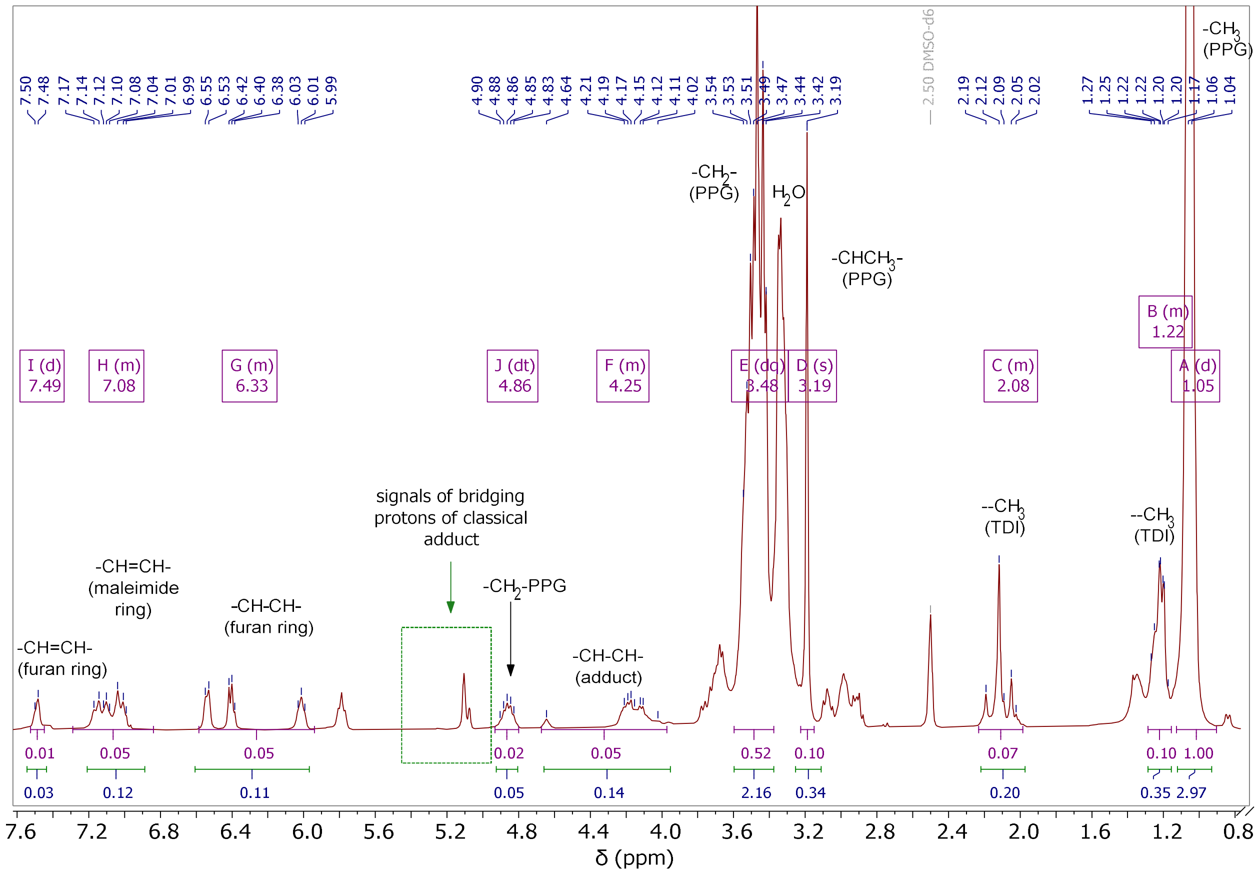

Figure S2e. ^{1}H NMR spectrum of the PU-H2 in DMSO-d_6.

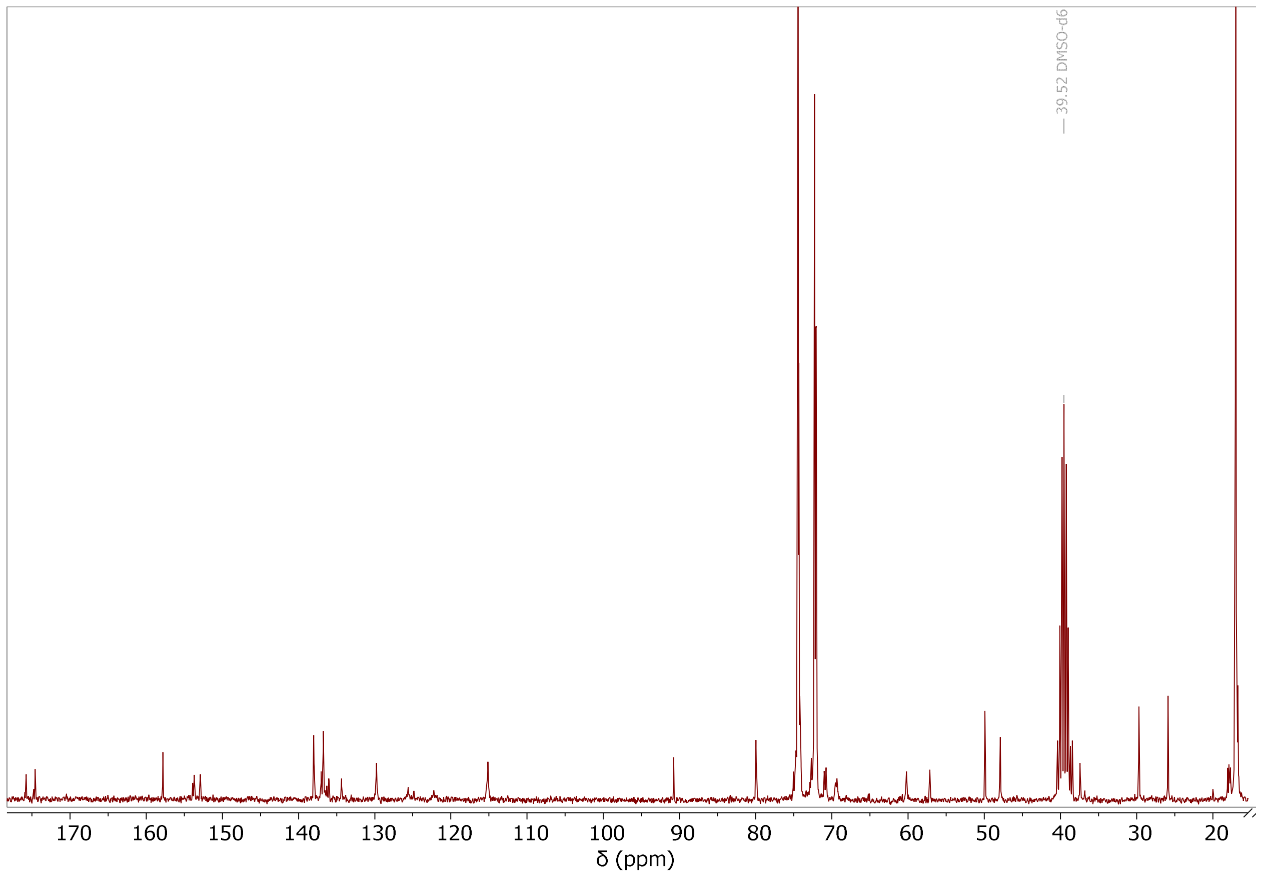

Figure S2f. ^{13}C NMR spectrum of the PU-H2 in DMSO-d_6.

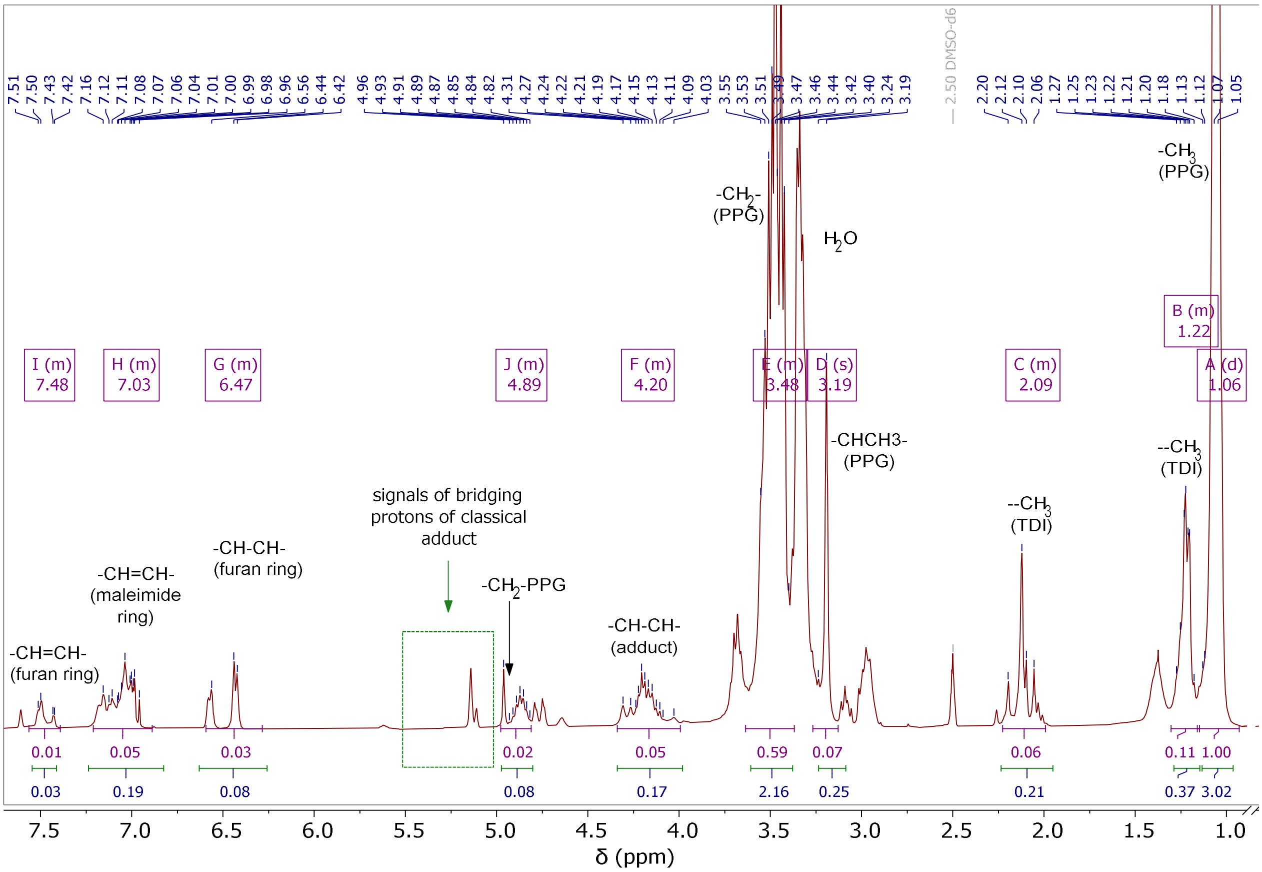

Figure S2g. ^{1}H NMR spectrum of the PU-H3 in DMSO-d_6.

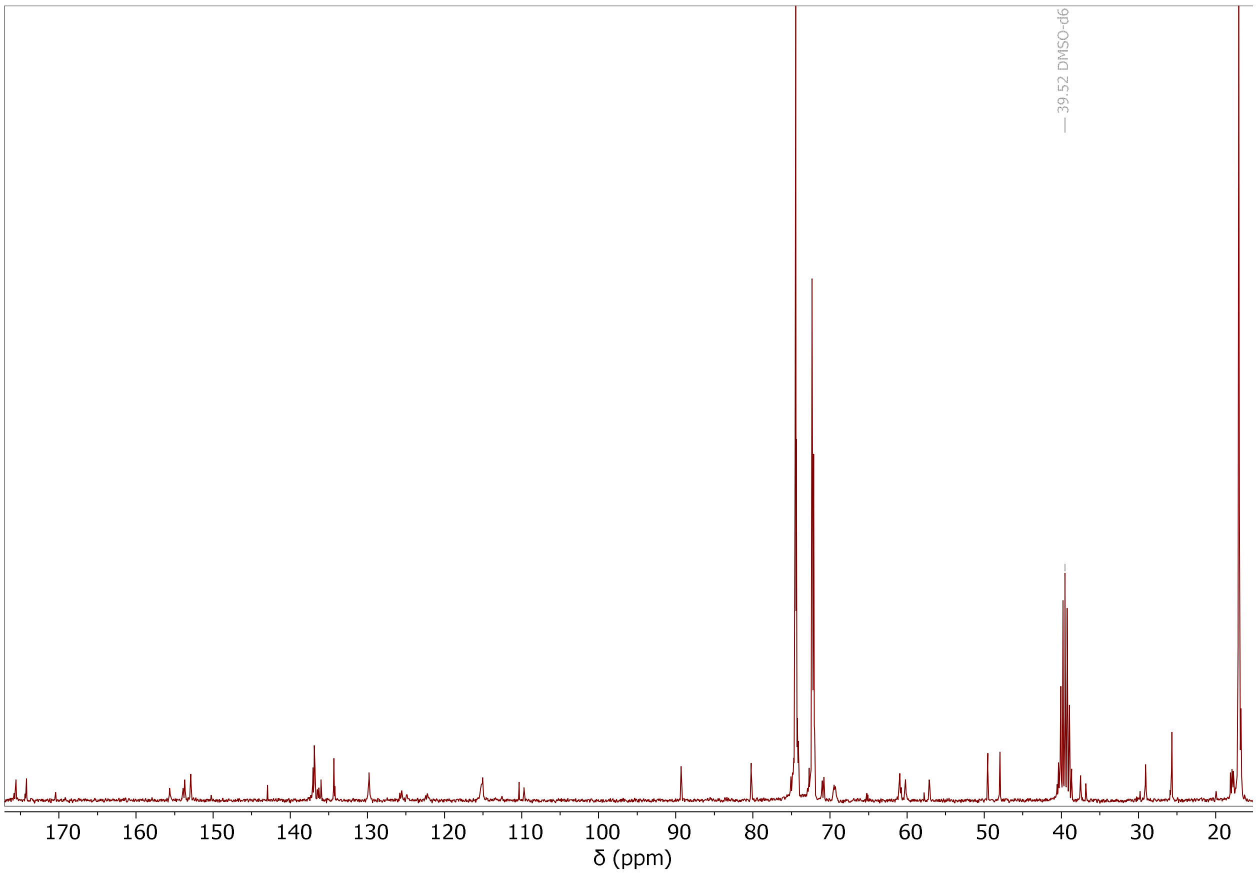

Figure S2h. ^{13}C NMR spectrum of the PU-H3 in DMSO-d_6.

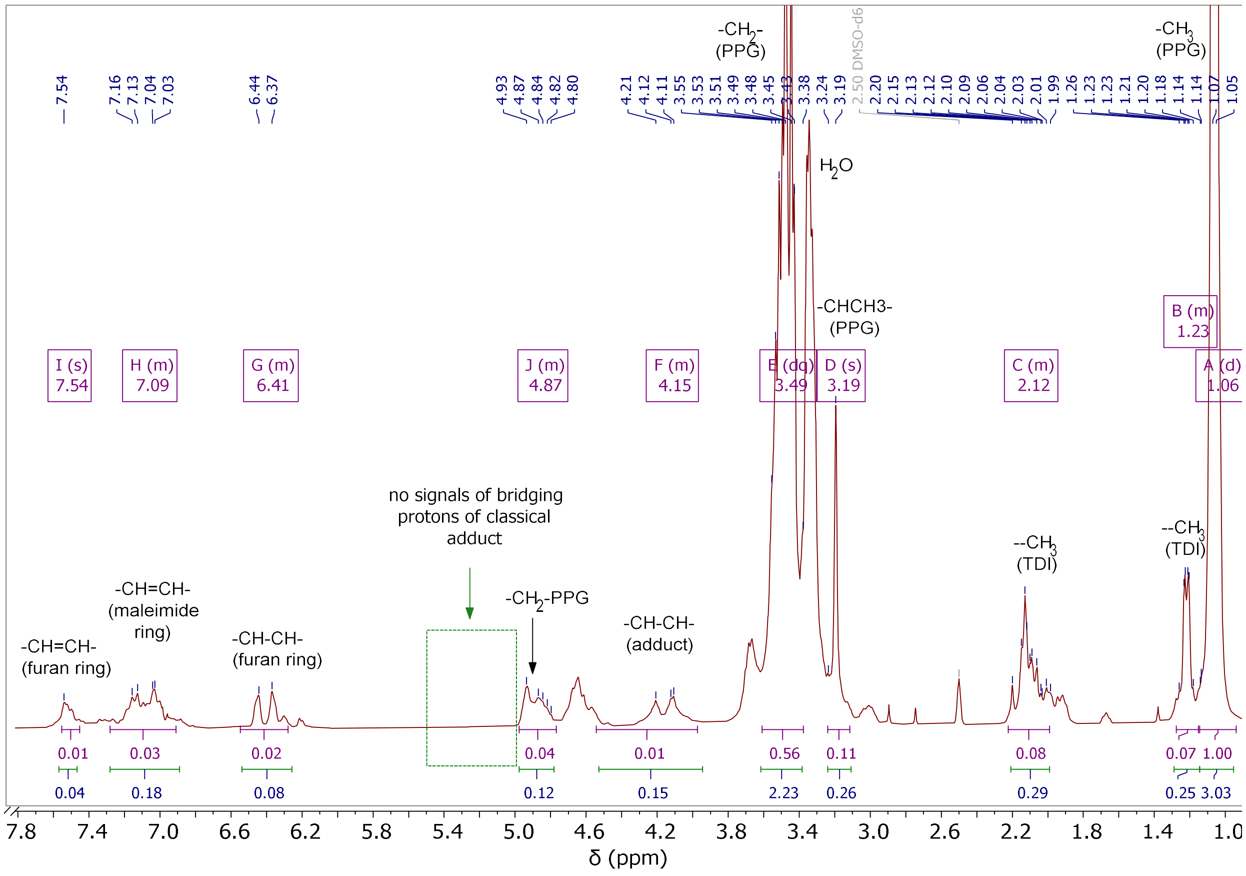

Figure S2i. ^{1}H NMR spectrum of the PU-T1 in DMSO-d_6.

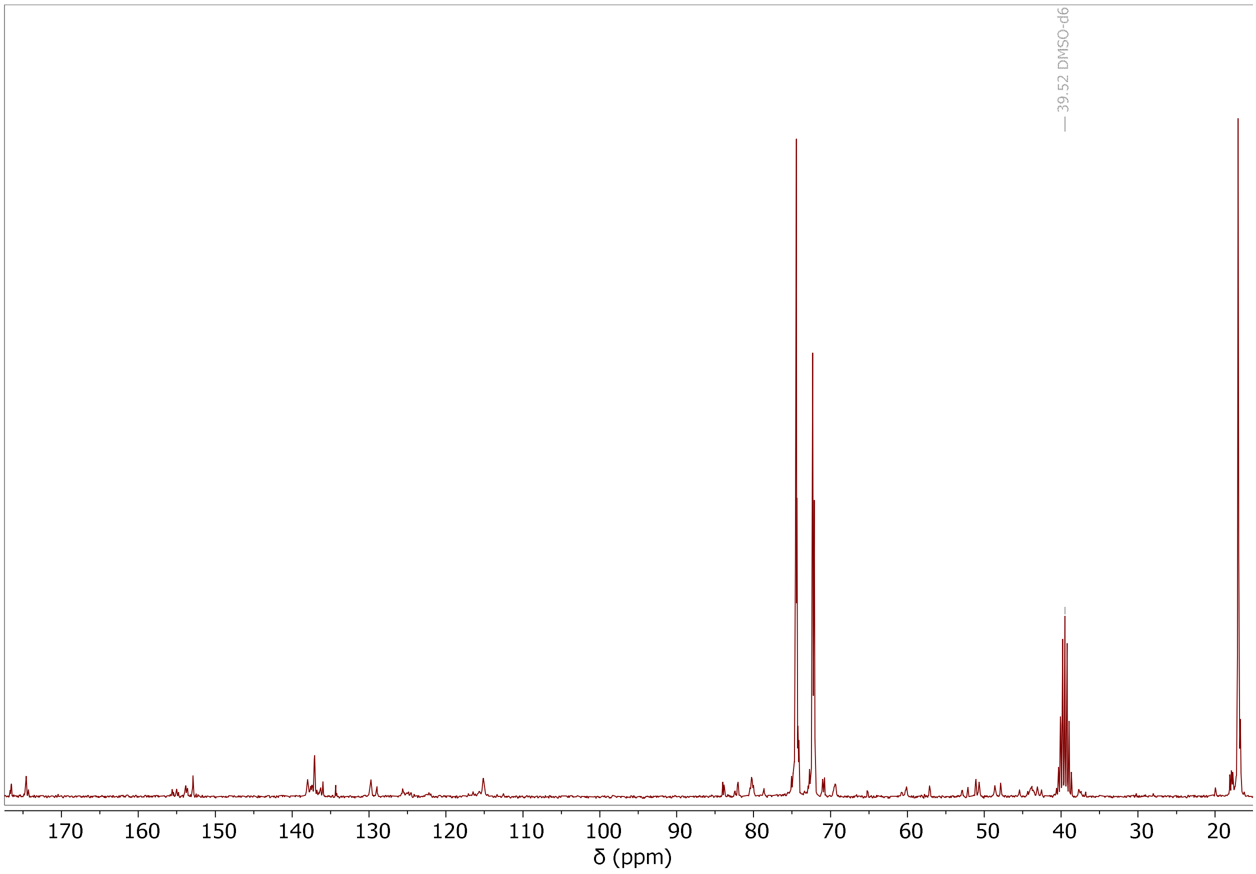

Figure S2j. ^{13}C NMR spectrum of the PU-T1 in DMSO-d_6.

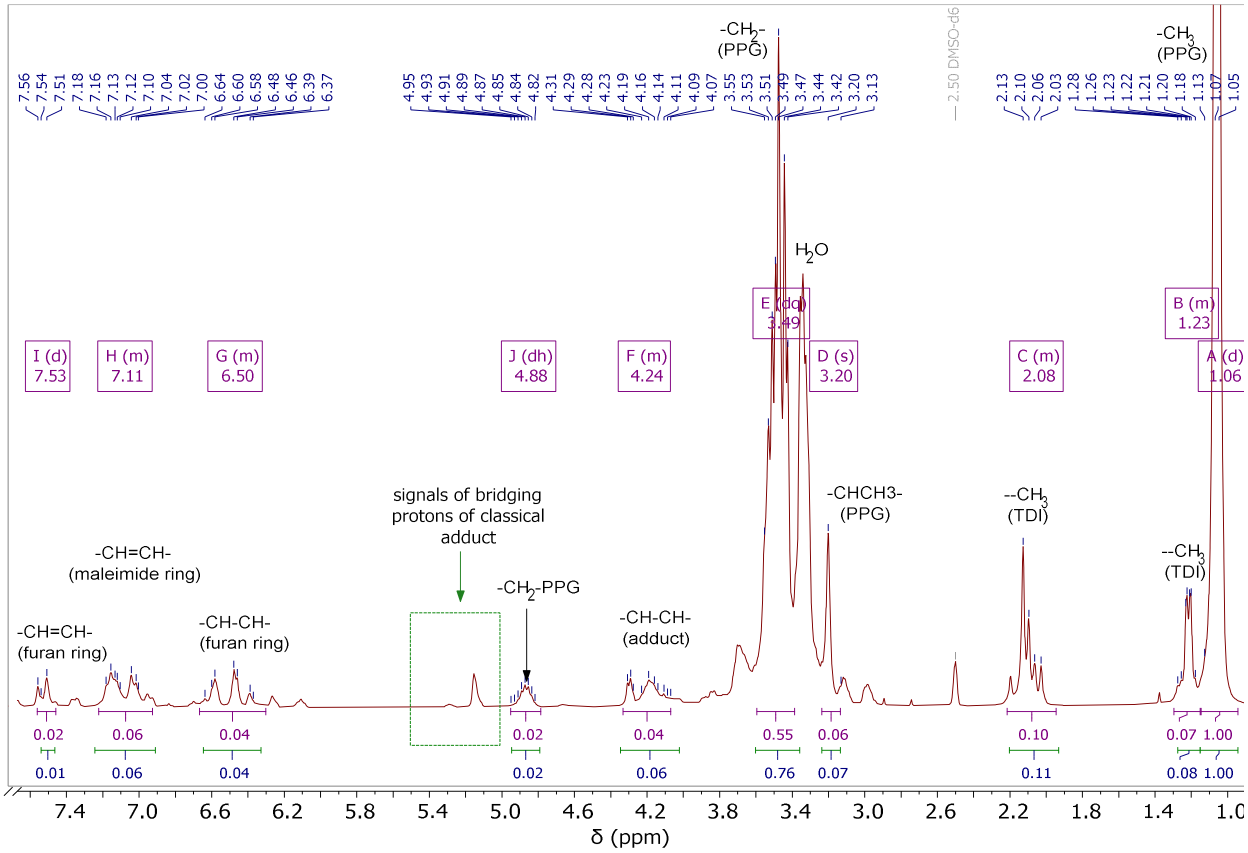

Figure S2k. ^{1}H NMR spectrum of the PU-T2 in DMSO-d_6.

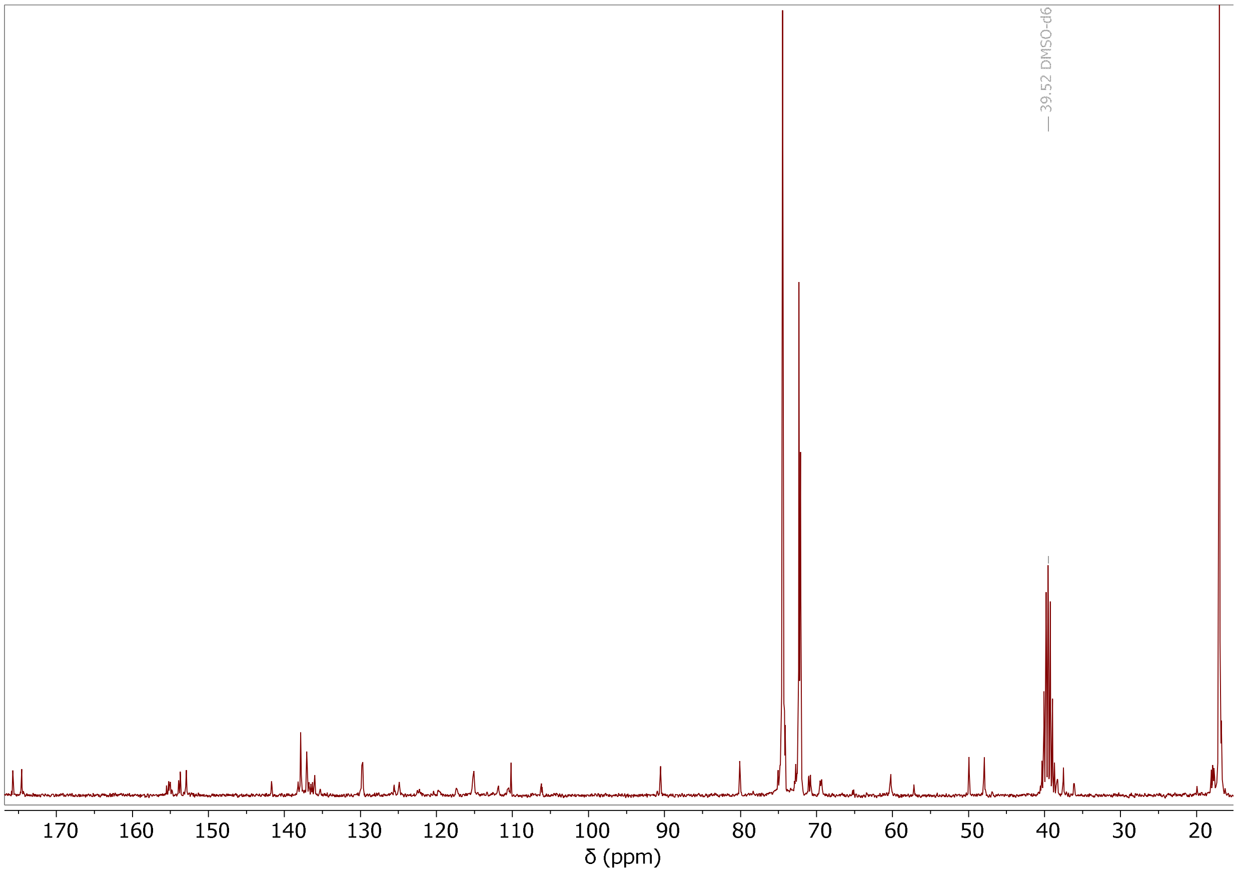

Figure S2l. ^{13}C NMR spectrum of the PU-T2 in DMSO-d_6.

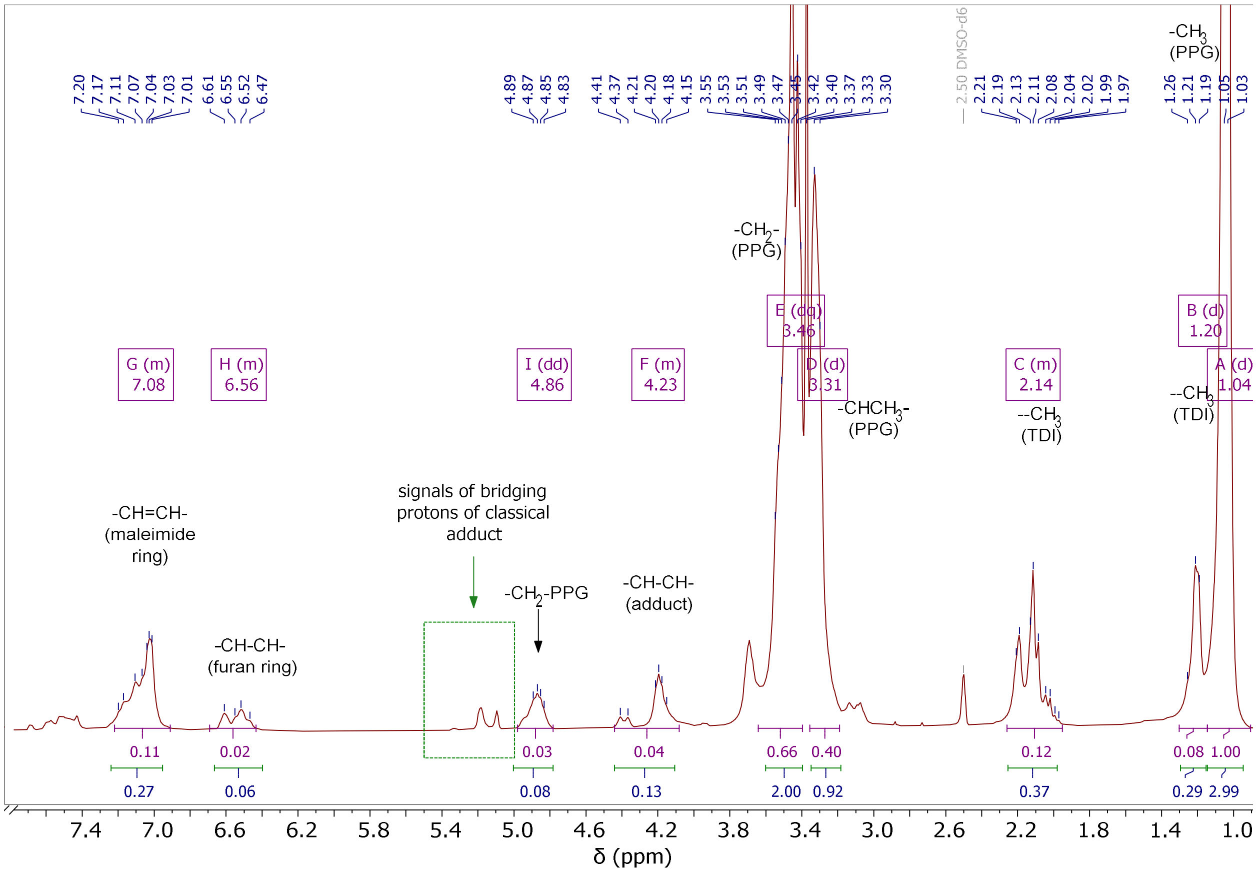

Figure S2m. ^{1}H NMR spectrum of the PU-T3 in DMSO-d_6.

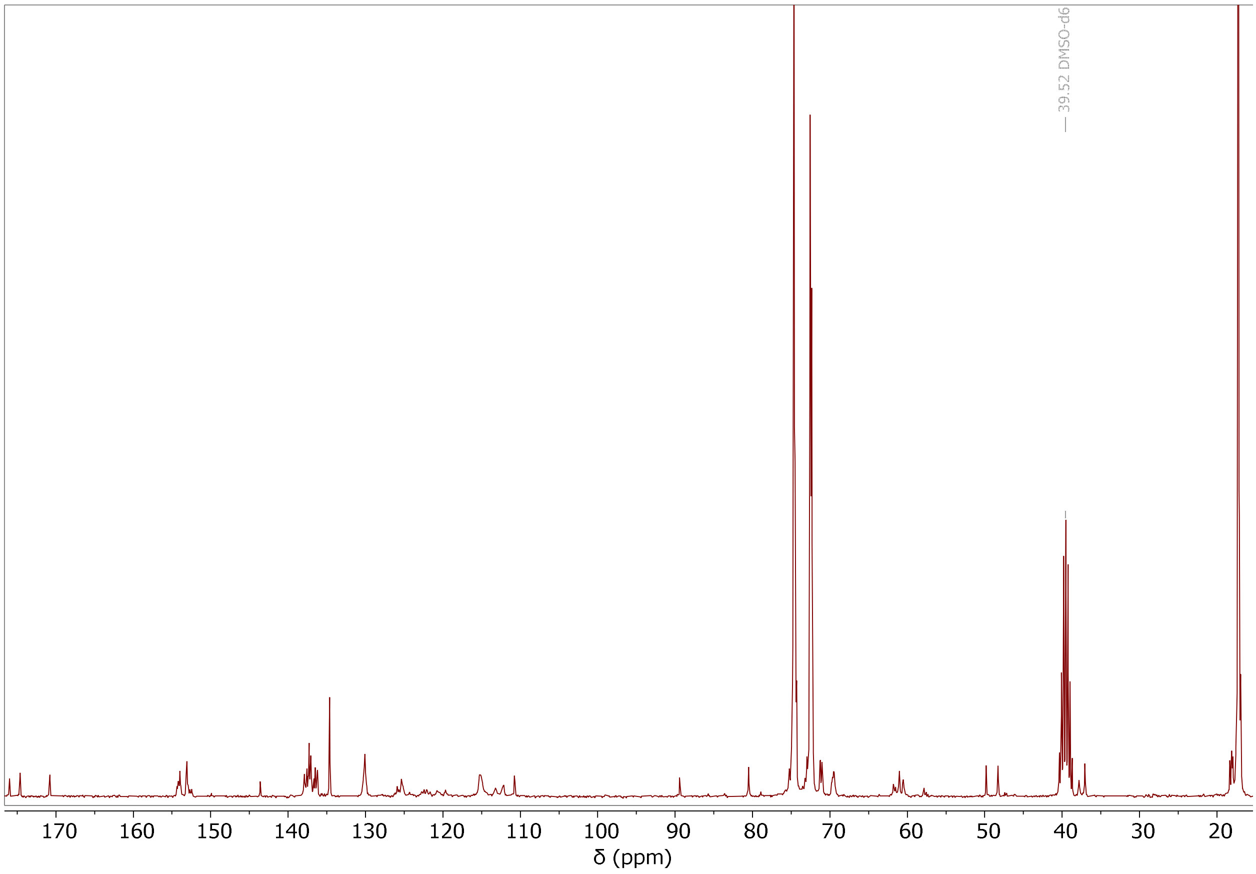

Figure S2n. ^{13}C NMR spectrum of the PU-T3 in DMSO-d_6.

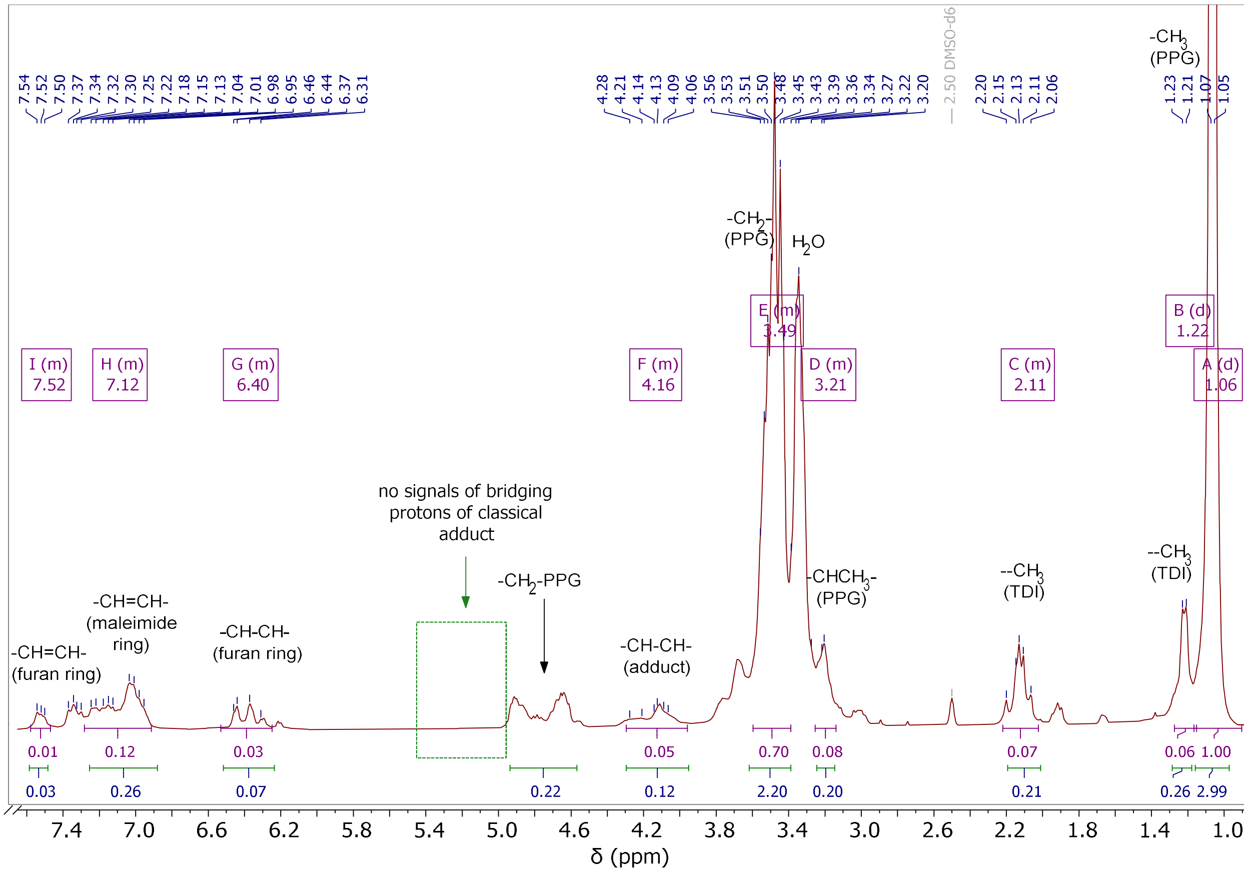

Figure S2o. ^{1}H NMR spectrum of the PU-M1 in DMSO-d_6.

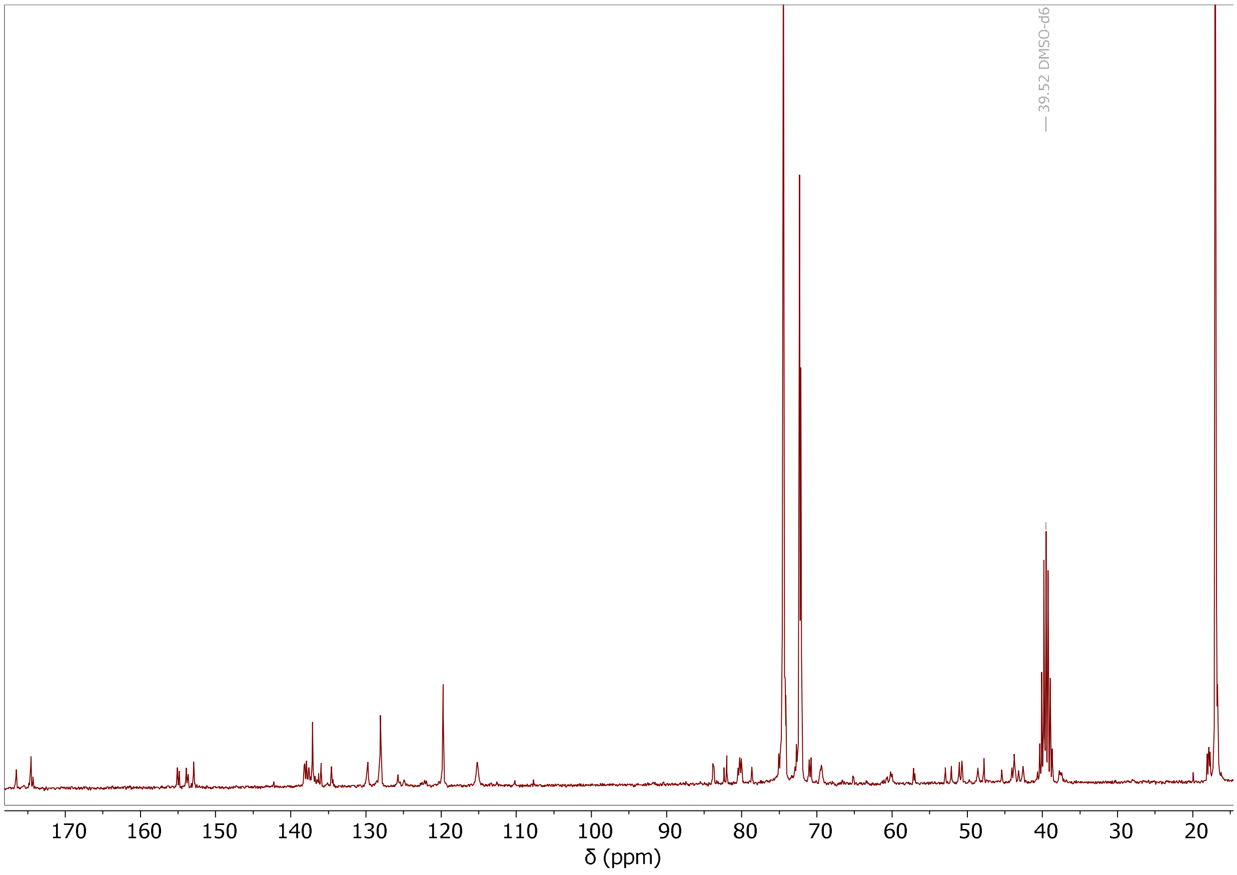

Figure S2p. ^{13}C NMR spectrum of the PU-M1 in DMSO-d_6.

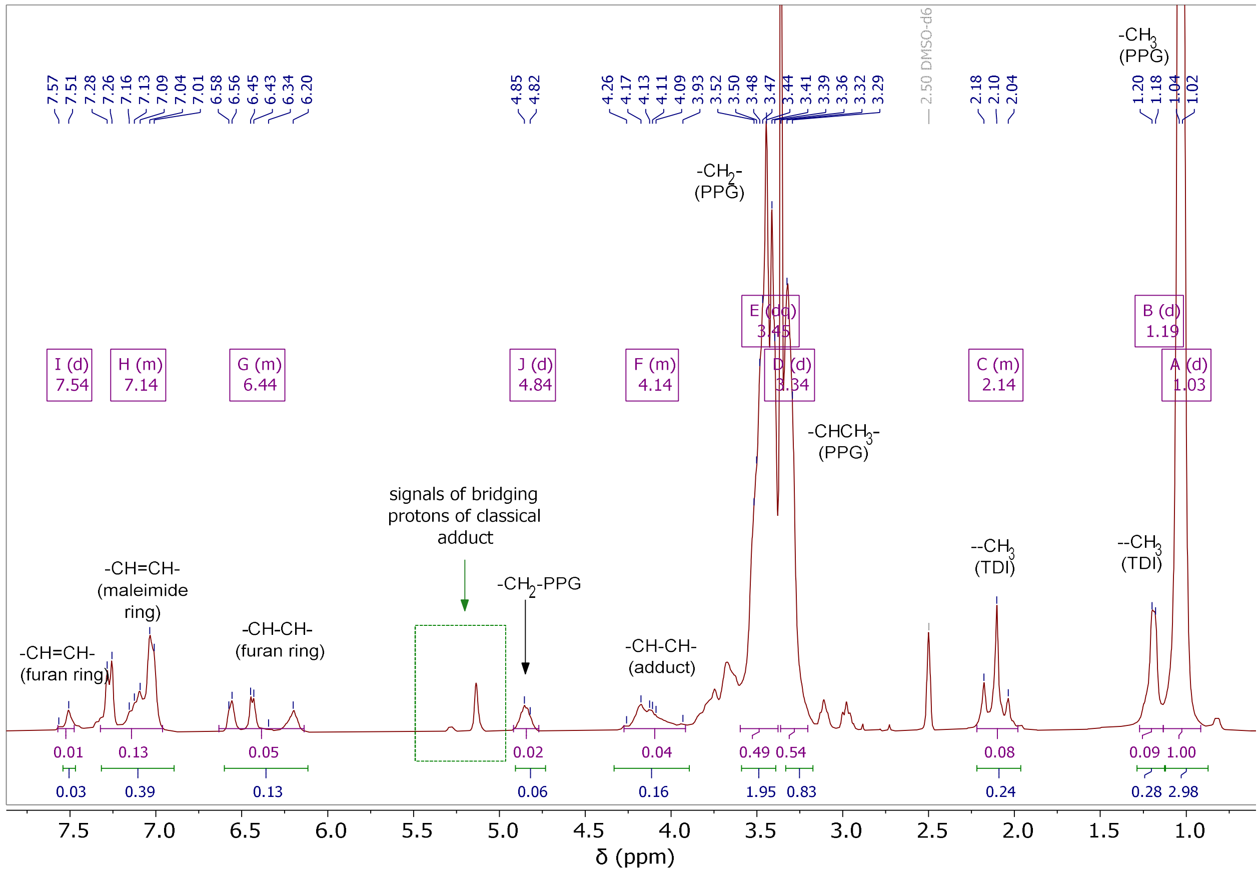

Figure S2q. ^{1}H NMR spectrum of the PU-M2 in DMSO-d_6.

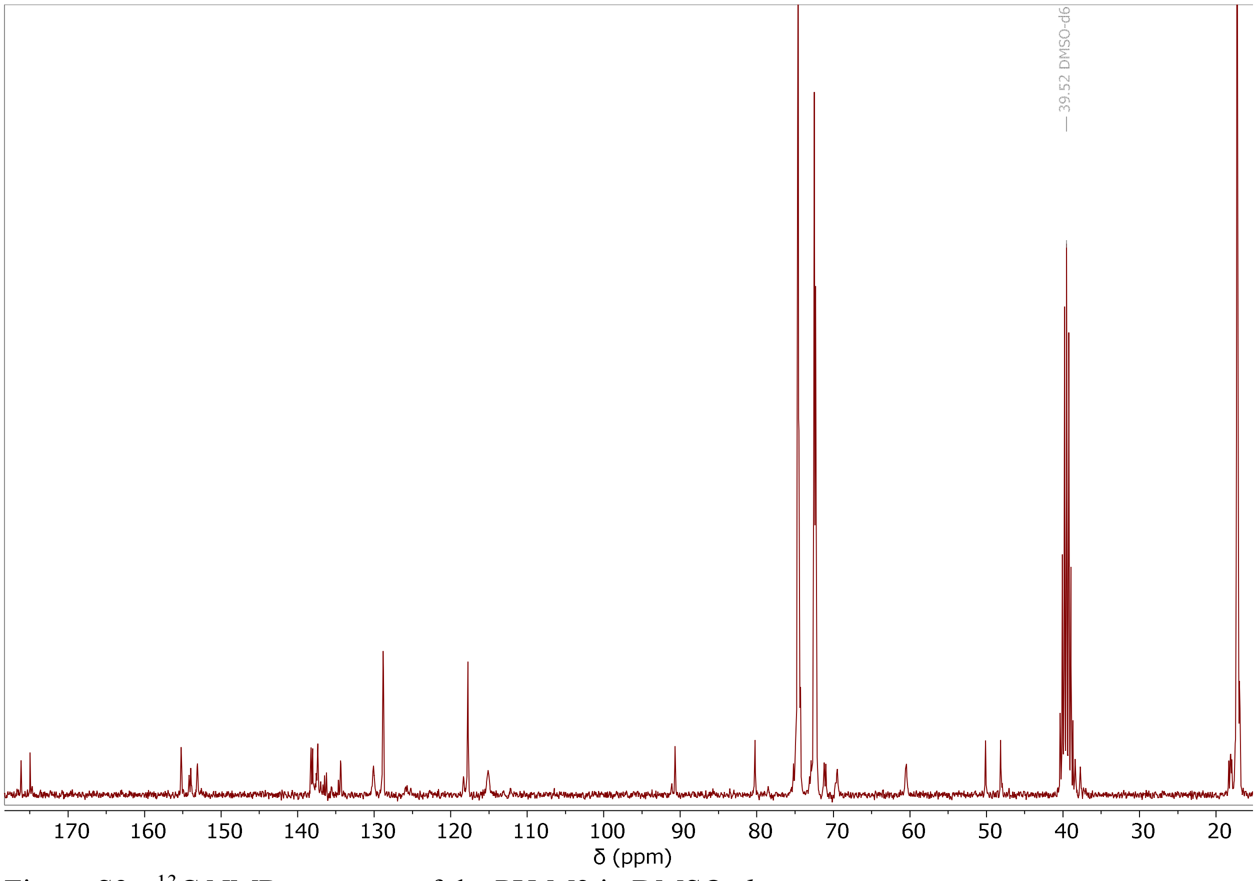

Figure S2r. ^{13}C NMR spectrum of the PU-M2 in DMSO-d_6.

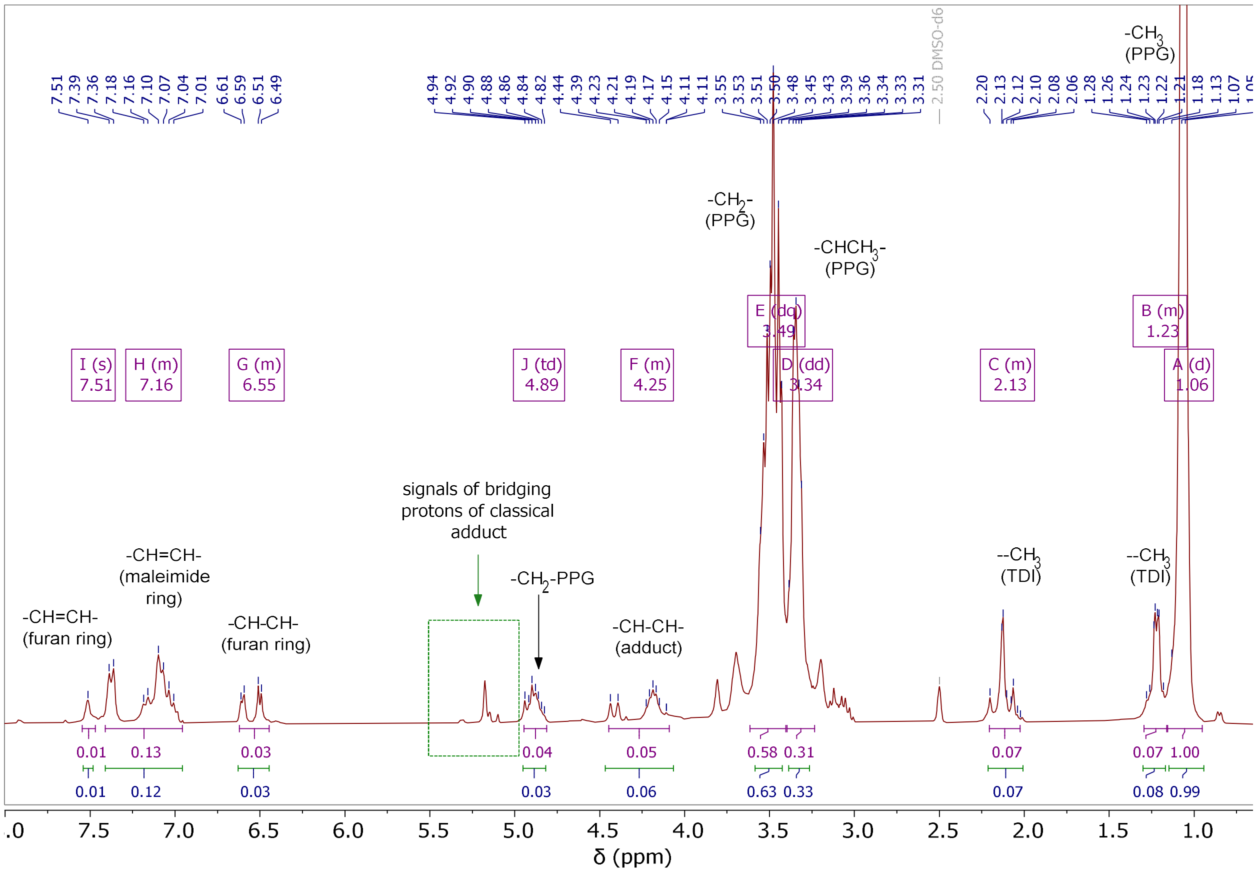

Figure S2s. ^{1}H NMR spectrum of the PU-M3 in DMSO-d_6.

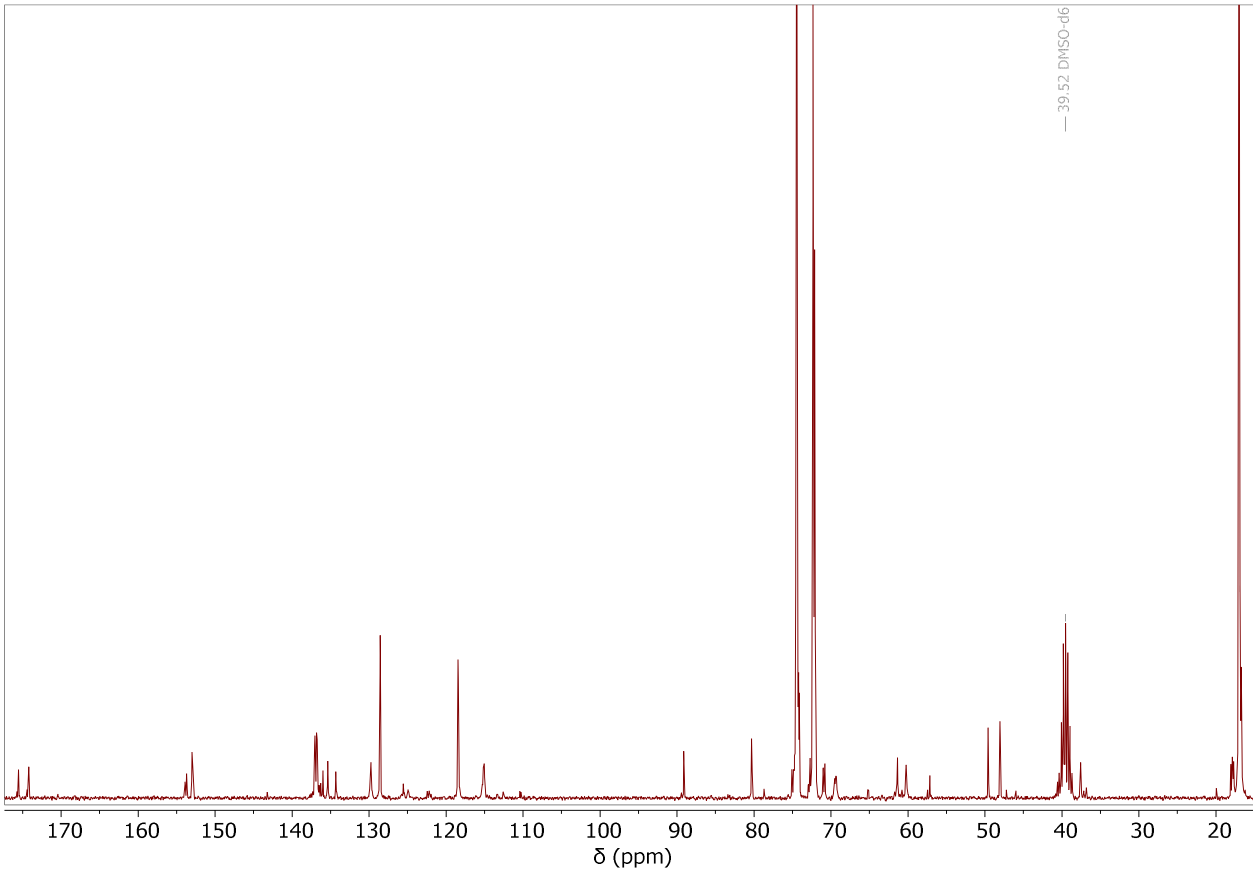

Figure S2t. ^{13}C NMR spectrum of the PU-M3 in DMSO-d_6.

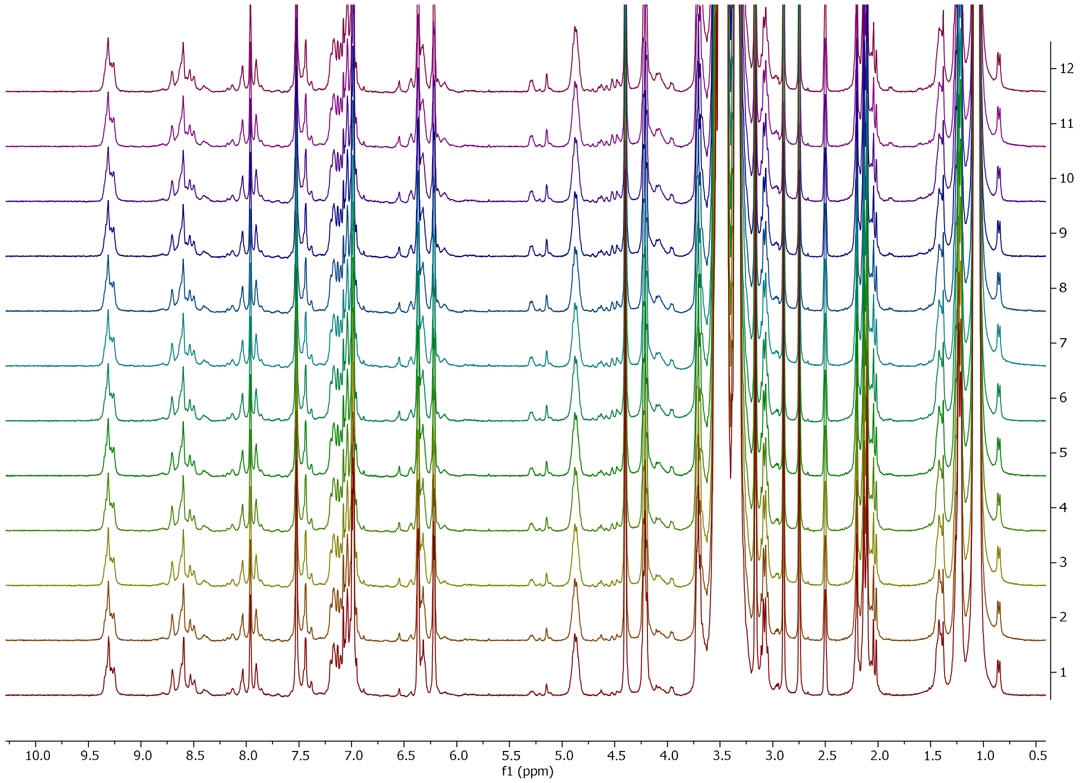

Figure S2u. Kinetic data for the polymerization reaction of PU-H1.

3 Attenuated Total Reflection-Fourier Transform Infrared Spectroscopy (ATR-FTIR)

ATI-FTIR was performed on Nicolet iS10 spectrometer in the range of the 4000 to 650 cm^{-1} on germanium crystal.

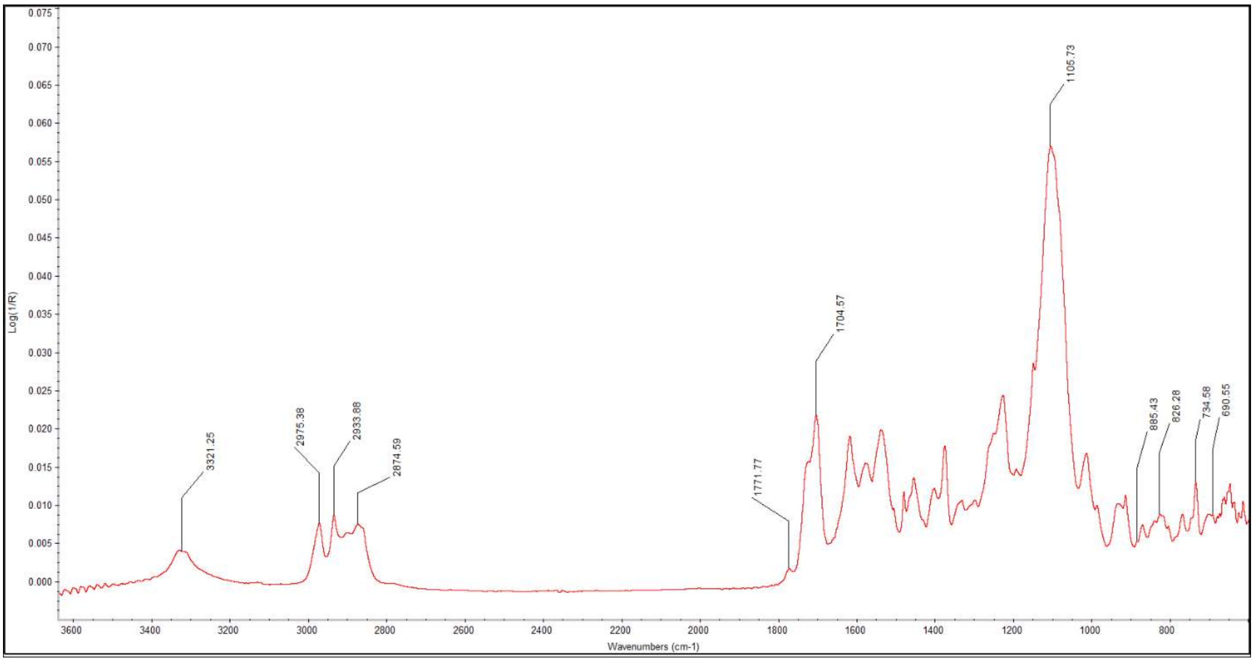

Figure S3a. ATI-FTIR spectrum of the PU-H1.

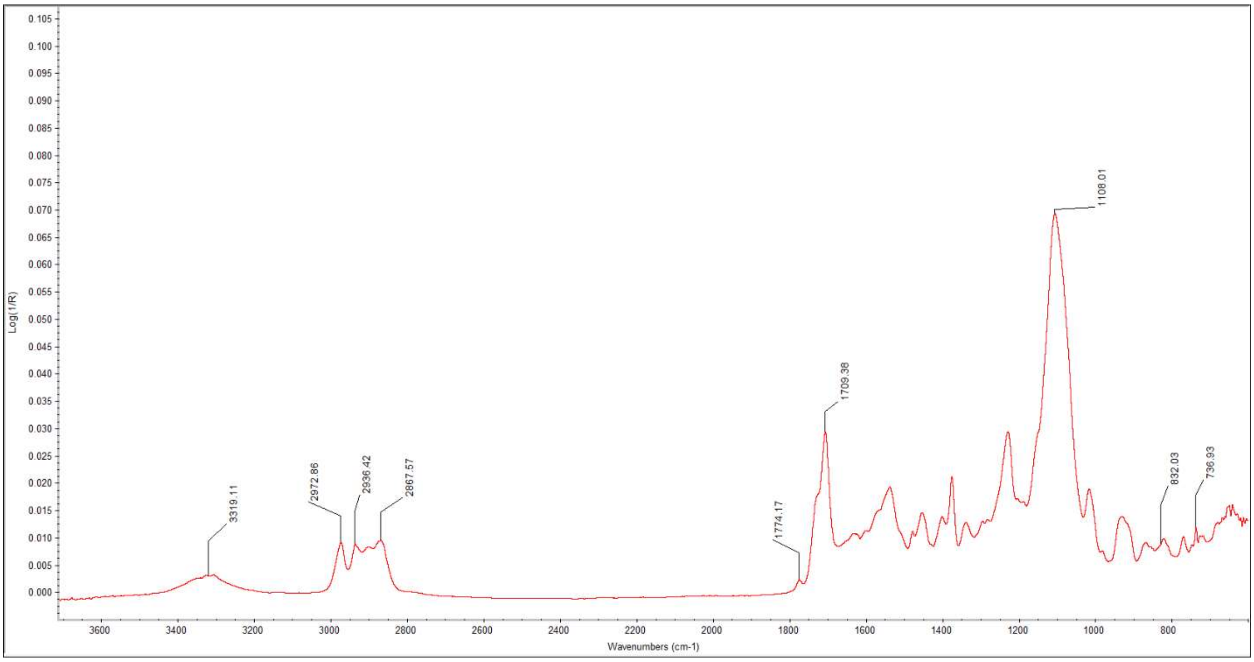

Figure S3b. ATI-FTIR spectrum of the PU-H2.

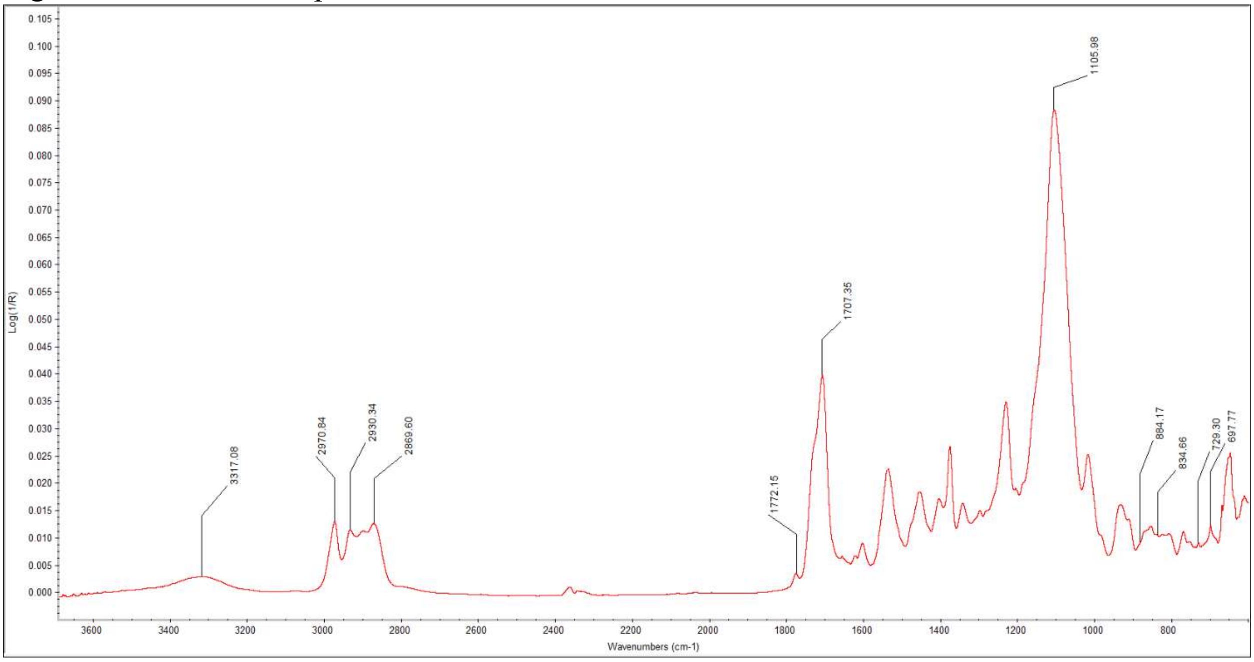

Figure S3c. ATI-FTIR spectrum of the PU-H3.

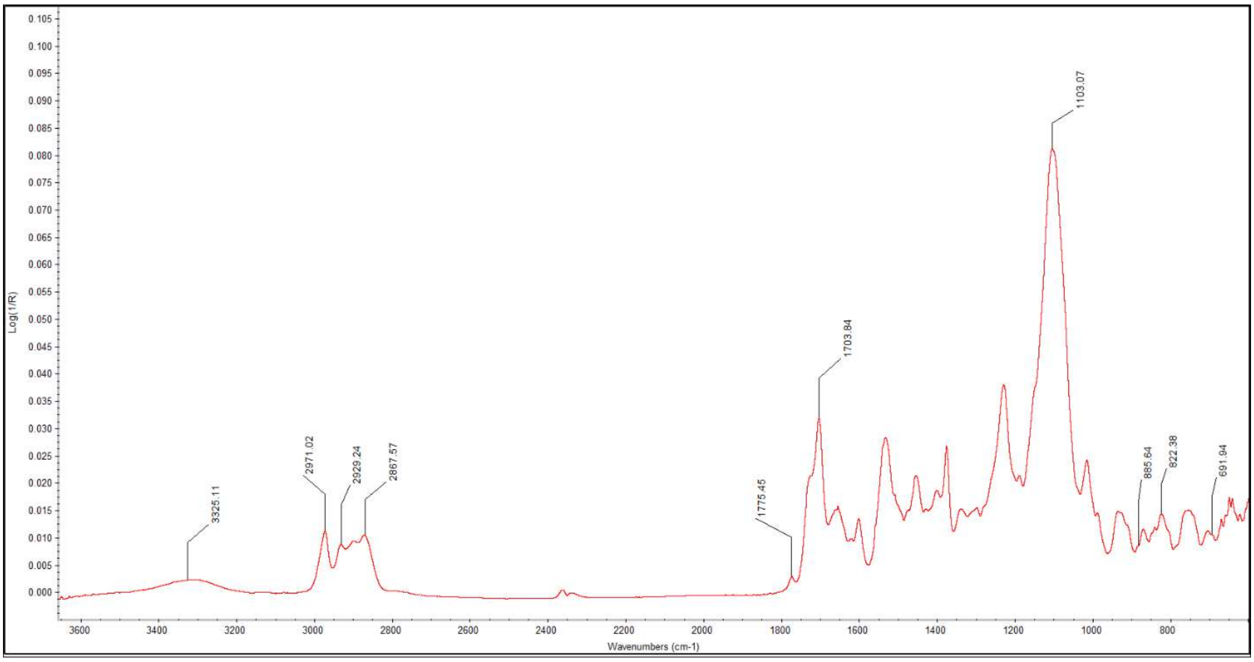

Figure S3d. ATI-FTIR spectrum of the PU-T1.

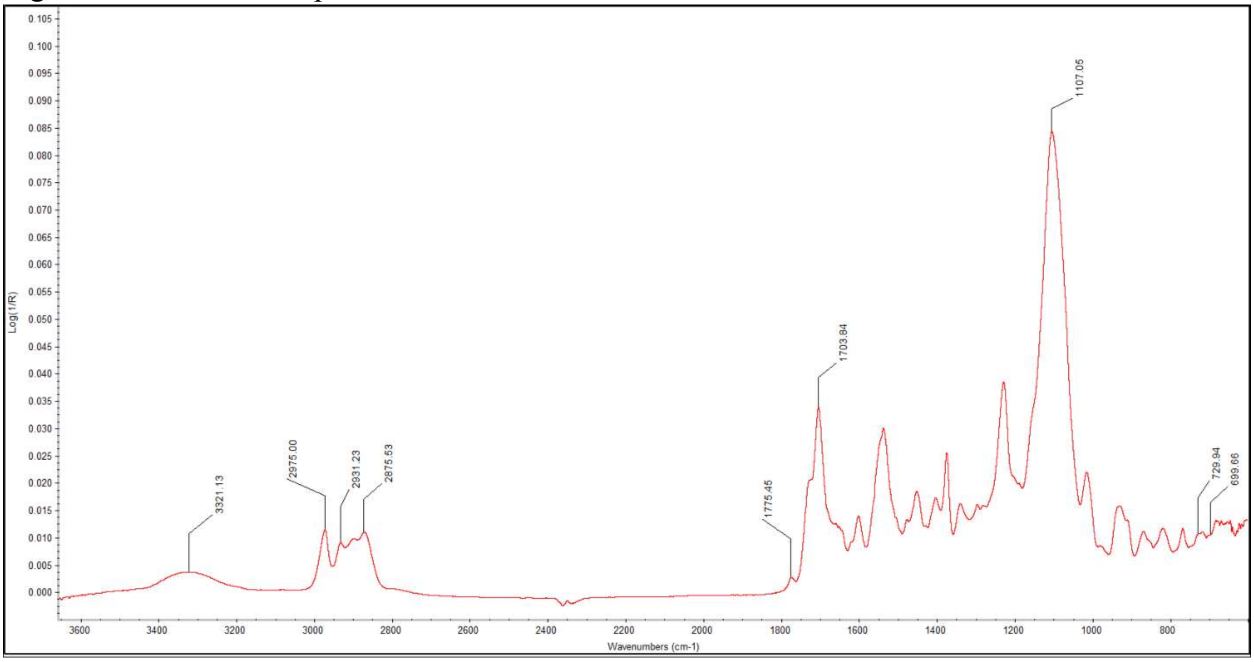

Figure S3e. ATI-FTIR spectrum of the PU-T2.

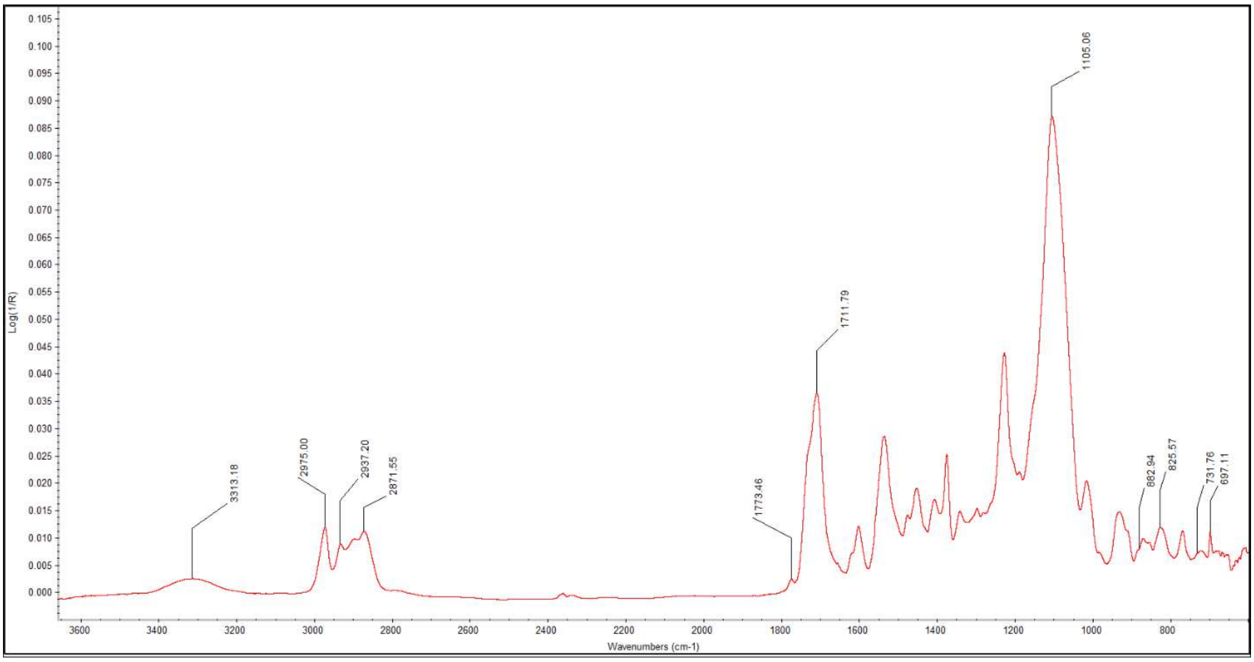

Figure S3f. ATI-FTIR spectrum of the PU-T3.

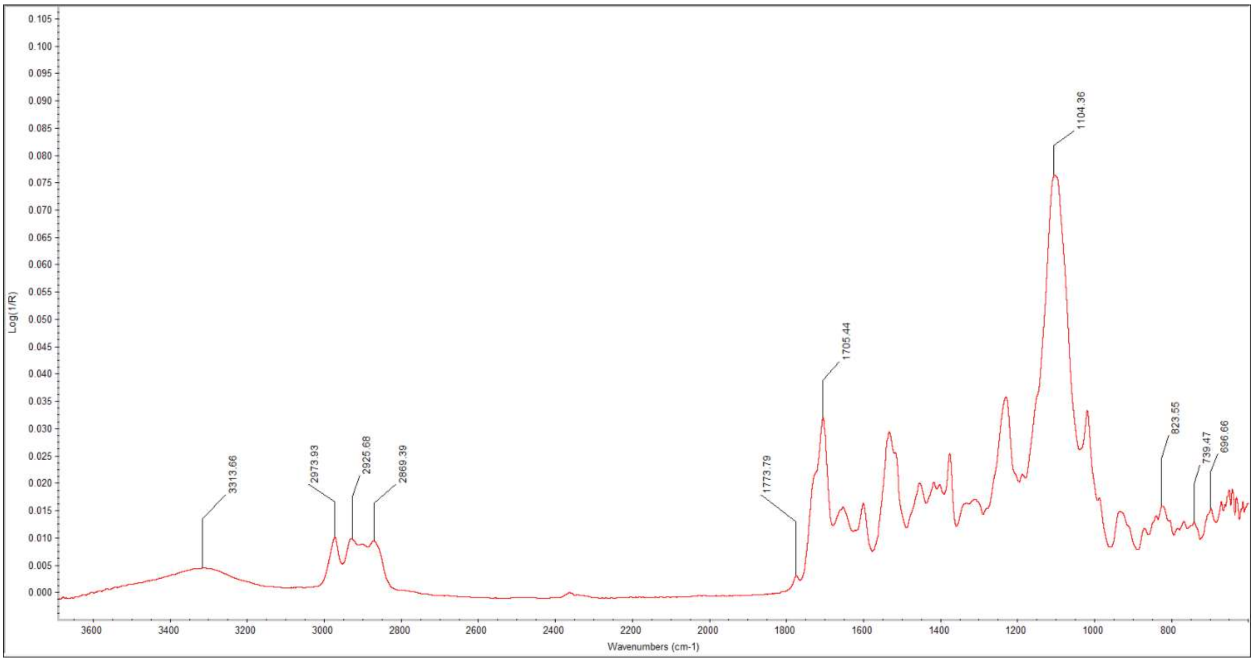

Figure S3g. ATI-FTIR spectrum of the PU-M1.

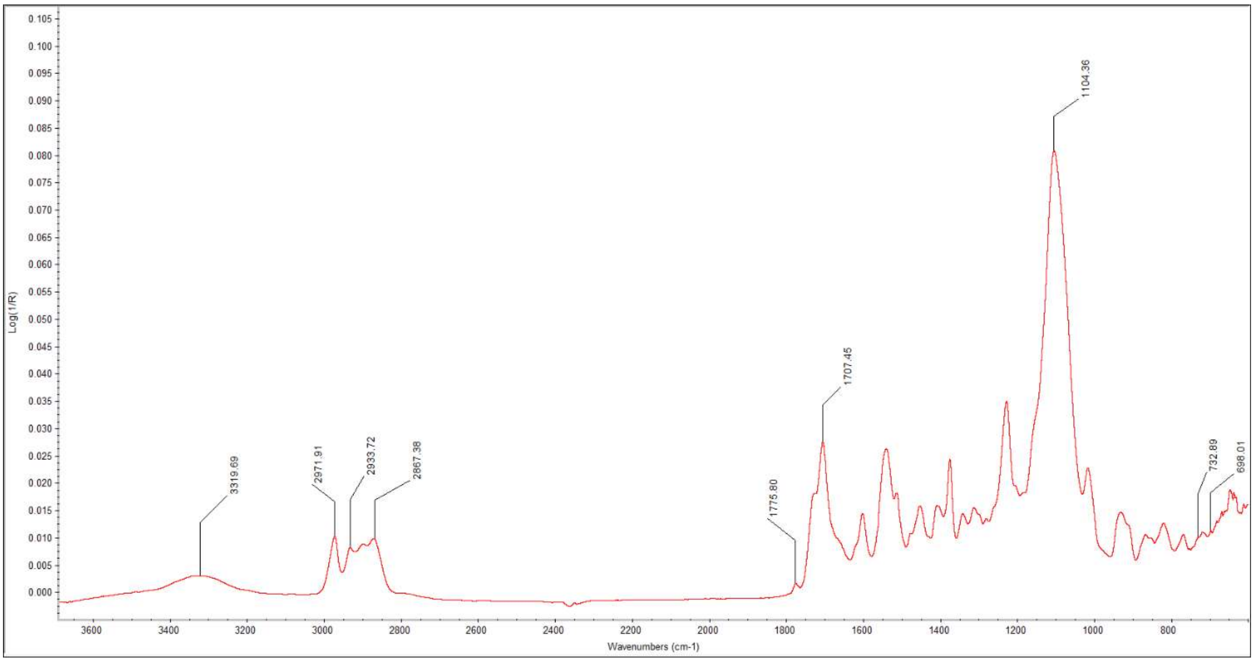

Figure S3h. ATI-FTIR spectrum of the PU-M2.

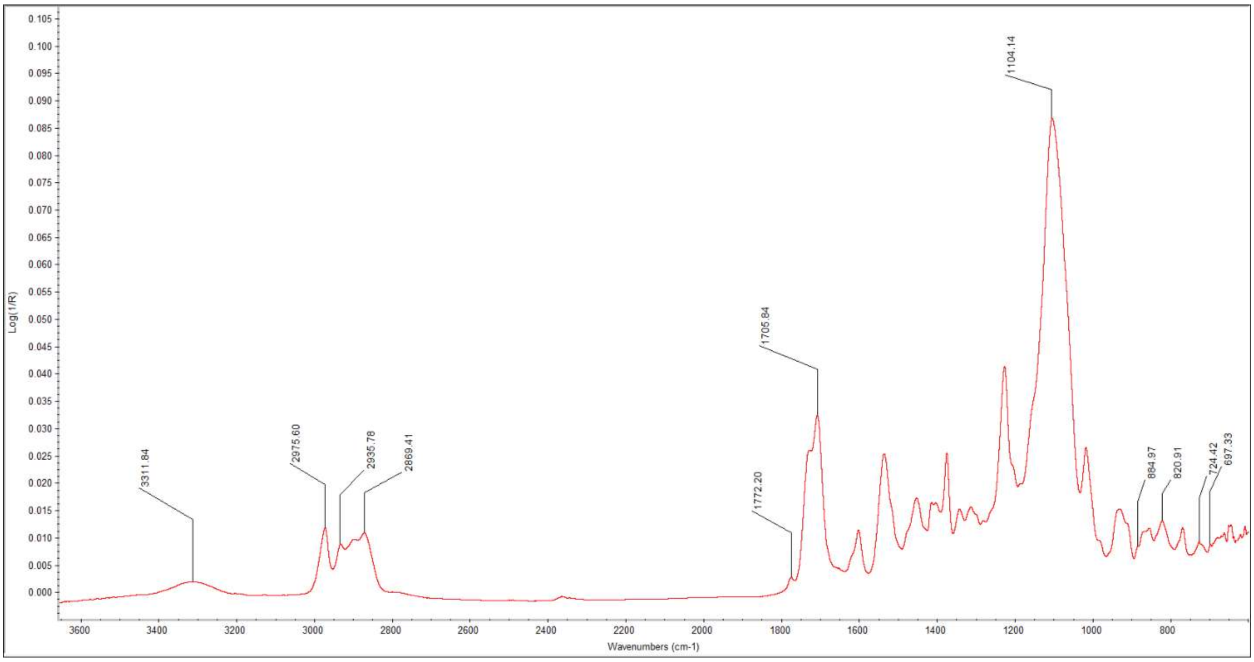

Figure S3i. ATI-FTIR spectrum of the PU-M3.

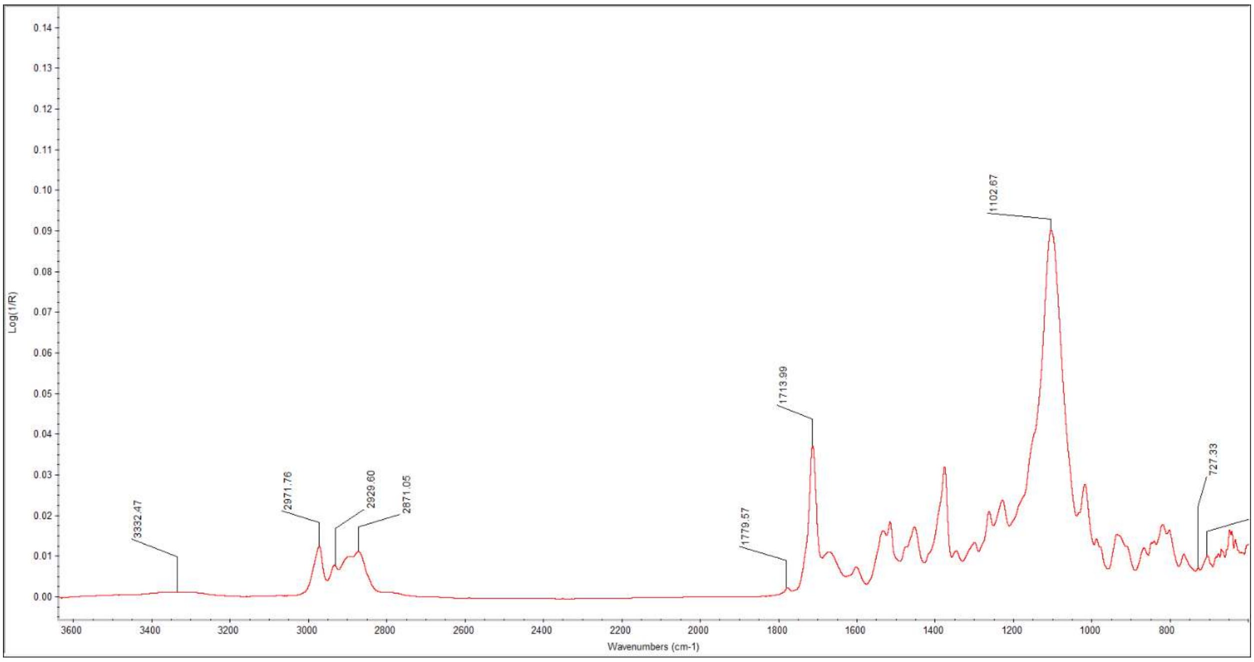

Figure S3j. ATI-FTIR spectrum of the PU-$(NFu_2)_2$.

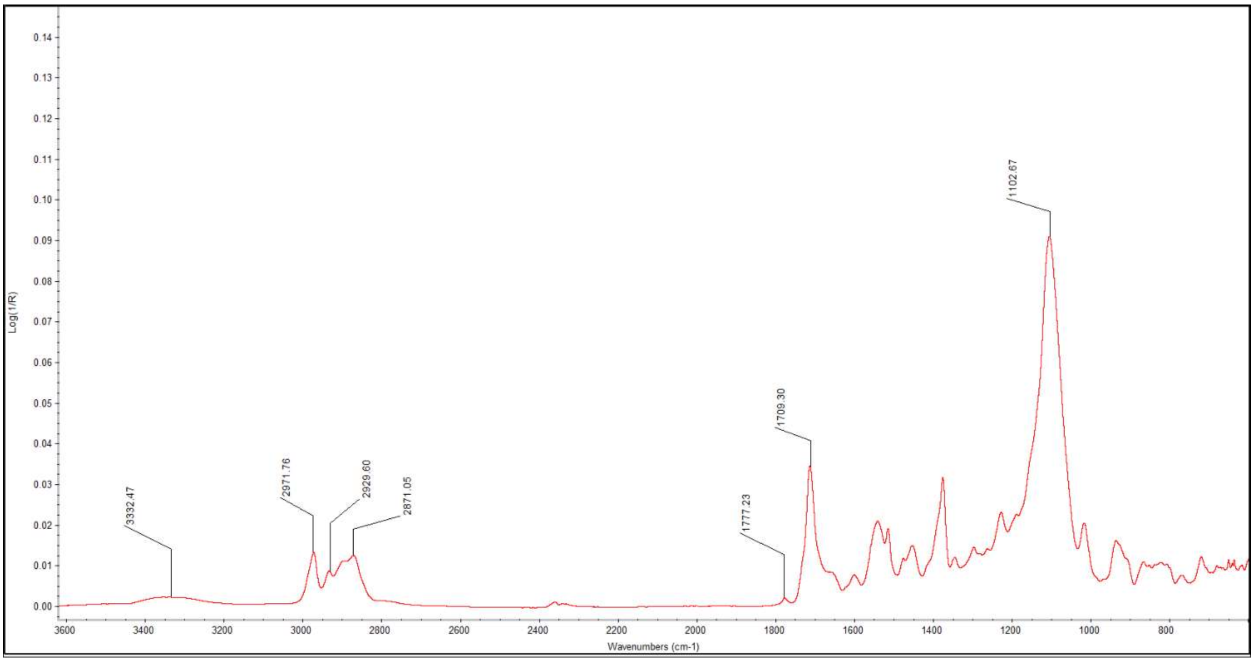

Figure S3k. ATI-FTIR spectrum of the PU-$(NFu)_2$.

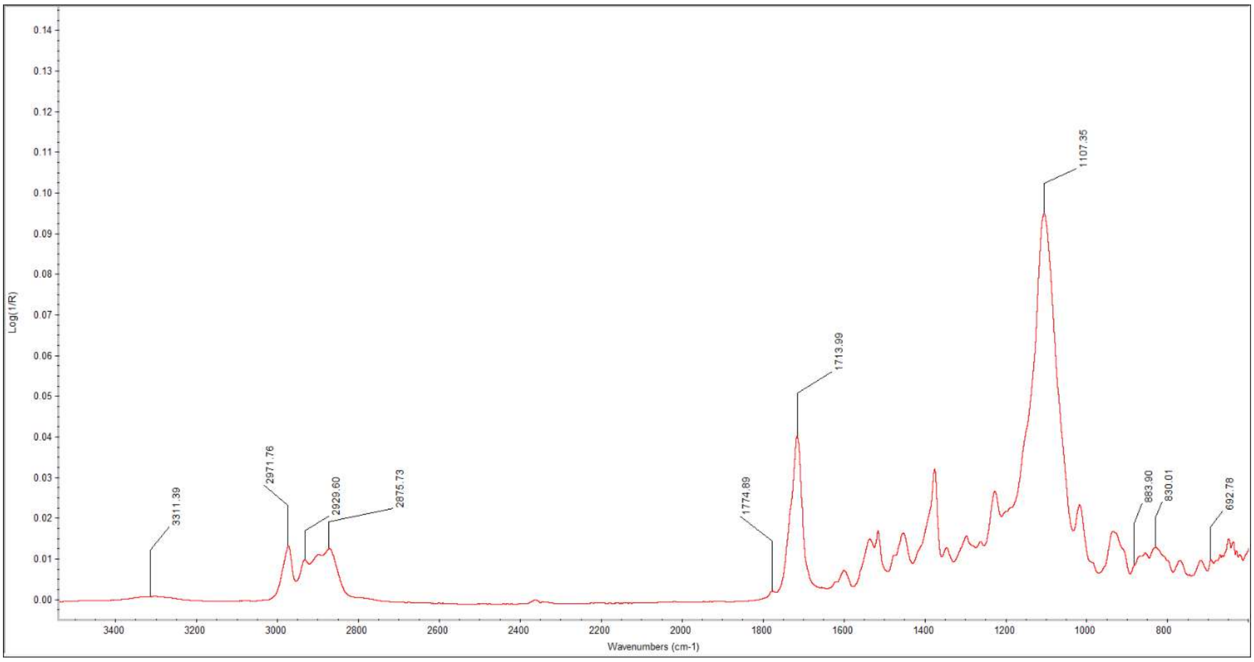

Figure S3l. ATI-FTIR spectrum of the PU-$(OFu)_2$.

4 Thermogravimetric Analysis (TGA)

TGA was performed on NETZSCH TG 209 F1 Libra within a temperature range of the 30 to 550 °C at heating/cooling rate of the 10 K min-1 in an argon atmosphere.

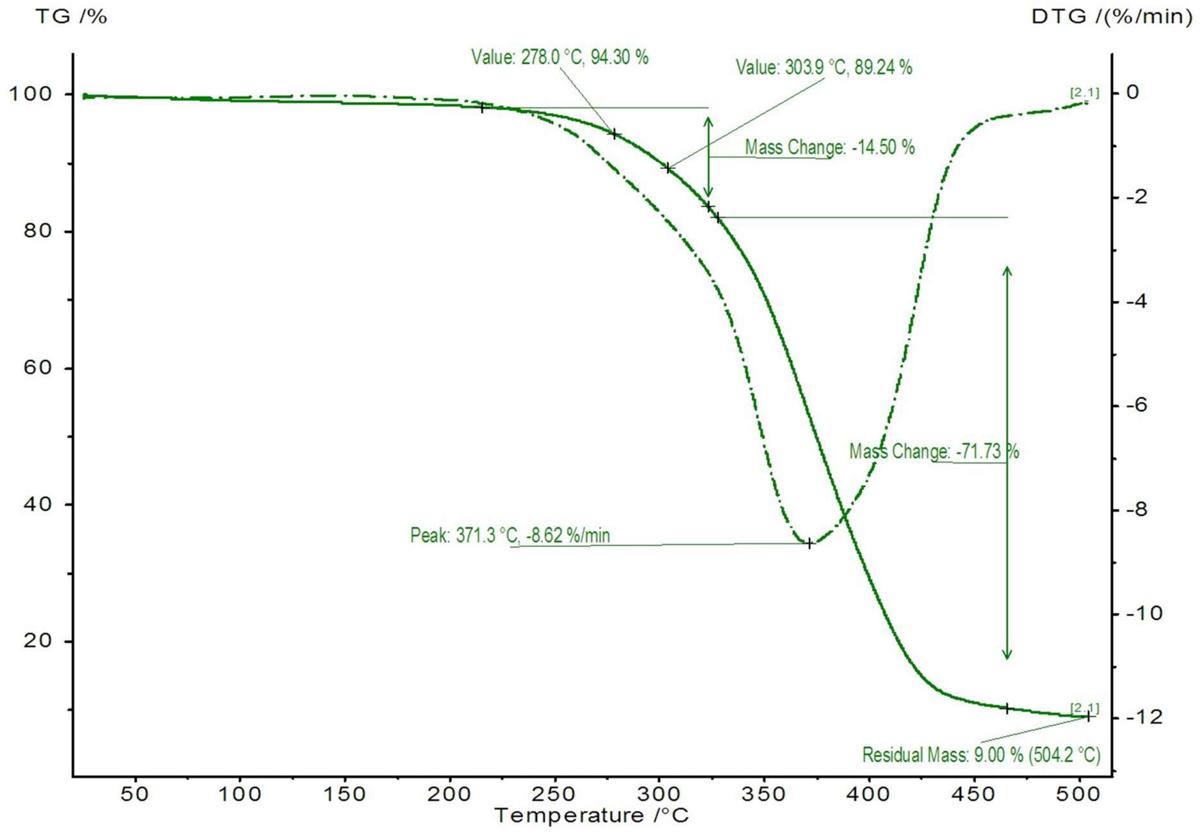

Figure S4a. TGA and DTG curves of the PU-H1.

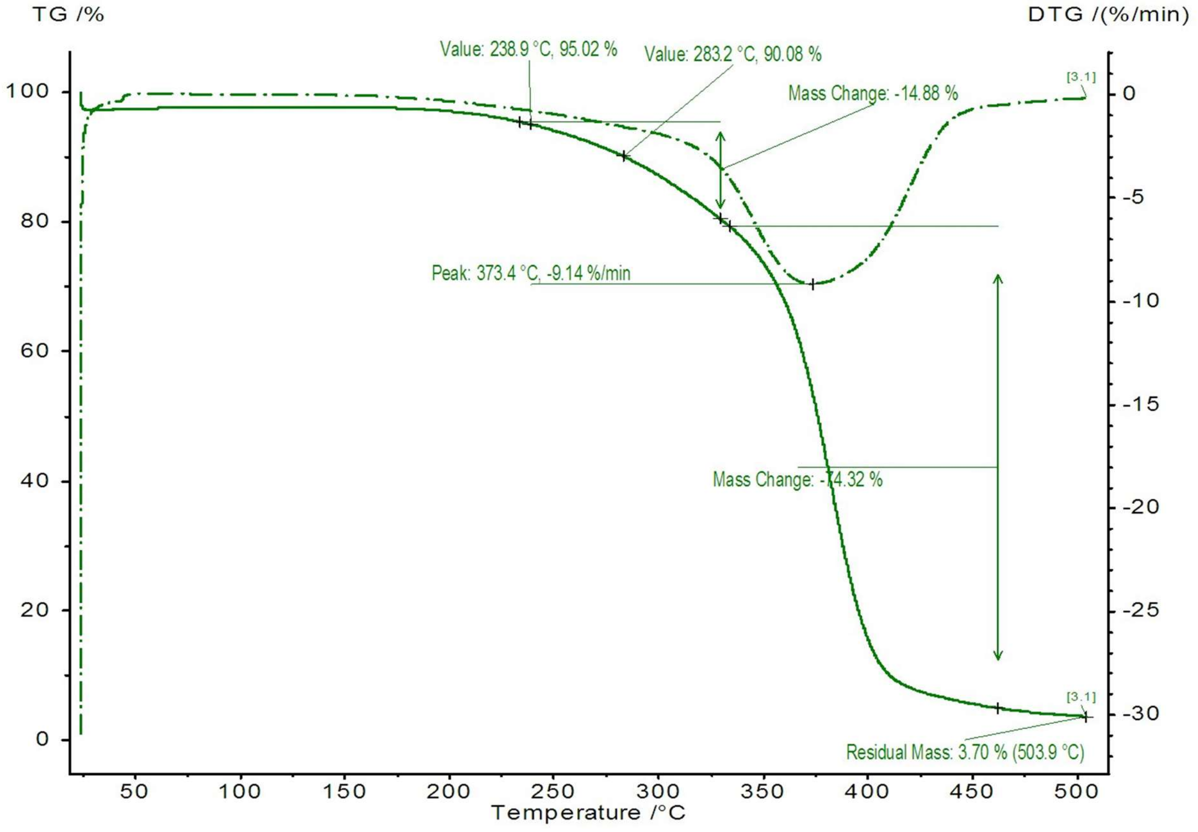

Figure S4b. TGA and DTG curves of the PU-H2.

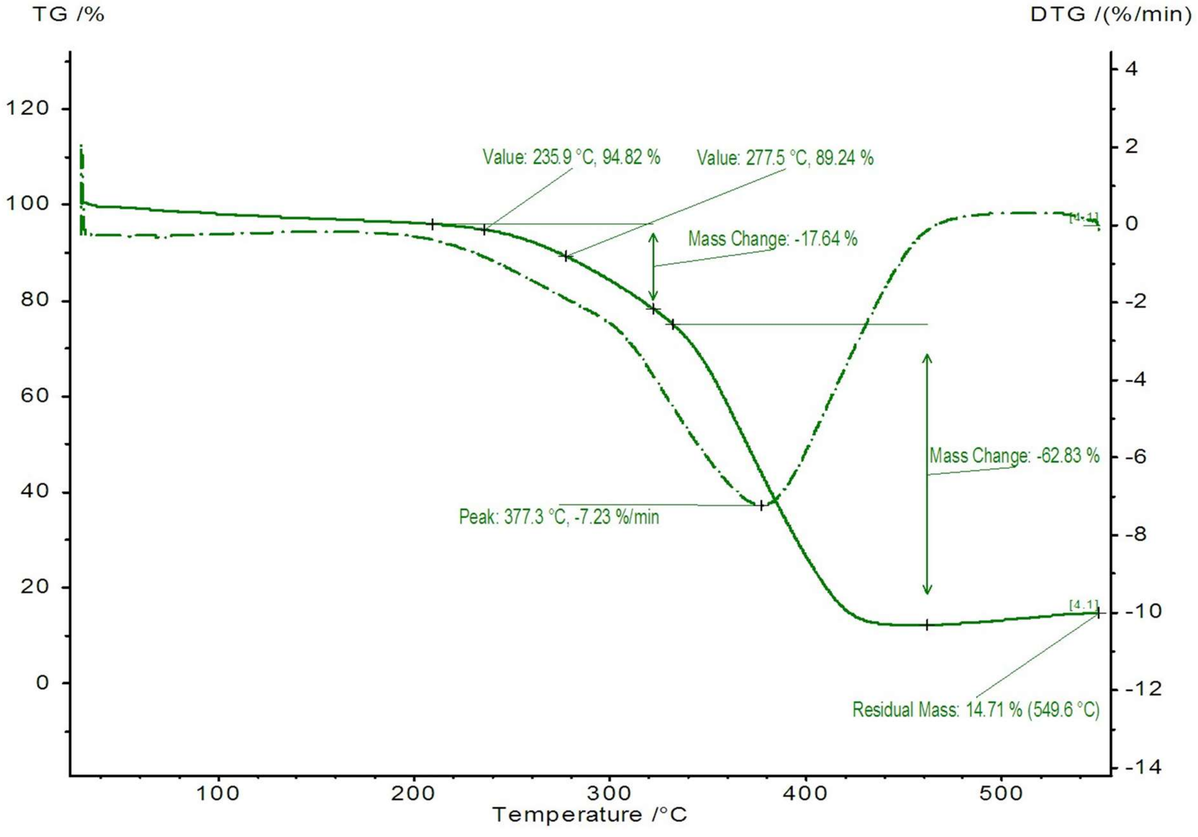

Figure S4c. TGA and DTG curves of the PU-H3.

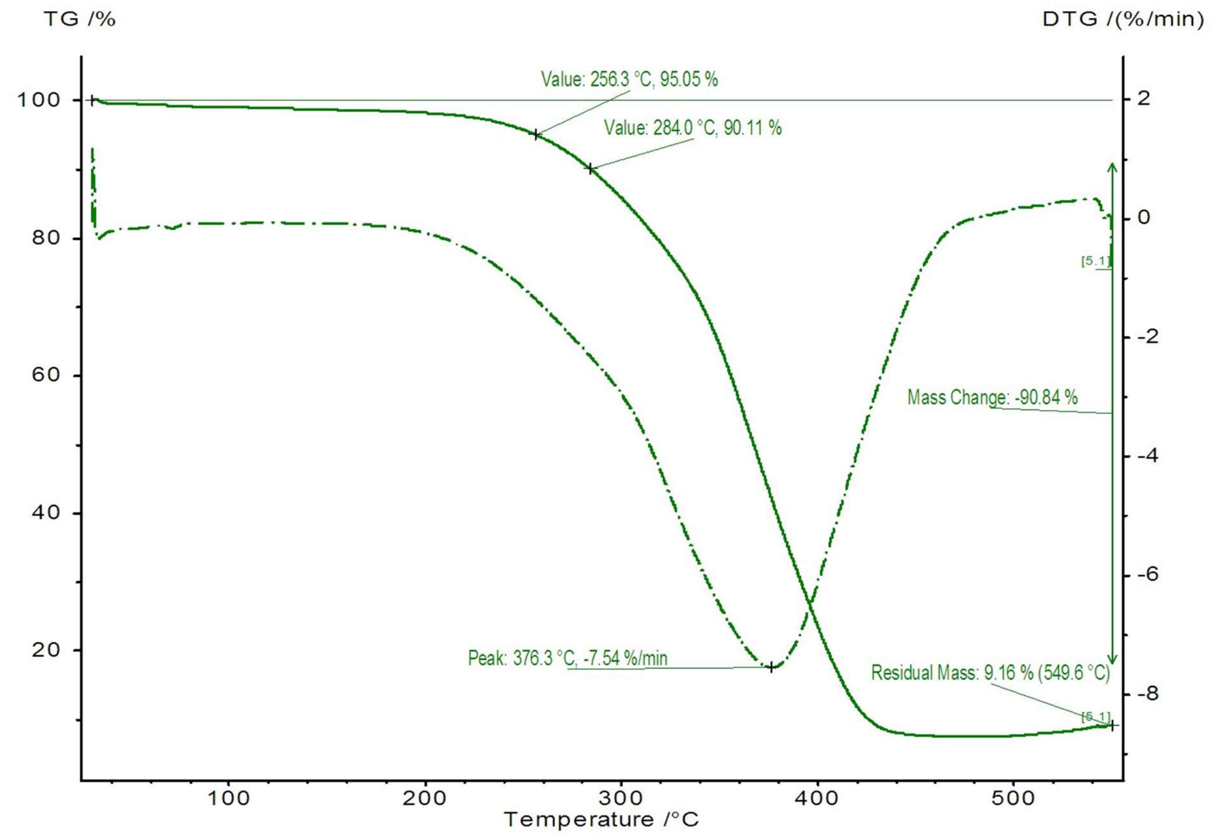

Figure S4d. TGA and DTG curves of the PU-T1.

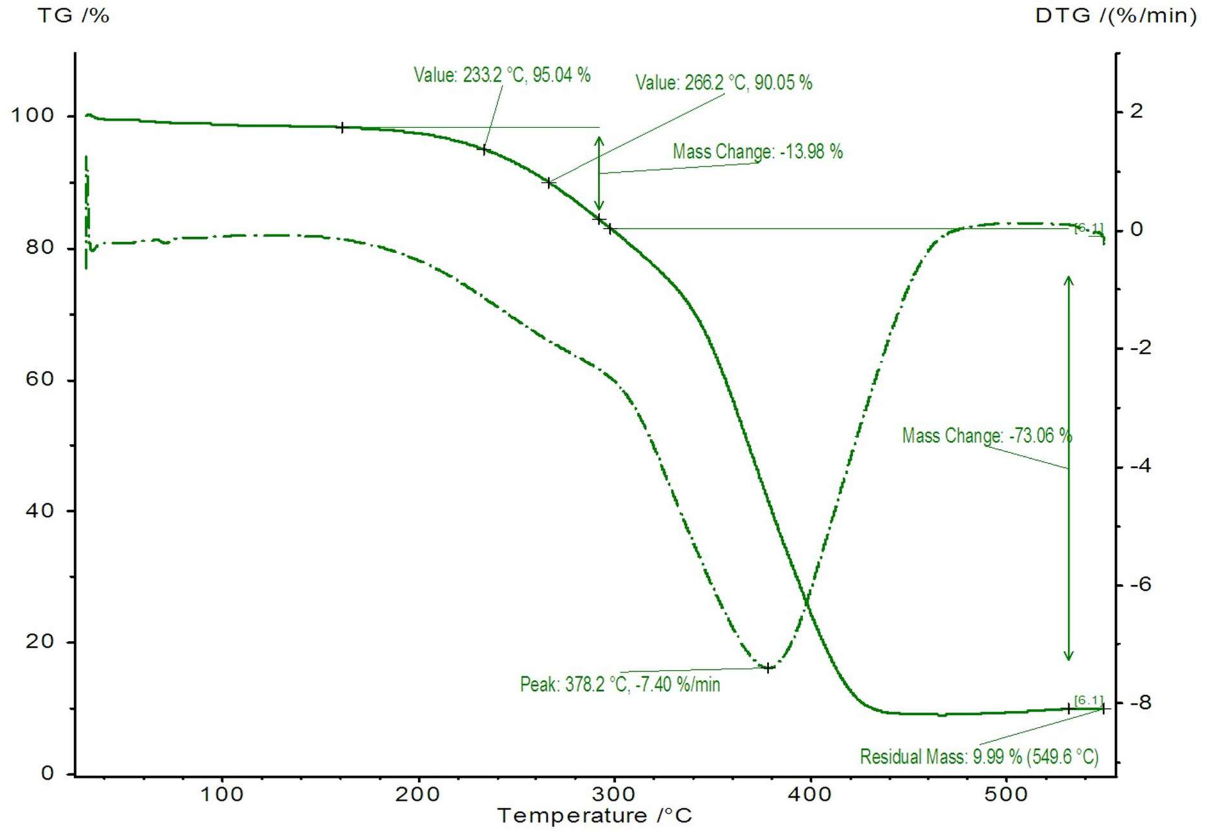

Figure S4e. TGA and DTG curves of the PU-T2.

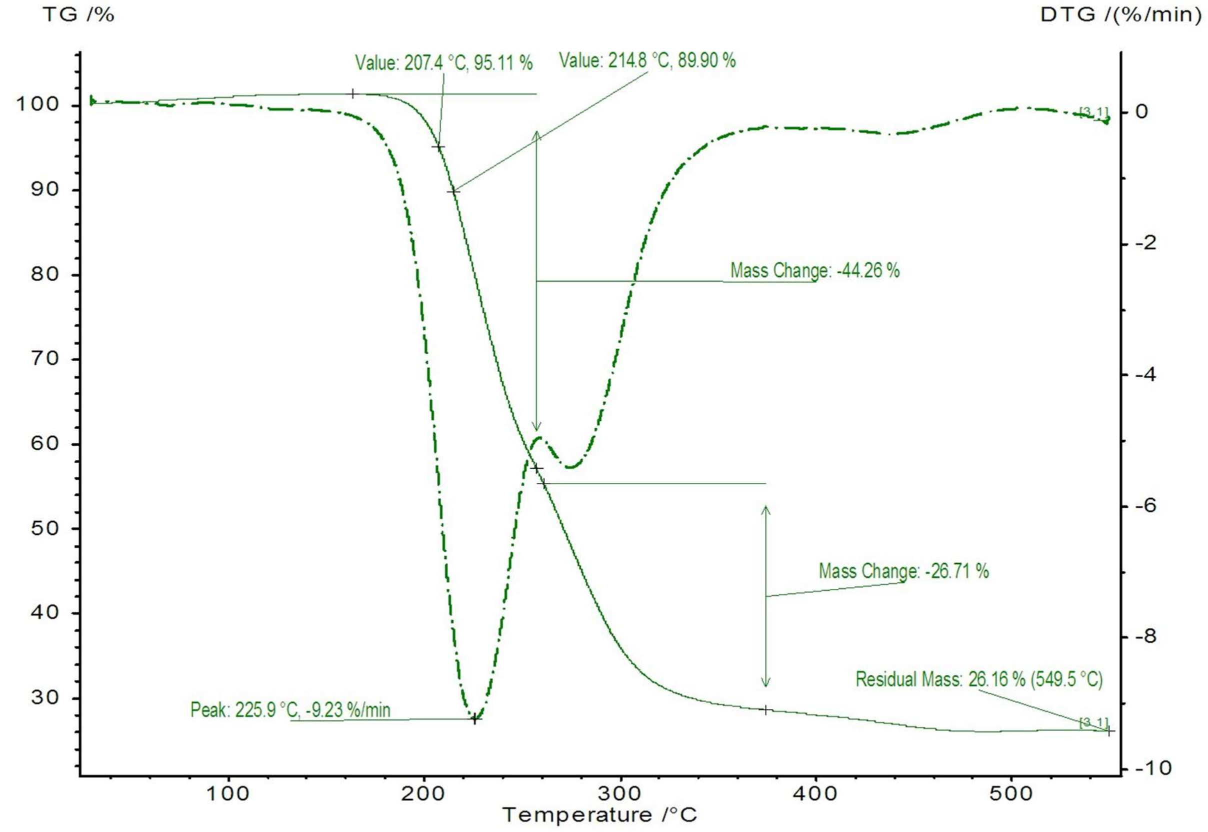

Figure S4f. TGA and DTG curves of the PU-T3.

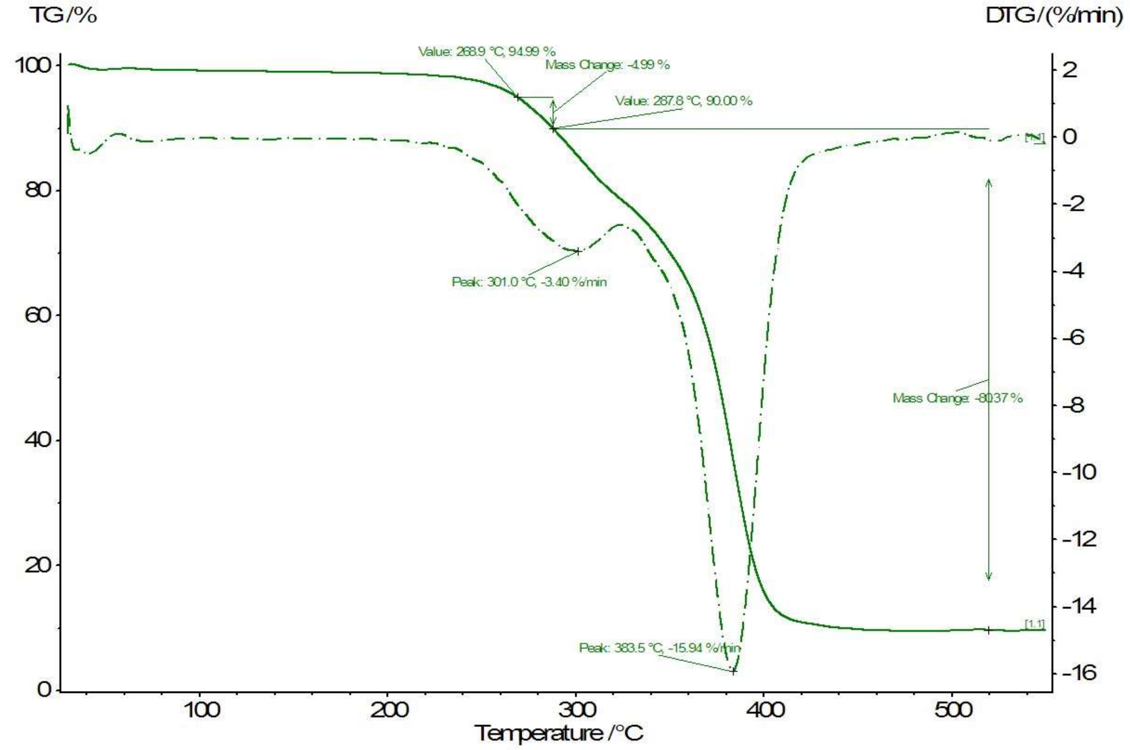

Figure S4g. TGA and DTG curves of the PU-M1.

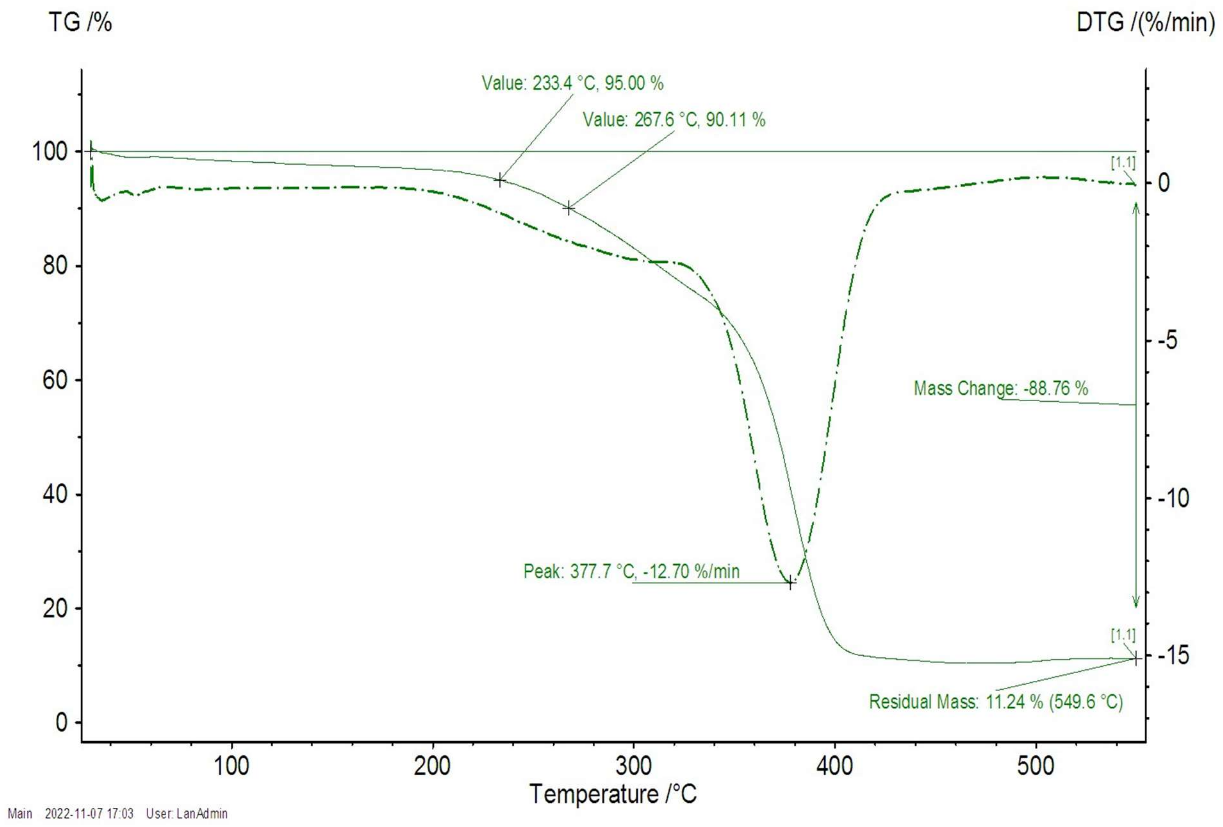

Figure S4h. TGA and DTG curves of the PU-M2.

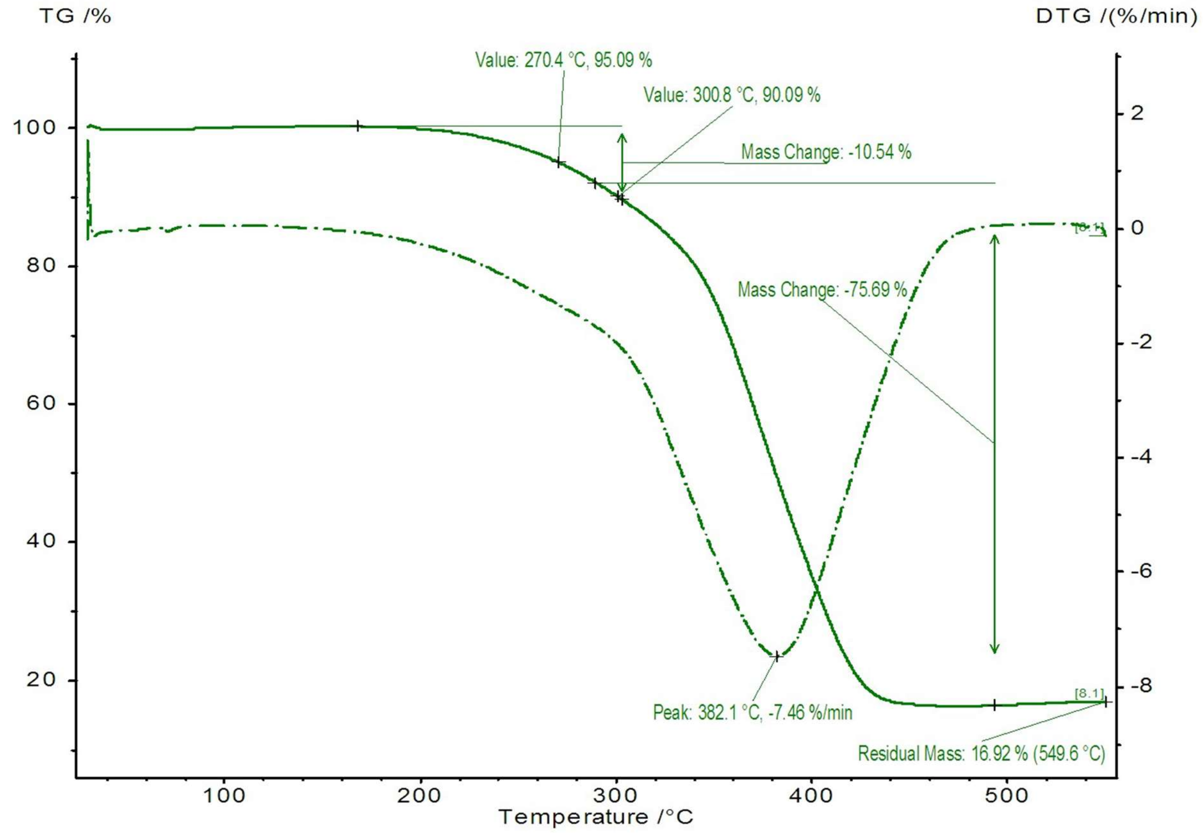

Figure S4i. TGA and DTG curves of the PU-M3.

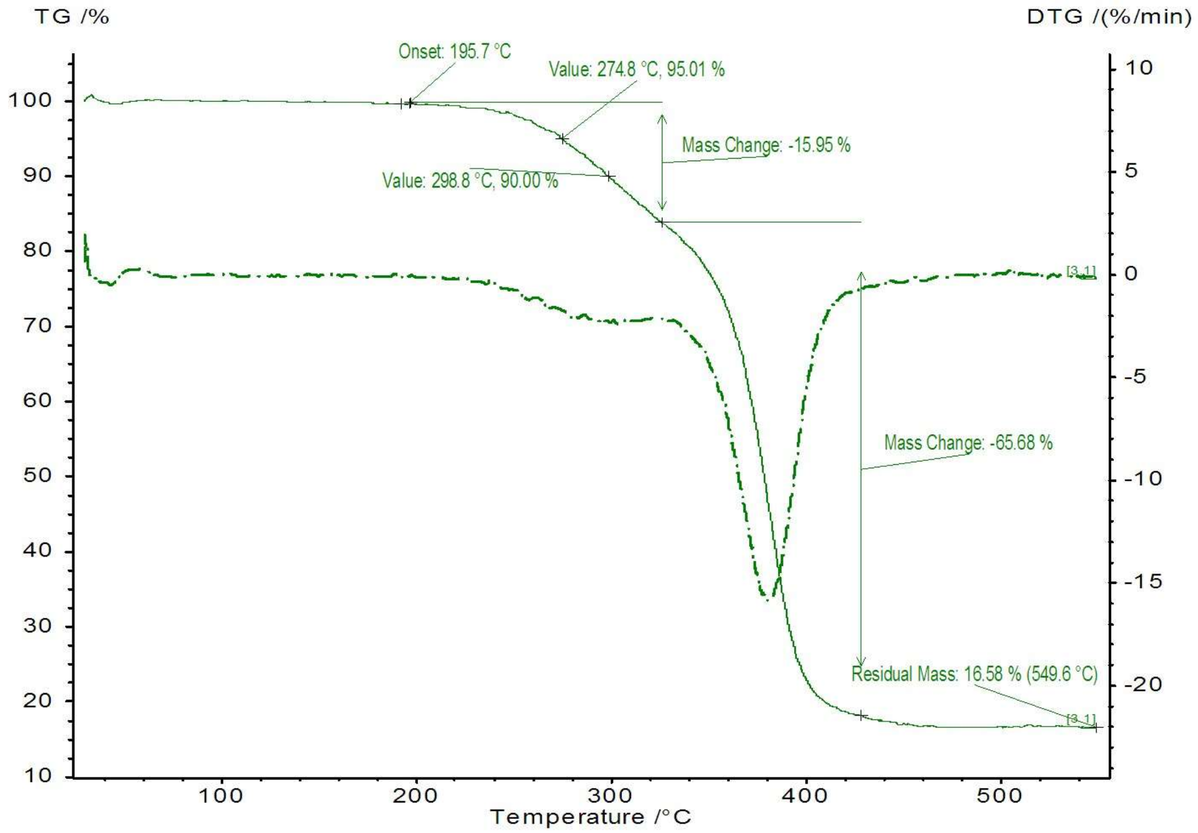

Figure S4j. TGA and DTG curves of the PU-$(NFu_2)_2$.

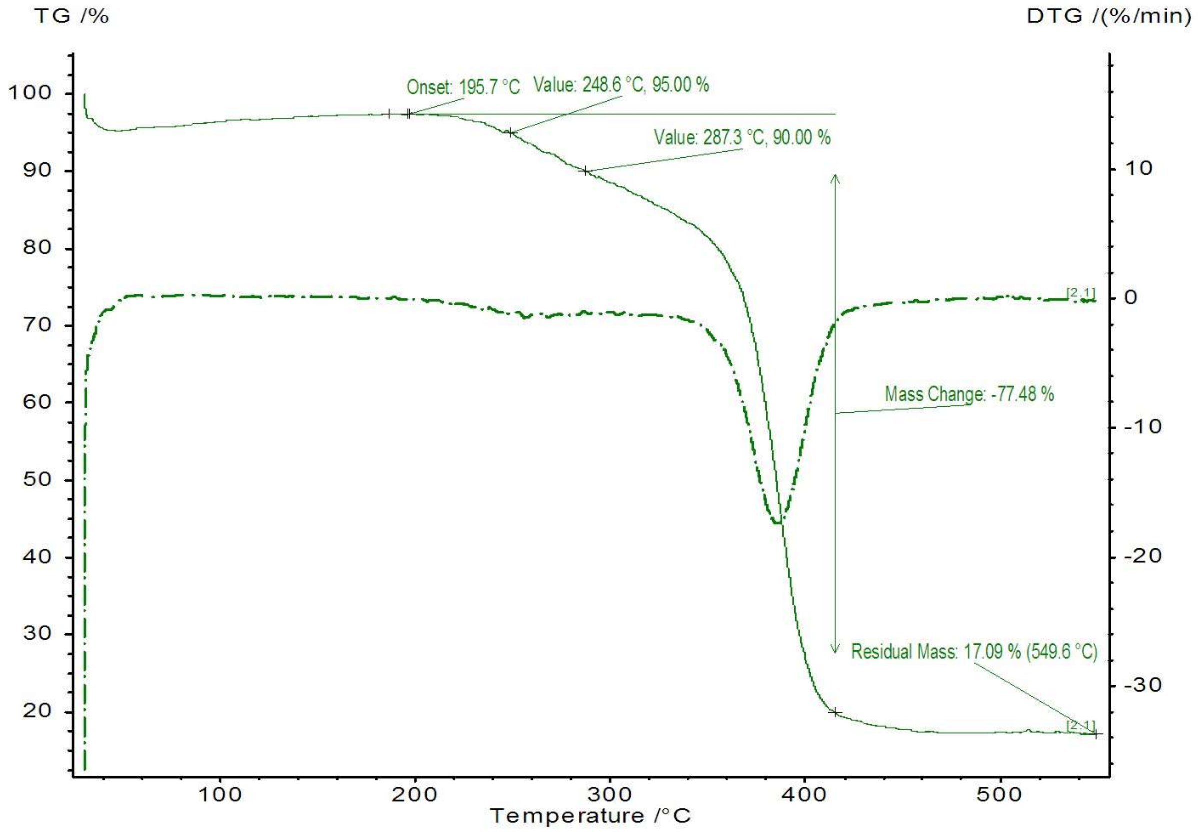

Figure S4k. TGA and DTG curves of the PU-$(NFu)_2$.

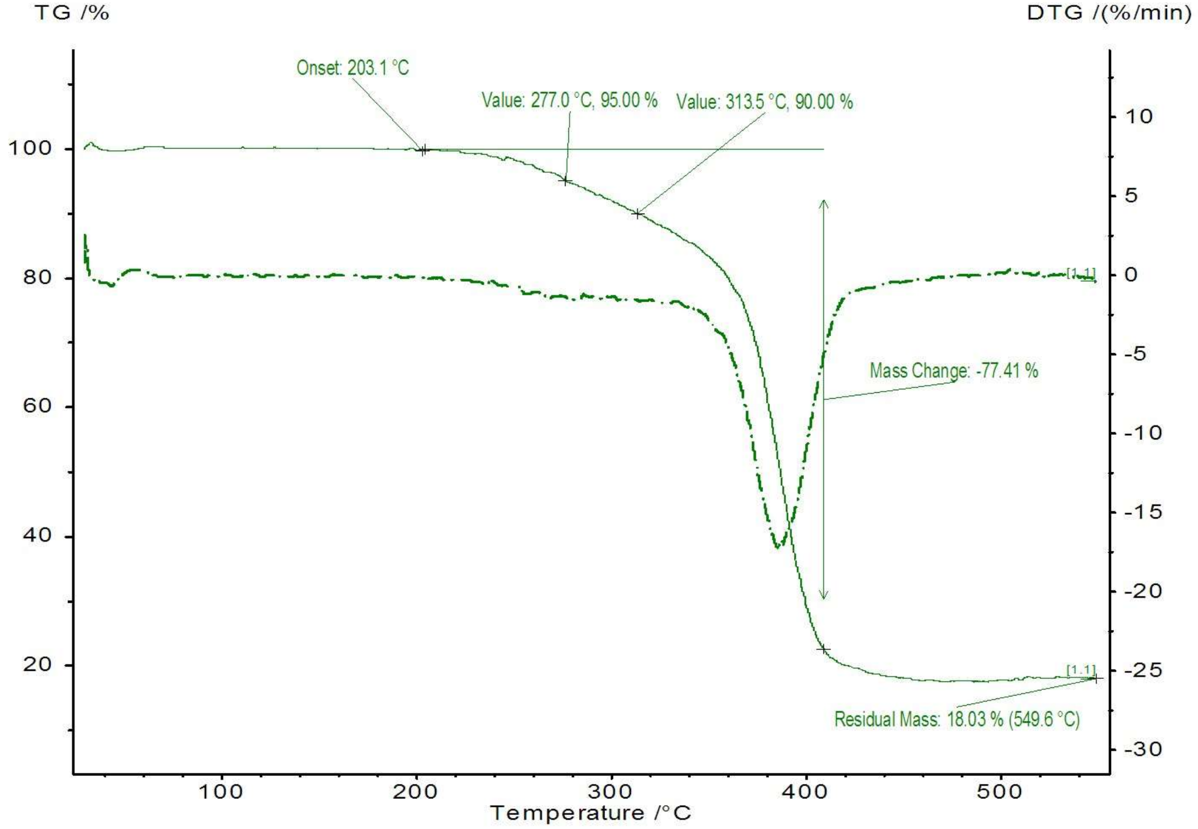

Figure S4l. TGA and DTG curves of the PU-$(OFu)_2$.

5 Differential Scanning Calorimetry (DSC)

The thermal behavior was examined by DSC, with a NETZSCH DSC 204 F1 Phoenix within a temperature range of the -80 to 180 °C at heating/cooling rates of the 10 K min^{-1} in argon atmosphere. A sample weight of the about 10-15 mg was used for measurement.

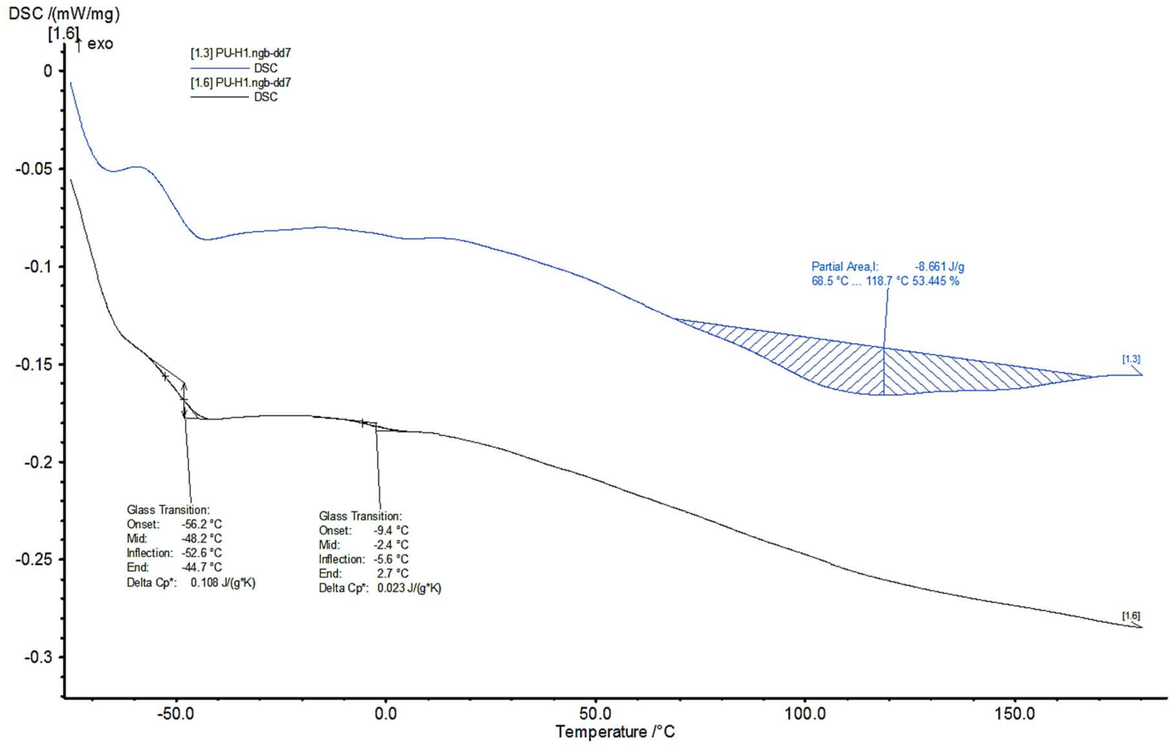

Figure S5a. DSC curve of the PU-H1 (the first and second heating curve).

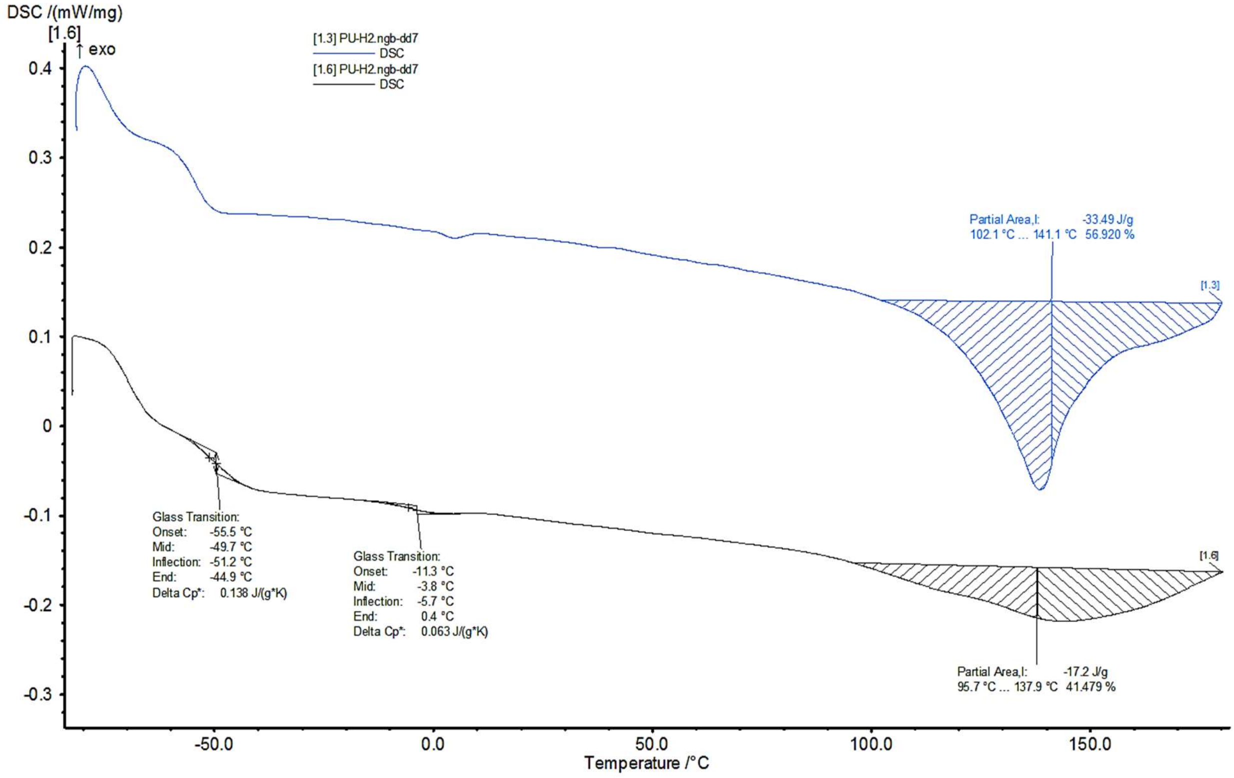

Figure S5b. DSC curve of the PU-H2 (the first and second heating curve).

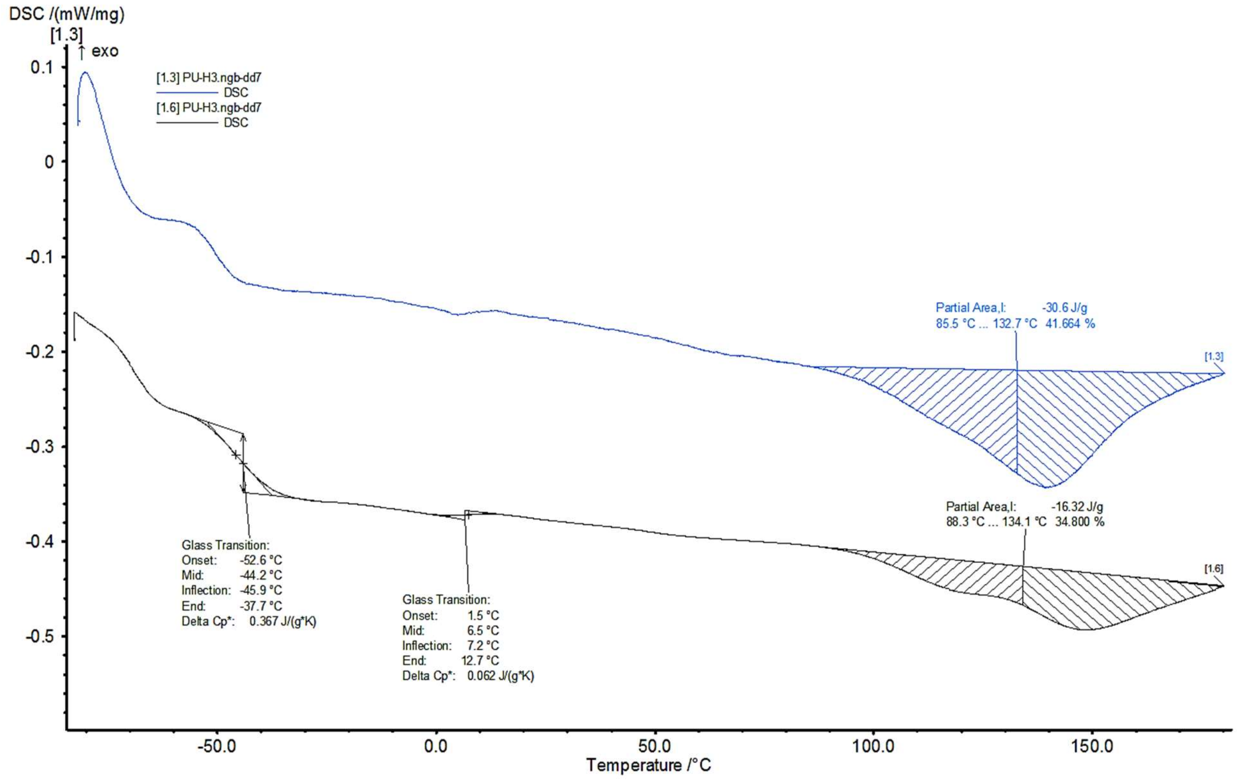

Figure S5c. DSC curve of the PU-H3 (the first and second heating curve).

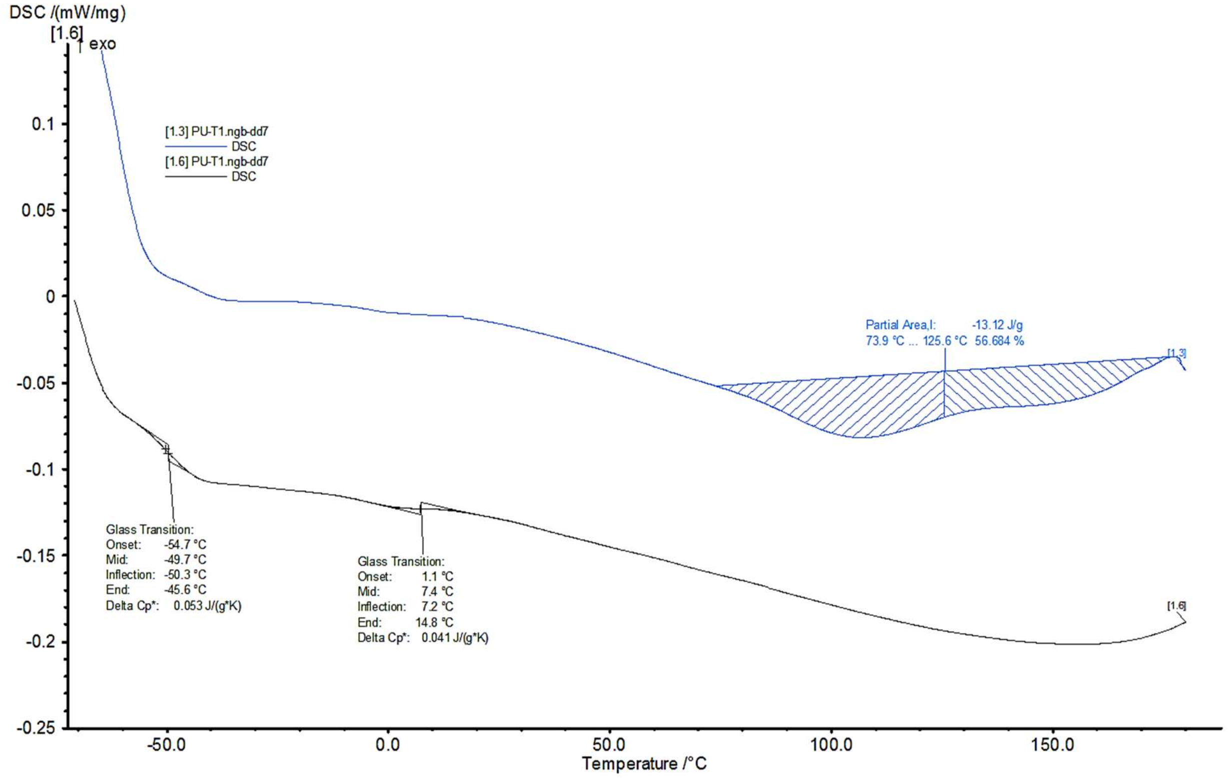

Figure S5d. DSC curve of the PU-T1 (the first and second heating curve).

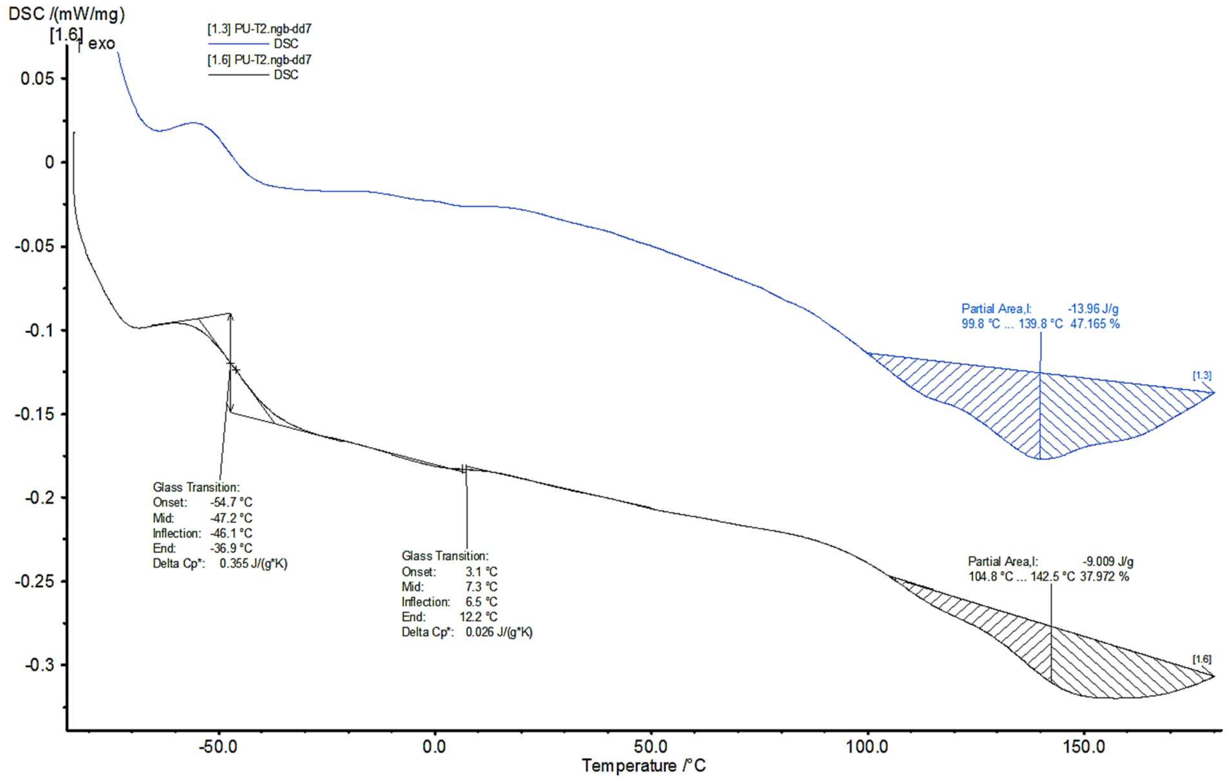

Figure S5e. DSC curve of the PU-T2 (the first and second heating curve).

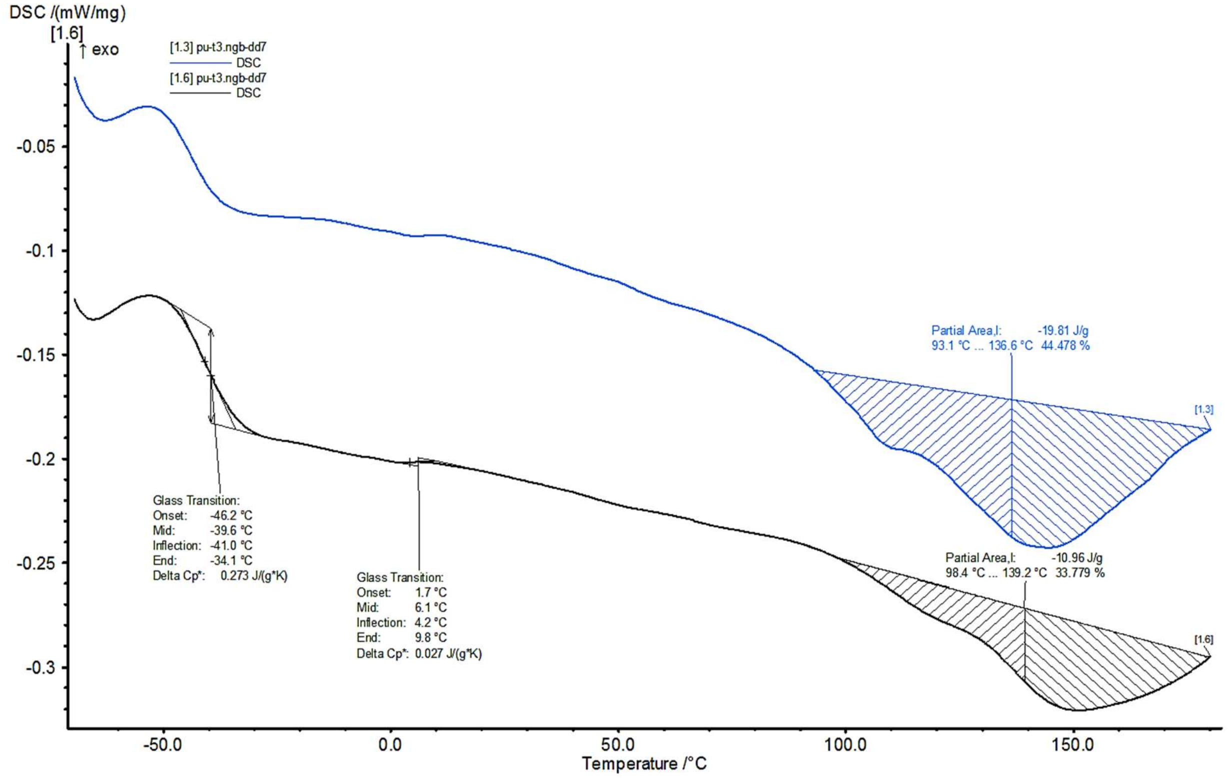

Figure S5f. DSC curve of the PU-T3 (the first and second heating curve).

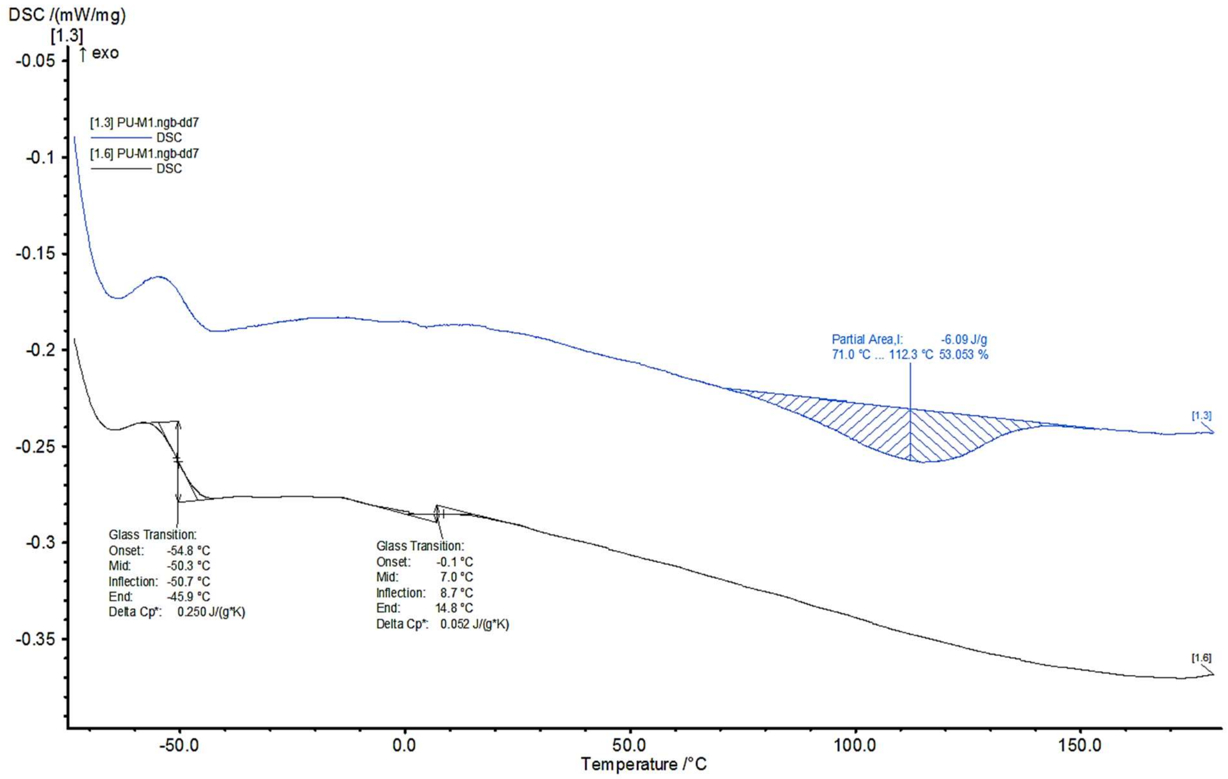

Figure S5g. DSC curve of the PU-M1 (the first and second heating curve).

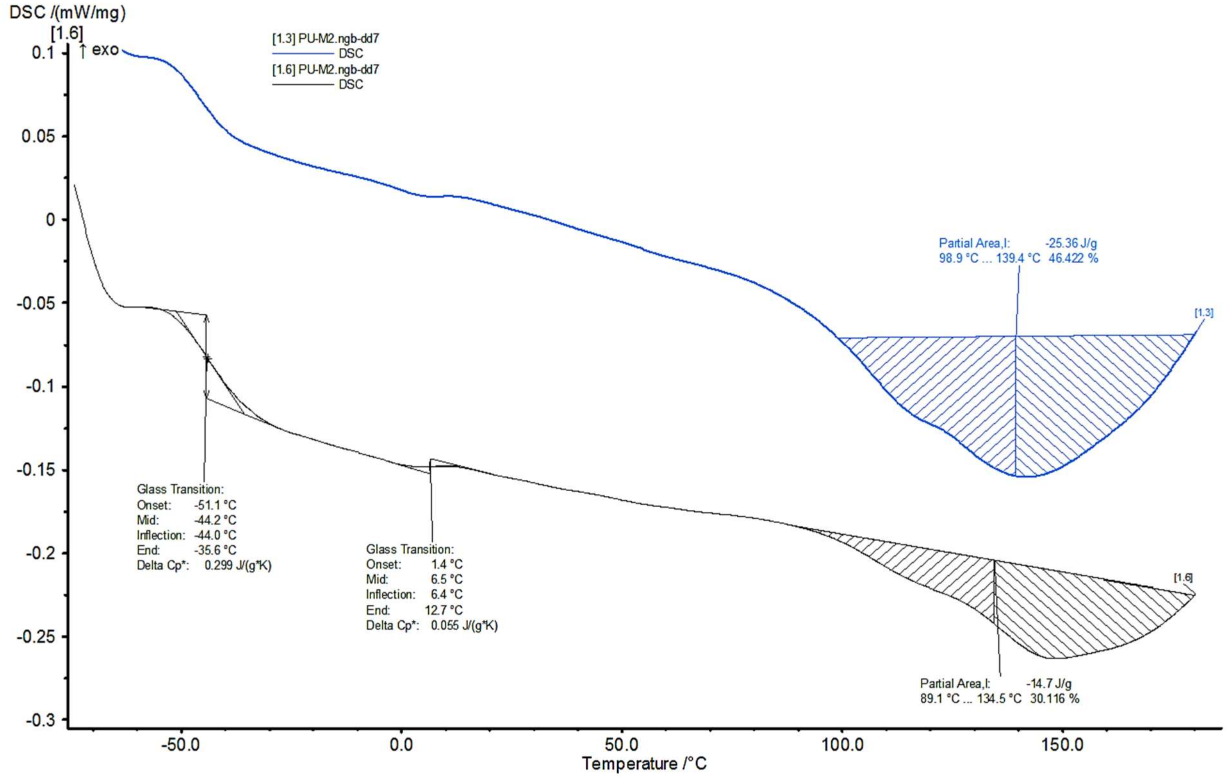

Figure S5h. DSC curve of the PU-M2 (the first and second heating curve).

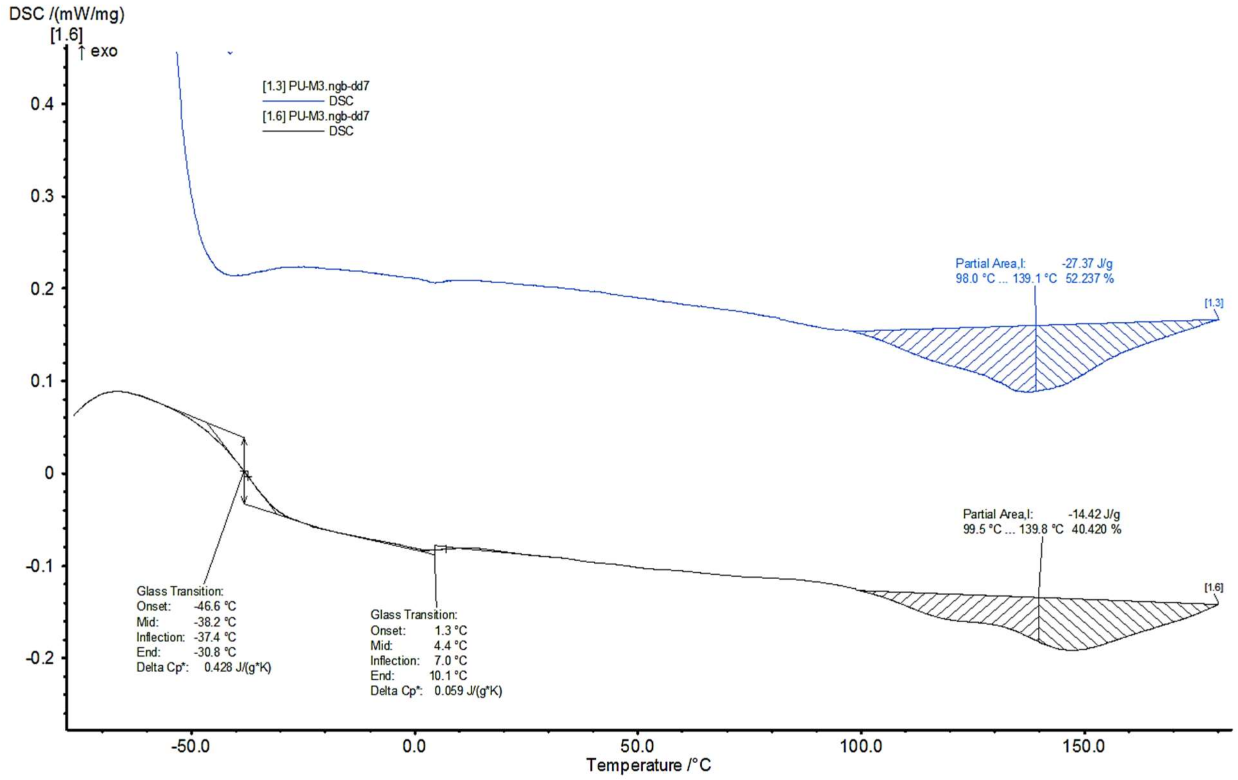

Figure S5i. DSC curve of the PU-M3 (the first and second heating curve).

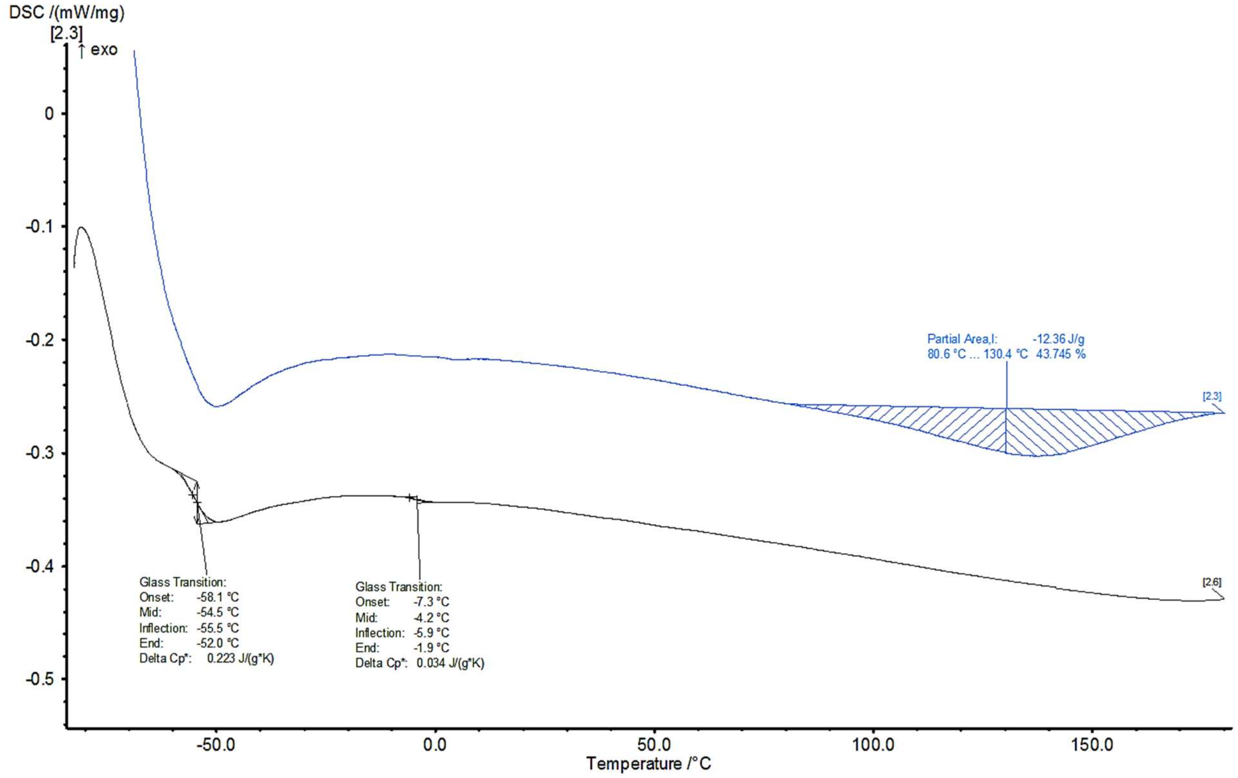

Figure S5j. DSC curve of the PU-$(NFu_2)_2$ (the first and second heating curve).

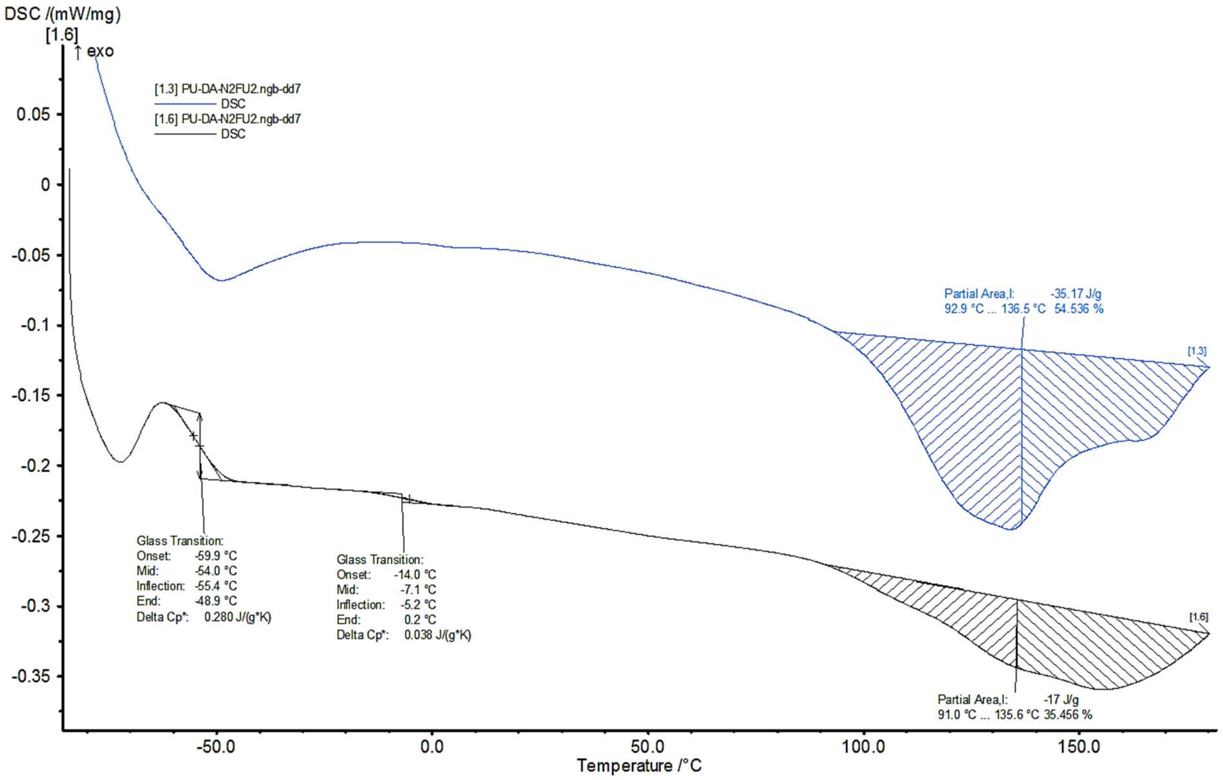

Figure S5k. DSC curve of the PU-$(NFu)_2$ (the first and second heating curve).

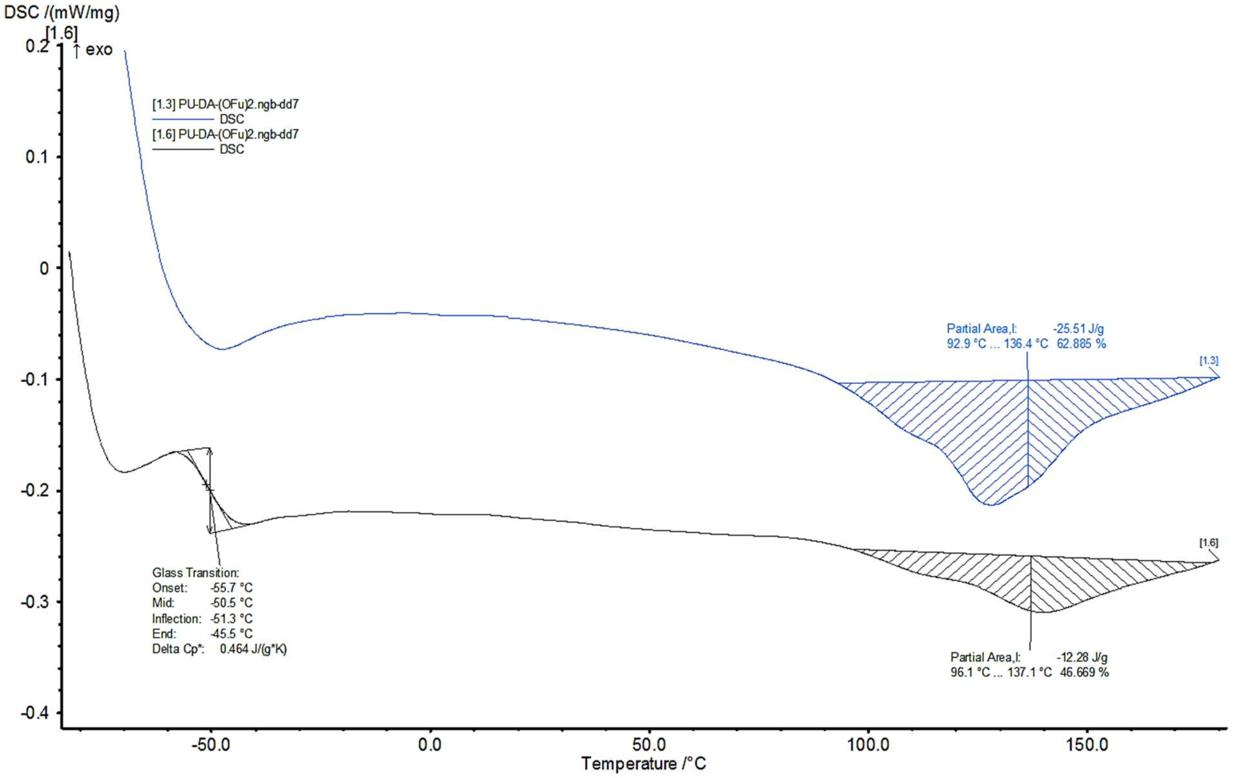

Figure S5l. DSC curve of the PU-$(OFu)_2$ (the first and second heating curve).

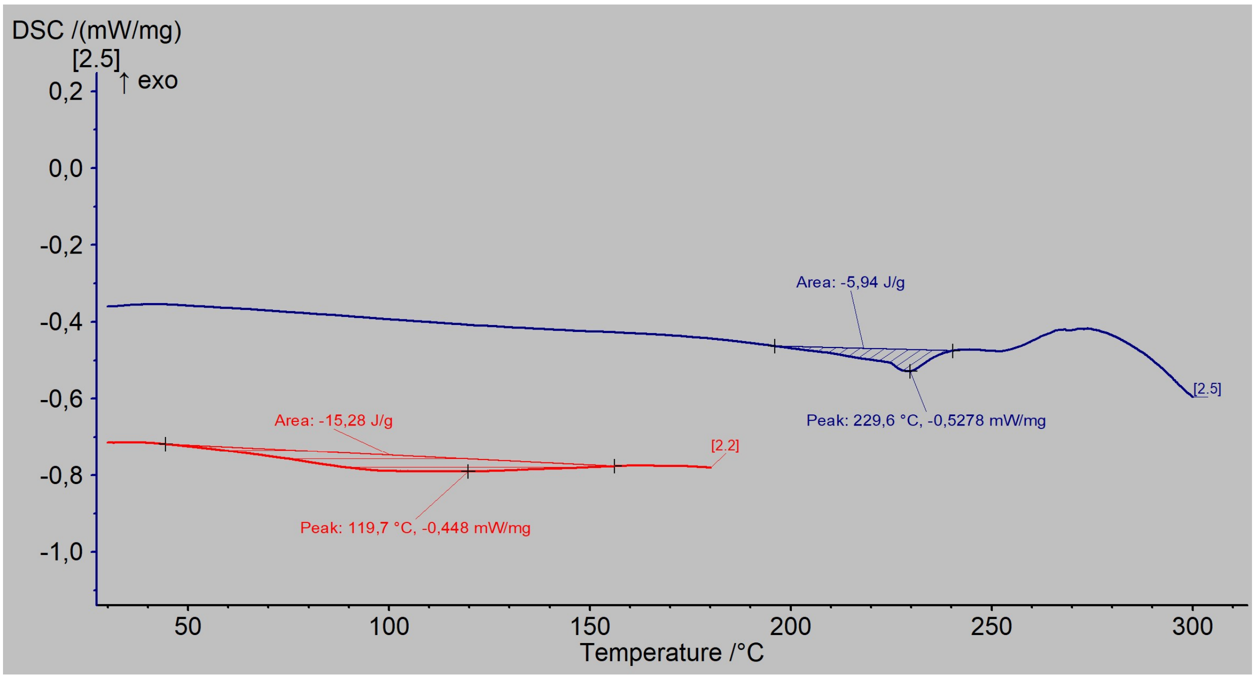

Figure S5m. Additional DSC curve of the PU-H1 (the first (red) and second heating curve(blue). 1st heating from 20 to 180 °C, 2nd heating from 20 to 300 °C

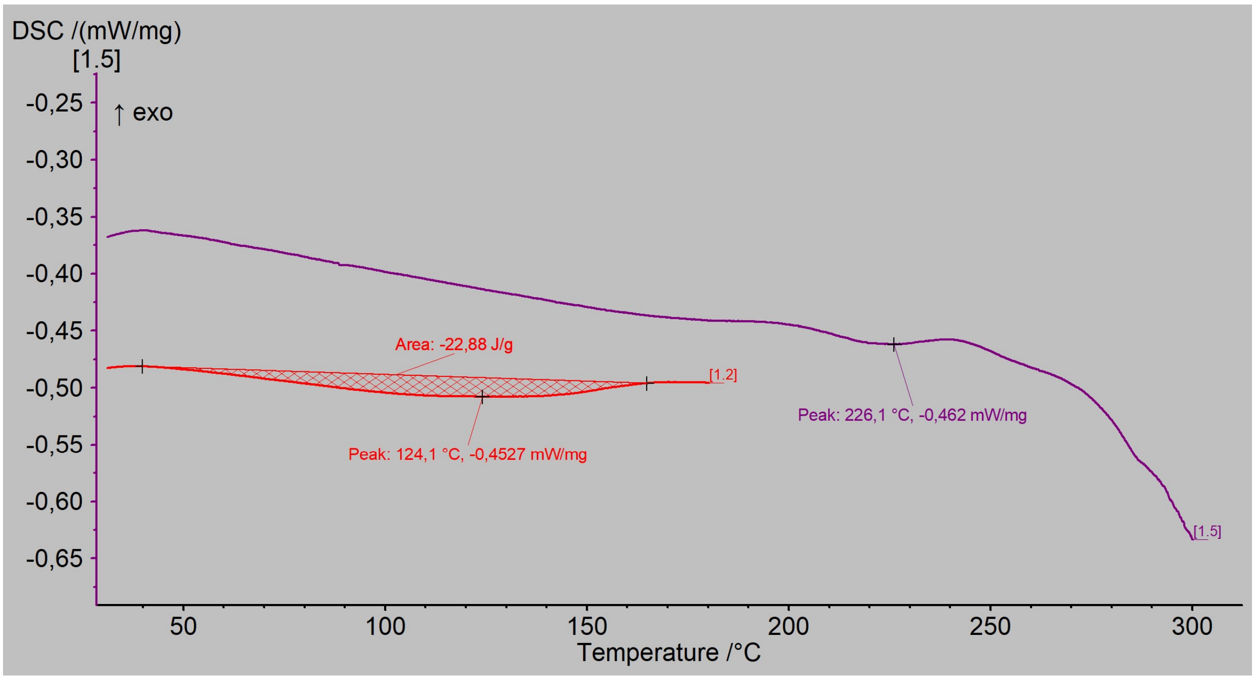

Figure S5n. Additional DSC curve of the PU-T1 (the first (red) and second heating curve(purple). 1st heating from 20 to 180 °C, 2nd heating from 20 to 300 °C

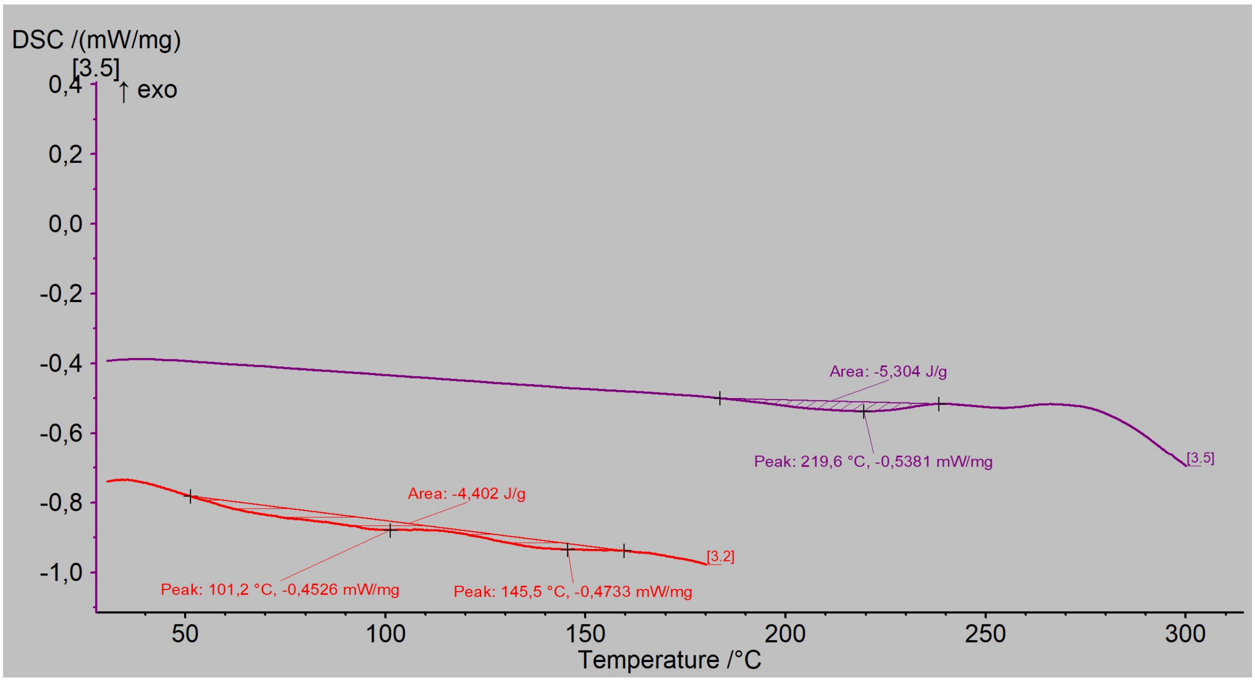

Figure S5o. Additional DSC curve of the PU-M1 (the first (red) and second heating curve(purple). 1st heating from 20 to 180 °C, 2nd heating from 20 to 300 °C

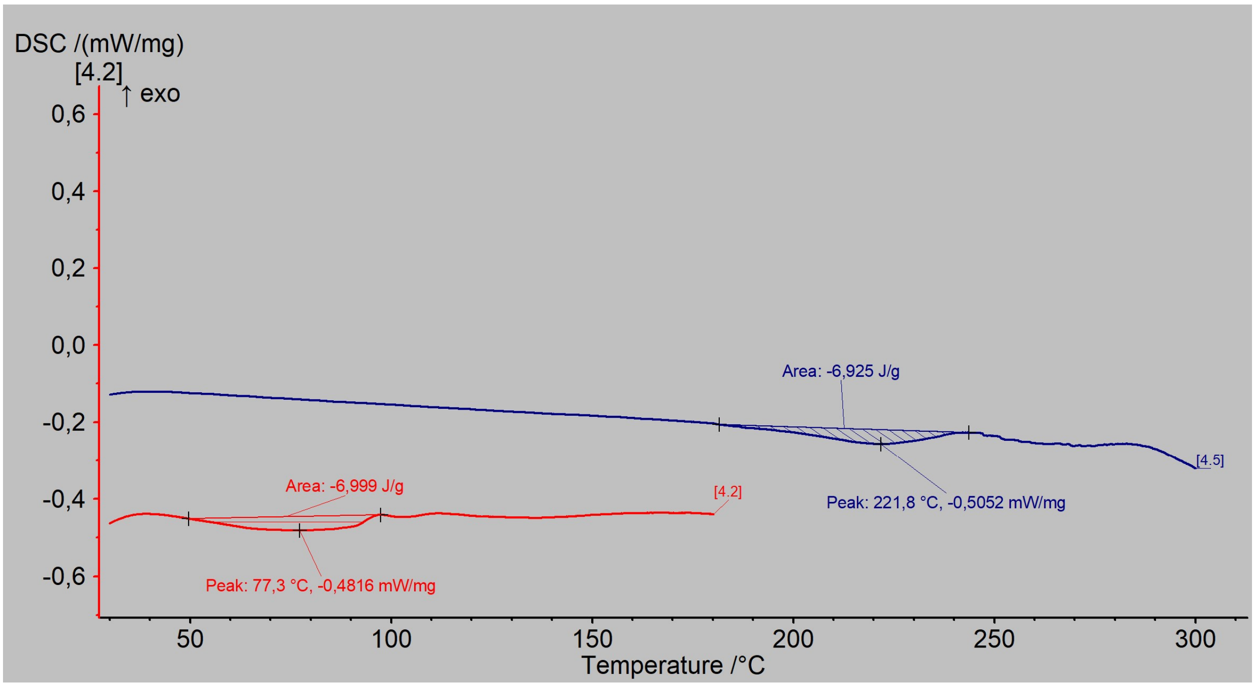

Figure S5p. Additional DSC curve of the PU-$(NFu_2)_2$ (the first (red) and second heating curve(BLUE). 1st heating from 20 to 180 °C, 2nd heating from 20 to 300 °C

5 Thermomechanical analysis (TMA)

Samples with a diameter of 6 mm were tested in the temperature range from -70 to 250 °C at heating/cooling rates of 5 K/min, a load of 1 N, and a probe diameter of 2.54 mm.

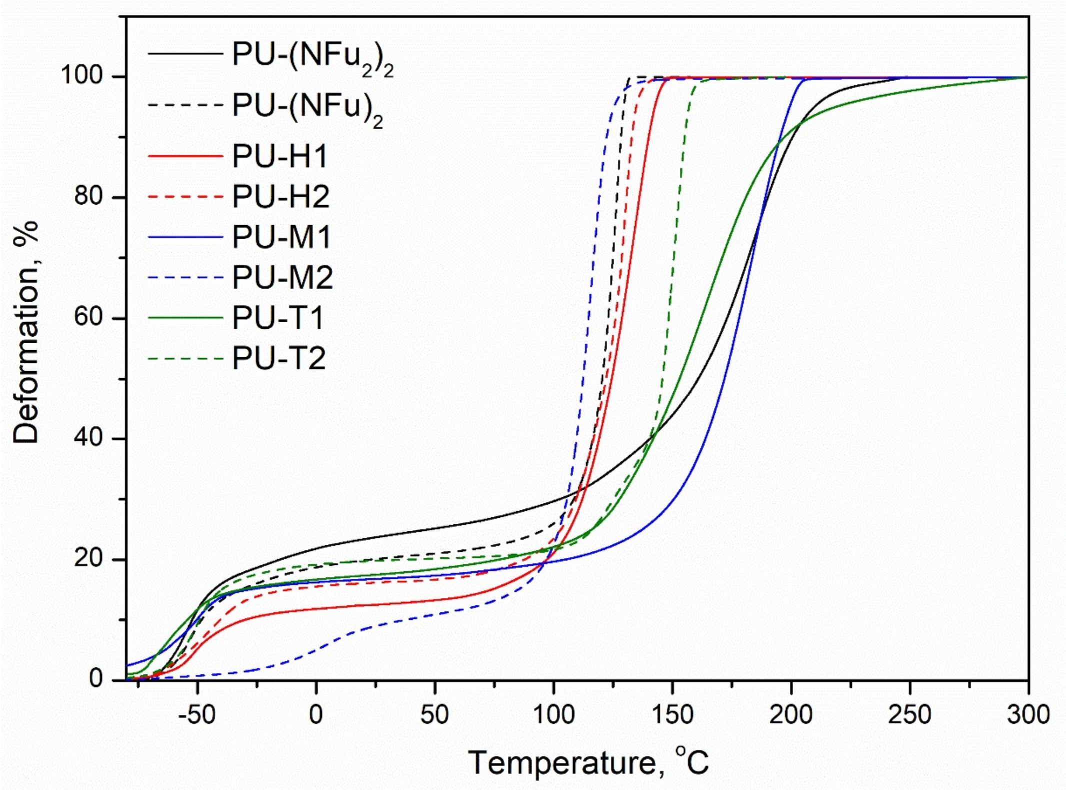

Figure S6a. TM curves of all polyurethanes.

References

[1] E. Platonova, P. Ponomareva, Z. Lokiaeva, A. Pavlov, V. Nelyub, A. Polezhaev, New Building Blocks for Self-Healing Polymers, Polymers, 14 (2022) 5394. https://doi.org/10.3390/polym14245394.
